# Intracranial Ependymoma: Long-Term Results in a Series of 21 Patients Treated with Stereotactic ^125^Iodine Brachytherapy

**DOI:** 10.1371/journal.pone.0047266

**Published:** 2012-11-05

**Authors:** Faycal El Majdoub, Moataz Elawady, Tobias Blau, Christian Bührle, Mauritius Hoevels, Matthias Runge, Rolf-Peter Müller, Martina Deckert, Volker Sturm, Mohammad Maarouf

**Affiliations:** 1 Department of Stereotaxy and Functional Neurosurgery, University Hospital, Cologne, Germany; 2 Department of Neuropathology, University Hospital, Cologne, Germany; 3 Department of Radiation Oncology, University Hospital, Cologne, Germany; Johns Hopkins University, United States of America

## Abstract

**Background:**

We evaluated the long-term outcome in patients harboring intracranial ependymomas treated with interstitial brachytherapy (IBT).

**Methods:**

Twenty-one patients (M/F = 9/12; median age: 29 years; range: 8–70 years), diagnosed with intracranial ependymoma (1 WHO I, 11 WHO II, 9 WHO III) were treated with IBT using stereotactically implanted ^125^Iodine seeds between 1987 and 2010, either primarily, as adjuvant therapy following incomplete resection, or as salvage treatment upon tumor recurrence. Sixteen of 21 patients underwent microsurgical resection prior to IBT; in 5 patients, IBT was performed primarily after stereotactic biopsy for histological diagnosis. The cumulative tumor surface dose ranged from 50–65 Gy treating a median tumor volume of 3.6 ml (range, 0.3–11.6 ml). A median follow-up period of 105.3 months (range, 12.7–286.2 months) was evaluated.

**Results:**

Actuarial 2-, 5- and 10-years overall- and disease-specific survival rates after IBT were each 90% and 100% at all times for ependymomas WHO I/II, for anaplastic ependymomas WHO III 100%, 100%, 70% and 100%, 100%, 86%, respectively. The neurological status of seven patients improved, while there was no change in 12 and deterioration in 2 patients, respectively. Follow-up MR images disclosed a complete tumor remission in 3, a partial remission in 12 and a stable disease in 6 patients. Treatment-associated morbidity only occurred in a single patient.

**Conclusions:**

This study shows that stereotactic IBT for intracranial ependymomas is safe and can provide a high degree of local tumor control. Due to the low rate of side effects, IBT may evolve into an attractive alternative to microsurgery in ependymomas located in eloquent areas or as a salvage treatment.

## Introduction

Ependymomas are rare tumors arising from ependymal cells lining the ventricular system, from the central canal of the spinal cord, and from the filum terminale. They account for 6–12% of all intracranial tumors in children, even constituting up to 50% in patients younger than 5 years of age [1], [2]. Almost 90% of pediatric ependymomas are located intracranially, with two thirds arising within the posterior fossa [3], [4]. In adults, ependymomas account for approximately 4% of CNS tumors and for 50–60% of neuroepithelial tumors in the spinal cord [1], [5], respectively.

Histopathologically, the tumors either correspond to classic ependymomas (WHO II), anaplastic ependymomas (WHO III), or subependymomas (WHO I) [6].

Optimum therapy of intracranial ependymoma is still controversial varying among institutions from surgery alone to a multimodal treatment consisting of surgery, radio- and chemotherapy [1], [7]. Older age at diagnosis and extent of surgery have both been identified as favorable prognostic factors reported most frequently, even though local recurrence after complete resection may occur in up to 50% of patients [2], [8], [9]. Even late relapses after 10 to 15 years are not uncommon [10]. The overall- and progression-free survival rates after 5 years range between 39–64% and 23–45%, respectively [11]. At the molecular biological level, gains of chromosome 1q have been emphasized as an adverse prognostic factor [8]. Also the determination of valid biomarkers may predict the clinical behavior and prognosis of ependymomas [12].

In this study, we evaluated the long-term outcome in patients suffering from intracranial ependymoma treated with interstitial brachytherapy (IBT) using stereotactically implanted ^125^Iodine seeds (^125^I).

## Patients and Methods

### Ethics Statement

No separate ethics application and statement by the ethical committee for this retrospective study are required. This study has been evaluated in accordance with German data protection legislation (Statement of the Ethic Committee, German Data Protection Legislation English Version and Data Protection Legislation available as Files S1, S2, S3). This, in particular, means that the results of the study have been obtained in a completely anonymous manner.

For all kinds of treatment done at the Department of Stereotaxy and Functional Neurosurgery Cologne it is mandatory to obtain informed written consent of patients scheduled for treatment. In case of minors, this consent is granted either by their parents or by a court-approved caregiver.

Twenty-one patients with intracranial ependymoma were treated between February 1987 and August 2010 (Table 1). All patients (M/F ratio: 9/12; median age: 29 years, range: 8–70 years) had a follow-up of at least 12.7 months and were evaluated as follows:

**Table 1:** Patient characteristics and treatment parameters.

	Gender	Age at IBT	Therapy prior IBT	Grade	Tumor volume (ml)	Dose (Gy)	Time (Days)	Therapy post IBT	Response	Survival (months)
1.	M	22	None	III	3.5	50	42	Rx(36Gy)	PR	286.2
2.	F	28	TR	II	6.0	65	Perm.	None	PR	214.8
3.	F	41	TR, Rx(36Gy)	III	3.2	50	90	None	PR	208.9
4.	F	21	TR	II	4.2	65	Perm.	None	PR	188.2
5.	F	35	STR, Cx(HIT91), Rx(36Gy)	III	3.8	50	Perm.	None	Stable	171.5
6.	M	29	TR, Rx(36Gy)	III	2.1	50	90	None	Stable	91.3*
7.	F	14	TR, Cx(HIT91)	II	1.9	65	Perm.	None	PR	155.9
8.	F	35	STR, Rx(36Gy)	II§	11.6	50	90	None	Stable	138.6
9.	F	24	TR, Cx(HIT91), Rx(30.5+10Gy#)	III	5.0	50	42	None	PR	62.2*
10.	F	15	None	II	3.6	65	Perm.	None	PR	137.1
11.	M	44	STR	II	4.0	65	Perm.	None	PR	109.7
12.	F	17	TR, Cx(HIT2001)	II	2.1	65	Perm.	None	CR	107.5
13.	F	13	TR	II	4.0	65	Perm.	None	CR	105.3
14.	F	8	STR, Cx(HIT2001)	III	1.8	50	42	Rx(36Gy)	Stable	80.6
15.	M	29	TR, Rx(36Gy)	III	4.5	50	42	None	PR	65.5
16.	M	40	TR	II	4.5	65	Perm.	None	PR	64.3
17.	M	36	None	III	4.2	50	42	Rx(36Gy)	CR	60.2
18.	M	48	None	I	3.1	50	Perm.	None	PR	12.1*
19.	M	37	TR, Rx(36Gy)	III	2.4	50	42	None	PR	34.6
20.	F	70	TR	II	3.6	50	Perm.	None	Stable	27.0
21.	M	49	None	II	0.3	50	Perm.	None	Stable	12.7

Abbreviations: IBT, interstitial brachytherapy; ml, milliliter; Gy, Gray; TR, total resection; STR, subtotal resection; Rx, radiation therapy; Cx, chemotherapy; Perm., permanent; PR, partial response; CR, complete response.

# Pat. received 30.5 Gy to the brain and additional 10 Gy to the spinal metastasis,

§ Neuropathology confirmed Grade II instead of III on re-evaluation,

* Pat. died.

The cohort presented here included 5 patients less than 18 years: i.e. aged 8, 13, 14, 15 and 17 years, respectively. At the time of IBT, patients presented with headache (15/21), diplopia (8/21), vertigo (4/21), hemiparesis (2/21) and seizures (1/21). The tumors were located in the lateral ventricles (9/21), fourth ventricle (5/21), third ventricle (2/21), brainstem (2/21), cerebellopontine angle (1/21), basal ganglia (1/21), and medulla oblongata (1/21). The M-stage was M0 in 19 patients, M1 in one patient, and M3 in an additional one who presented evidence for spinal metastasis at the level of S1 in MR images. In both patients, the tumor was diagnosed as anaplastic ependymoma (WHO III).

### Pretreatments

Prior to stereotactic interstitial brachytherapy, a ventriculoperitoneal shunt was placed in 5 of 21, while a ventriculo-cisternotomy was performed in 2 of 21 patients with occlusive hydrocephalus to remove CSF flow obstruction. Sixteen patients had undergone microsurgical resection at different institutions (12 total, 4 subtotal resections). The remaining five patients had had stereotactic biopsies for validating the neuropathological diagnosis. As an adjuvant to microsurgery 10 patients received further treatment consisting of external beam irradiation (EBI) alone (5 patients, each with a total dose of 36 Gy), chemotherapy alone (3 patients, according to HIT 91 and 2001) and combined radio-chemotherapy (2 patients, total dose of 30.5 Gy and 36 Gy combined with chemotherapy according to HIT 91). The single patient with M3 status additionally received 10 Gy for the spinal metastasis. The median tumor volume was 3.6 ml (range 0.3–11.6 ml).

### Neuropathology

Prior to IBT neuropathology diagnosed ependymoma WHO II in 10 patients, while 10 tumors were classified as anaplastic ependymoma WHO III; one tumor corresponded to a subependymoma WHO I. Neuropathological re-evaluation, however, revealed ependymoma WHO II in 10 patients, anaplastic ependymoma (WHO III) in 5 patients and a subependymoma in 1 patient (Table 2). In 5 cases, no tumor specimen was available for a second histopathological evaluation. These tumors had initially been diagnosed as ependymoma WHO II (n = 1) and WHO III (n = 4), respectively (Table 2). In one patient (patient #8 on tables 1 and 2), neuropathological re-examination downgraded the initial diagnosis from anaplastic ependymoma (WHO III) to ependymoma WHO II.

**Table 2:** Neuropathological re-evaluation of the biopsy specimens obtained from the patients of this study.

	Gender	Age at IBT	Initial diagnosis	Diagnosis after re-evaluation
1.	M	22	anaplastic ependymoma (WHO III)	n.a.
2.	F	28	ependymoma (WHO II)	n.a.
3.	F	41	anaplastic ependymoma (WHO III)	anaplastic ependymoma (WHO III)
4.	F	21	ependymoma (WHO II)	ependymoma (WHO II)
5.	F	35	anaplastic ependymoma (WHO III)	anaplastic ependymoma (WHO III)
6.	M	29	anaplastic ependymoma (WHO III)	anaplastic ependymoma (WHO III)
7.	F	14	ependymoma (WHO II)	ependymoma (WHO II)
8.	F	35	anaplastic ependymoma (WHO III)	ependymoma (WHO II)
9.	F	24	anaplastic ependymoma (WHO III)	anaplastic ependymoma (WHO III)
10.	F	15	ependymoma (WHO II)	ependymoma (WHO II)
11.	M	44	ependymoma (WHO II)	ependymoma (WHO II)
12.	F	17	ependymoma (WHO II)	ependymoma (WHO II)
13.	F	13	ependymoma (WHO II)	ependymoma (WHO II)
14.	F	8	anaplastic ependymoma (WHO III)	anaplastic ependymoma (WHO III)
15.	M	29	anaplastic ependymoma (WHO III)	anaplastic ependymoma (WHO III)
16.	M	40	ependymoma (WHO II)	ependymoma (WHO II)
17.	M	36	anaplastic ependymoma (WHO III)	anaplastic ependymoma (WHO III)
18.	M	48	subependymoma (WHO I)	subependymoma (WHO I)
19.	M	37	anaplastic ependymoma (WHO III)	anaplastic ependymoma (WHO III)
20.	F	70	ependymoma (WHO II)	ependymoma (WHO II)
21.	M	49	ependymoma (WHO II)	ependymoma (WHO II)

Abbreviations: IBT, interstitial brachytherapy; n.a., not available.

### Eligibility

All patients were treated with IBT using stereotactic computed tomography and magnetic resonance imaging as a basis for stereotactical planning and guiding ^125^I seed implantation as either primary (n = 5), adjuvant (n = 4) or salvage (n = 12) therapy.

In our interdisciplinary tumor board to which stereotactic neurosurgeons, general neurosurgeons, neurologists, neuroradiologists, radiotherapists, pediatricians and neuropathologists contribute, we recommended that treatment of intracranial ependymoma should depend on a combination of criteria, including a) pretreatment (radiotherapy), b) tumor spread and tumor size (tumor size >4 cm = exclusion for IBT), c) microsurgical accessibility, d) patients' operability (multimorbidity) and e) patient refusal of microsurgery. Based on this information, the majority of patients in our institution were treated by microsurgery and radiotherapy.

### Technical data and surgical procedure

Analogous to our treatment schedule for gliomas [13], [14], also ependymomas of all WHO grades were permanently implanted. After radiotherapy recurrent ependymomas underwent either permanent or temporary implantation (median 42 days, range 42–90 days). Adjuvant fractionated radiotherapy was applied in patients with anaplastic ependymoma (WHO III) (whole brain, 36 Gy, 1.8–2 Gy/d) who did not receive any external radiotherapy prior to IBT. The detailed patients' characteristics and treatment parameters are displayed in table 1.

**Figure 1:**
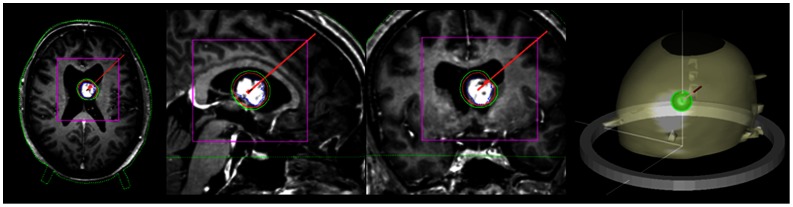
Precise treatment planning on axial, sagittal and coronal T_1_-weighted, gadolinium enhanced MR images (blue dotted line: tumor border; red line: 65 Gy isodose; green line: 50 Gy isodose) and 3D-trajectory plan (**figure 1d**).

^125^I seeds (Amersham Buchler GmbH & Co KG) were used in both permanent and temporary implants. In patients being permanently implanted, a cumulative tumor surface dose of 50–65 Gy was applied. Patients implanted temporarily were irradiated with a total dose of 50 Gy. Biopsy and implantation were done under general anesthesia, using a modified Riechert-Mundinger stereotactic frame [15]. Stereotactic 3D treatment was planned by the stereotactic neurosurgeon using STP 3.5 software (until February 1996 with STP 2; Leibinger, Freiburg, Germany) supported by a medical physicist. Entry points and targets of the catheters were determined taking into account both, optimum dose distribution and safest trajectory. In most cases one catheter and two seeds were implanted (range, 1–3 catheters and 2–6 seeds). The therapeutic isodose curve was superimposed on the tumor contour defined on MR images and stereotactic CT (Figure 1).

During autoclavation of the seeds, the stereotactic device was set up and the burr hole drilled. An outer nylon catheter (outside diameter 2.0 mm; BEST Industries, Inc., Springfield, VA) was positioned stereotactically and loaded with an inner catheter where the ^125^I seeds had been placed previously. After verification of catheter and seed location by orthogonal stereotactic X-ray, both catheters were fixed in the burr hole with Palacos® (Heraeus Medical GmbH, Wehrheim, Germany) and the skin sutured [13], [14], [16].

### Follow-up

After IBT, clinical follow-up data were obtained from patients and referring physicians for a median period of 105.3 months (range 12.7–286.2 months). MR images from patients with ependymoma WHO I and II were requested at 6-month intervals during the first 3 years and at 1-year intervals thereafter. In patients with anaplastic ependymoma (WHO III), MR images were scheduled for 3-month intervals during the first year and for 6-month intervals subsequently. Tumor response was classified according to MacDonald [17].

## Results

All of the 26 stereotactically guided procedures (21 ^125^I seed implantations and 5 biopsies) were performed without perioperative complications.

### Ependymoma WHO I/II (12 patients)

**Figure 2:**
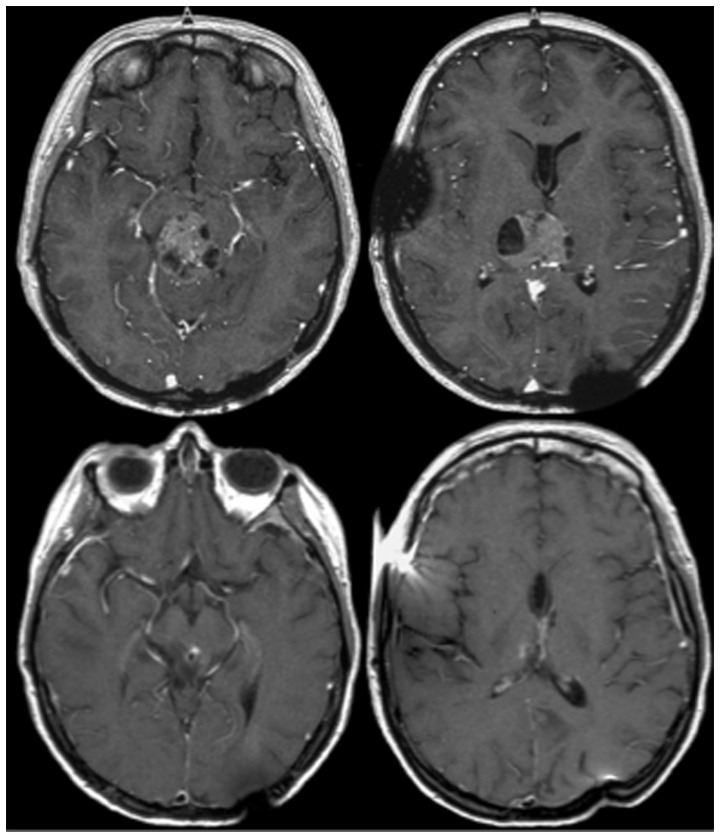
Follow-up MRI of a 14-year old female with an ependymoma III in the midbrain prior to IBT (upper line) and partial tumor remission 13 years after treatment (bottom line).

After a median clinical follow up of 109.7 months (range 12.7–214.8 months) 3 patients (25%) were free of symptoms. In the remaining 9 patients (75%) the symptoms improved or remained stable. There was no treatment-related mortality in this group. Two patients exhibited transient treatment-related deficits; one patient had a slight right-sided hemiparesis (tumor located in the left basal ganglia), and one patient complained of nausea (tumor within the brainstem). Symptoms recovered completely upon steroid medication within one week. One patient developed left-sided facial nerve palsy grade III according to House & Brackmann three months after treatment (second patient implanted in 1993, tumor located in the fourth ventricle, Table 3). One patient died due to myocardial infarction 12.1 months after interstitial brachytherapy after a partial response of the treated tumor. MR images showed a complete local remission in 16.7% (2/12), a partial remission in 58.3% (7/12) and stable disease in the remaining 25% (3/12). Representative cases were shown in figure 2 and figures S1 and S2. No tumor progression was documented in this group.

**Table 3:** Permanent and temporary side effects after IBT (n = 4/21).

	Gender	Age at IBT	Therapy prior IBT	Grade	Dose (Gy)	Time	Therapy post IBT	Side effects	Recovery
1.	F	28	TR	II	65	Perm.	None	N.VII palsy (H&B III)	Perm.
2.	F	14	TR, Cx(HIT91)	II	65	Perm.	None	Hemiparesis	6 Days
3.	F	15	None	II	65	Perm.	None	Nausea	7 Days
4.	M	29	TR, Rx(36Gy)	III	50	90 Days	None	Cognitive deficits	Perm.

Abbreviations: IBT, interstitial brachytherapy; Gy, Gray; TR, total resection; Rx, radiation therapy; Cx, chemotherapy; Perm., permanent; H&B, House & Brackmann.

**Figure 3:**
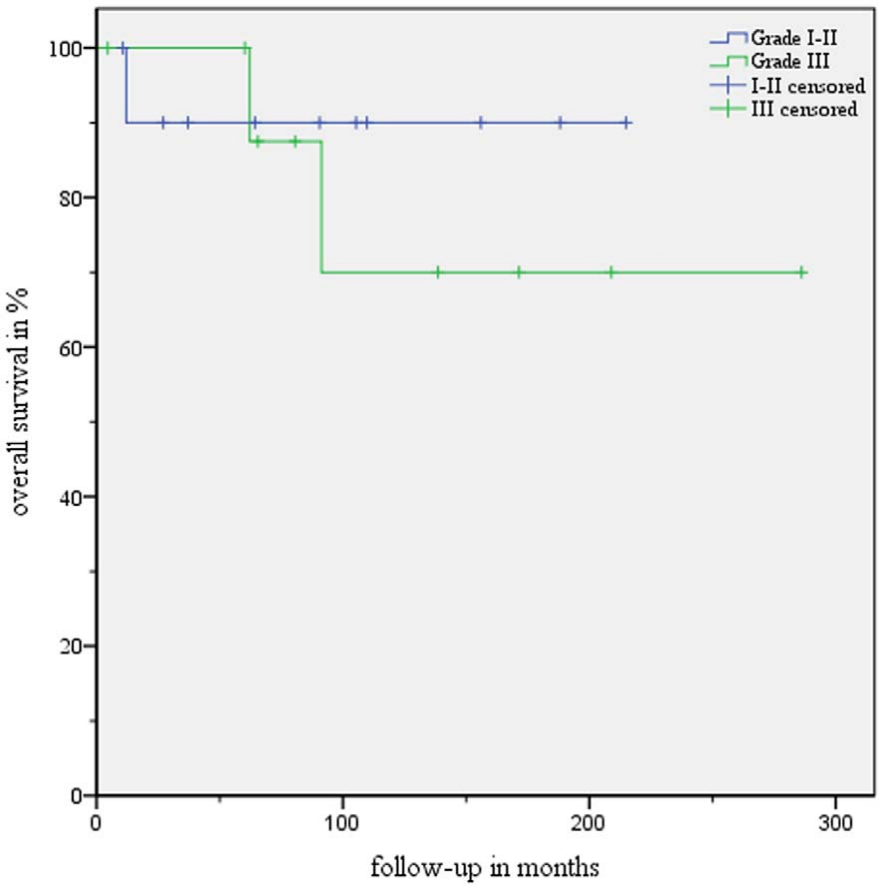
Overall survival curves according to Kaplan-Meier for ependymoma WHO I and II (blue graph) and anaplastic ependymoma WHO III (green graph).

**Figure 4:**
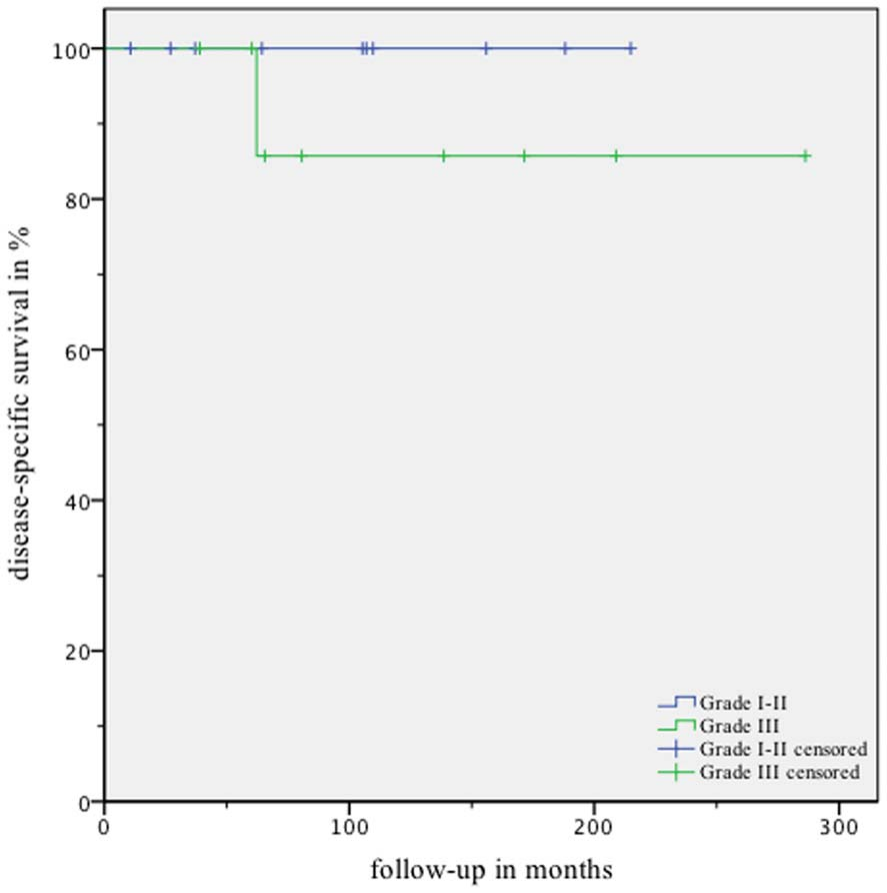
Disease-specific survival curves according to Kaplan-Meier for ependymoma WHO I and II (blue graph) and anaplastic ependymoma WHO III (green graph).

The actuarial 2-, 5- and 10-year overall- and disease-specific survival rates after IBT for patients with ependymoma WHO I and ependymoma WHO II were 90% and 100% each, respectively (Figures 3 and 4).

### Ependymoma WHO III (9 patients)

Median clinical follow up of this group was 80.6 months (range 34.6–286.2 months). Of the 9 patients, 2 patients (22.2%) were asymptomatic after interstitial brachytherapy, while 5 patients (55.6%) showed an improved or stable clinical status. One of the remaining 2 patients deteriorated clinically: A 29 year-old male who had been treated previously with surgery and external fractionated radiotherapy with a total dose of 36 Gy developed an impairment of his cognitive functions (Table 3). At that time, the tumor was stable. He died of pulmonary embolism after hip surgery 91.3 months after treatment. The second patient, a 24 year-old female, had also been treated previously with surgery, chemotherapy according to HIT 91 and external fractionated radiotherapy with a total dose of 30.5 Gy plus 10 Gy to a spinal metastasis at the level of S1. She developed a second spinal metastasis at the level of L2 58.7 months after interstitial brachytherapy resulting in complete paraparesis of the lower extremities. At this point the implanted tumor in the third ventricle showed a partial remission. This patient refused any further therapy and died 62.2 months after IBT treatment.

MR images showed a complete local remission in 11.1% (1/9), partial remission in 55.6% (5/9), and a stable disease in the remaining 33.3% (3/9) (Figure 2 and figures S1 and S2). No tumor progression was documented in this group. We did not observe any treatment-related mortality. The actuarial 2-, 5- and 10-year overall- and disease-specific survival rates after IBT were 100%, 100% and 86%, respectively (Figures 3 and 4).

## Discussion

The efficiency of stereotactically guided IBT for treating intracranial tumors not suited for microsurgery due to lack of accessibility has been convincingly demonstrated in current literature [13], [16], [18]. Nevertheless, reports on interstitial ependymoma irradiation with stereotactically implanted ^125^I seeds are rather scarce. Just a total of three cases have been published between 1993 and 2010 [18]–[20]. Hence, the data presented and analyzed here constitute the largest series obtained at a single institution that has been reported on and published so far.

One group [19] presented a case report of a patient with a large intraventricular low-grade ependymoma. The tumor was treated with stereotactically implanted ^125^I seeds for interstitial irradiation. With a tumor surface dose of 40 Gy the tumor shrank significantly within a few weeks. After a follow-up time of nearly 5 years the patient was still tumor free.

Chamberlain and coworkers [20] reported 16 patients treated with ^125^I seeds for interstitial brachytherapy and concomitant cisplatin (median tumor surface dose 50 Gy for 100 hours and cisplatin 20 mg/m^2^ per day for 5 days), including one patient with ependymoma. Korinthenberg and coworkers [21] reported on 94 patients with low-grade gliomas treated with temporarily implanted ^125^I seeds for interstitial brachytherapy also including only one patient with ependymoma; specific results concerning this particular ependymoma patient were not reported in both series.

Currently, in the literature, surgery is regarded as standard treatment for ependymoma. There are many retrospective studies claiming the extent of resection as a prognostic factor [22]–[26]. The 5-year survival rate of patients who underwent total resection or near total resection was 60% [26] and 80% [23] shrinking to a 5-year survival rate of 21% after only partial resection. However, ependymoma has a propensity to spread via the ventricular system, causing most recurrences. Clinically recognized leptomeningeal dissemination was reported to range from 0% to 22% [11], [27]. Therefore, external beam irradiation (EBI) is a further treatment option for ependymoma patients. The benefit of combined surgery and postoperative irradiation as compared to surgery alone has also been demonstrated in some more recent series, achieving 45% versus 0% 5-year event-free survival [25] and 51–70% versus 13% 5-year progression-free survival [23].

Combs et al. reported the results of 57 patients with circumscribed ependymomas all of whom underwent surgery after previously having been treated with radiotherapy. Overall 5-year survival rate was 80% in low-grade and 79% in high-grade tumors, respectively. Essentially, there was no difference in local failure rates between craniospinal irradiation and local radiotherapy. A rate of 83% for distant failure-free survival was observed in the group of patients with craniospinal irradiation as opposed to 93% in the group having received only local radiotherapy [28].

Regarding to chemotherapy, most retrospective studies evaluating adjuvant chemotherapy for pediatric ependymomas failed to detect favorable outcomes, except for one study which reported a 74% 5-year progression-free survival [29].

Due to the heterogeneous clinical behavior, the determination of valid biomarkers for the prediction of prognosis may be of great relevance. Costa et al. identified micro RNAs that were over- and under expressed in ependymomas compared to the normal ependymal tissue [12]. Referring to this, they uncovered associations between expression and overall- and progression-free survival.

In agreement with reports in the literature, we may conclude from our data that the extent (progression) of disease diagnosed on first admission of the patient appears to be an important indication of further prognosis in ependymoma cases. In our study, the patient suffering from ependymoma disseminated to the spine, although initially demonstrating local remission, subsequently presented with long-distance tumor dissemination.

We therefore believe that both examining the cerebrospinal fluid for malignant cells and providing MR-imaging for WHO II and III ependymomas are mandatory steps for initial staging.

Stereotactic interstitial brachytherapy may be considered superior to conventional radiation therapy inasmuch as precision of radiation dose distribution is concerned. Thus, by comparison, there frequently will be a reduction of radiation-induced damage imposed on normal brain tissue in the immediate vicinity of the irradiated tumor volume. This goes in parallel with an enhancement of the radiobiological effect on the tumor tissue itself. Due to a decreasing total brain dose, IBT may also have an important part in treating children with anaplastic ependymoma.

When comparing our results with IBT to those obtained using external radiation therapy, the following becomes obvious: A significantly higher rate of local tumor control (100%) in combination with a marked tumor remission (CR, PR) of 71.4% (15/21), as well as a generally prolonged survival time.

Regarding the risk of secondary malignancies after IBT of intracranial tumors, to our knowledge, there were no reports in the literature. Nevertheless, Moon and coworkers [30] reported on cancer incidences after localized therapy including radioactive implants. He stated that patients who received external fractionated irradiation had significantly higher odds of developing second cancers than patients who received radioactive implants.

This study demonstrates the safety as well as the efficiency of stereotactically guided interstitial ^125^I brachytherapy when treating patients suffering from intracranial ependymoma of all WHO grades.

For the first group (ependymoma WHO I and II, n = 12), the total actuarial survival rates for 2, 5- and 10 years were 90% at any time, respectively. The median follow-up period being >9 years, we observed either complete remission of the tumor (CR), partial remission (PR) or stable disease in all patients. This translates into the rate of local tumor control being 100%.

Following treatment, three patients had no symptoms left, while the remaining nine patients exhibited either an improvement of their condition or demonstrated a stable clinical status.

In addition, patients in the second group (anaplastic ependymoma WHO III, n = 9) also responded to stereotactic interstitial brachytherapy. This was reflected in high overall actuarial 2-, 5- and 10-year rates of survival (i. e. 100%, 100% and 70%, respectively).

Also in this group – with a median follow-up period of 7 years, we achieved a high local tumor control rate of 100% (1 CR, 5 PR and 3 SD). After interstitial brachytherapy, two patients (22.2%) were devoid of any symptoms. The remaining 5 patients were either improved or had attained a stable clinical status.

One patient who underwent multimodal treatment, including surgery and radiotherapy, deteriorated clinically. A further patient developed spinal metastasis 58.7 months after IBT. In this patient, neither surgery- nor IBT-related morbidity occurred. Remarkably, treatment-related mortality was absent in the patients of both groups. In two patients, acute morbidity occurred one and three days after interstitial brachytherapy, respectively. However, all symptoms vanished completely within one week following steroid medication. Two patients developed late morbidity after treatment. The first one exhibited left-sided facial nerve palsy grade III according to House-Brackmann three months after IBT. In the second patient, who had previously been treated surgically and with fractionated external radiation therapy at a total dose of 36 Gy, cognitive functions were impaired. It is likely that these deficits were caused by both external fractionated radiation therapies prior to IBT and by our treatment itself.

## Conclusions

Stereotactic interstitial brachytherapy for intracranial ependymoma has obvious virtues as a consequence of its minimal invasiveness, its inherent capability to achieve high rates of local tumor control, and its low incidence of treatment-related morbidity. Taken together, based on the limited size of our series, at present we should only like to indicate that stereotactically guided IBT may evolve into an attractive alternative to microsurgery as far as treatment of patients with ependymoma is concerned, either as a primary therapy for small, microsurgically inaccessible ependymomas, or as an adjuvant measure following microsurgical decompression of grossly symptomatic tumors. Additionally, IBT may be utilized as a salvage therapy with recurrently progressive ependymomas.

## Supporting Information

Figure S1 **Follow-up MRI of a 17-year old female with an ependymoma II adjacent to the fourth ventricle prior to IBT (upper line) and complete tumor remission 9 years after treatment (bottom line).**
(TIF)

Figure S2 **Follow-up MRI of a 36-year old male with an ependymoma III in the left ventricle prior to IBT (upper line) and complete tumor remission 5 years after treatment (bottom line).**
(TIF)

File S1 **Statement of the Ethic Committee German.**
(PDF)

File S2 **German Data Protection Legislation English Version.**
(PDF)

File S3 **Data Protection Legislation.**
(PDF)
