# Supplementary figures and images for: Intracranial Ependymoma: Long-Term Results in a Series of 21 Patients Treated with Stereotactic 125Iodine Brachytherapy
Source: PLoS One. 2012 Nov 5;7(11):e47266. doi: 10.1371/journal.pone.0047266 (PMC3489891; doi:10.1371/journal.pone.0047266)

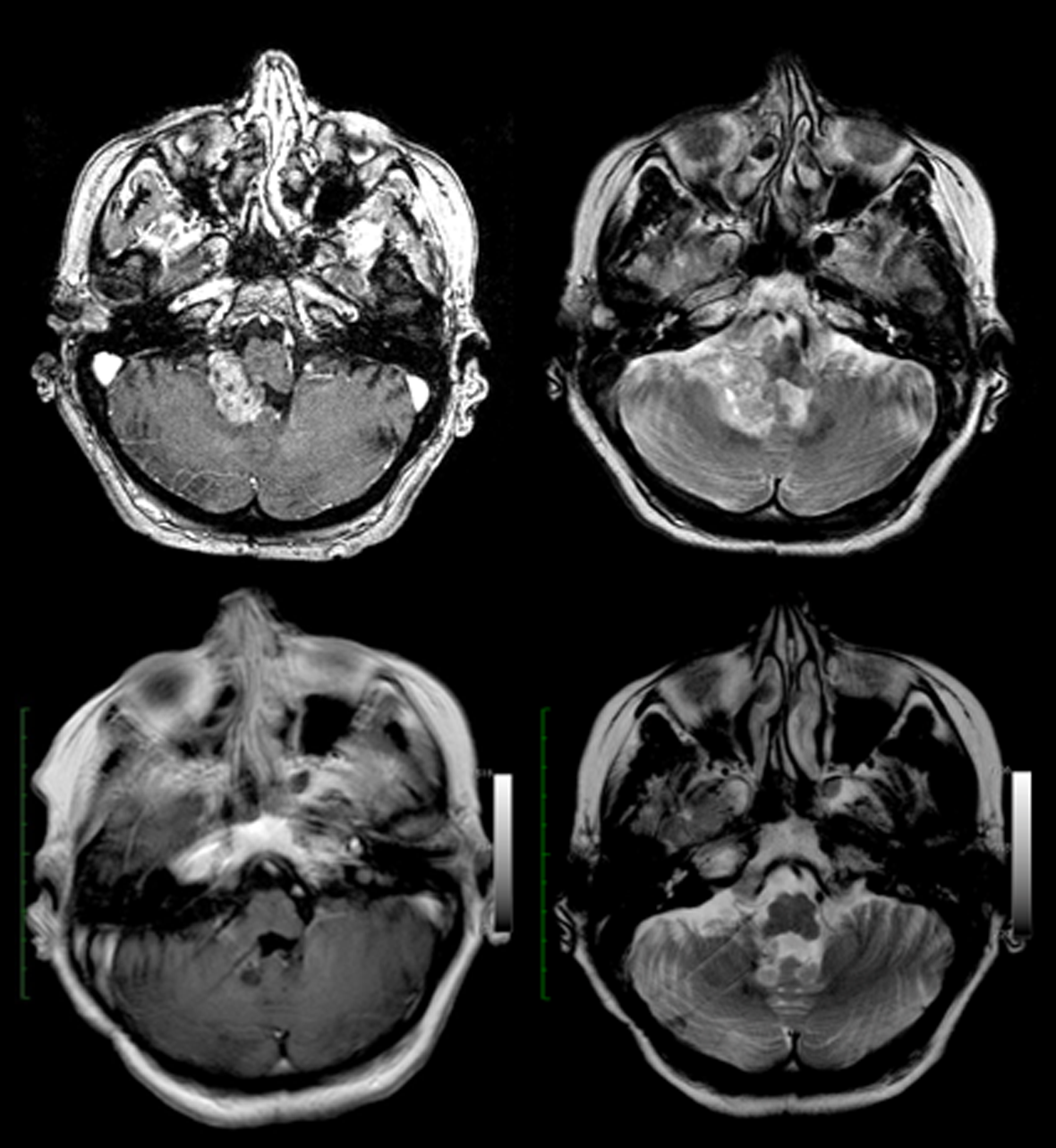

Supplement: Figure S1 — Follow-up MRI of a 17-year old female with an ependymoma II adjacent to the fourth ventricle prior to IBT (upper line) and complete tumor remission 9 years after treatment (bottom line). (TIF) [file pone.0047266.s001.tif]

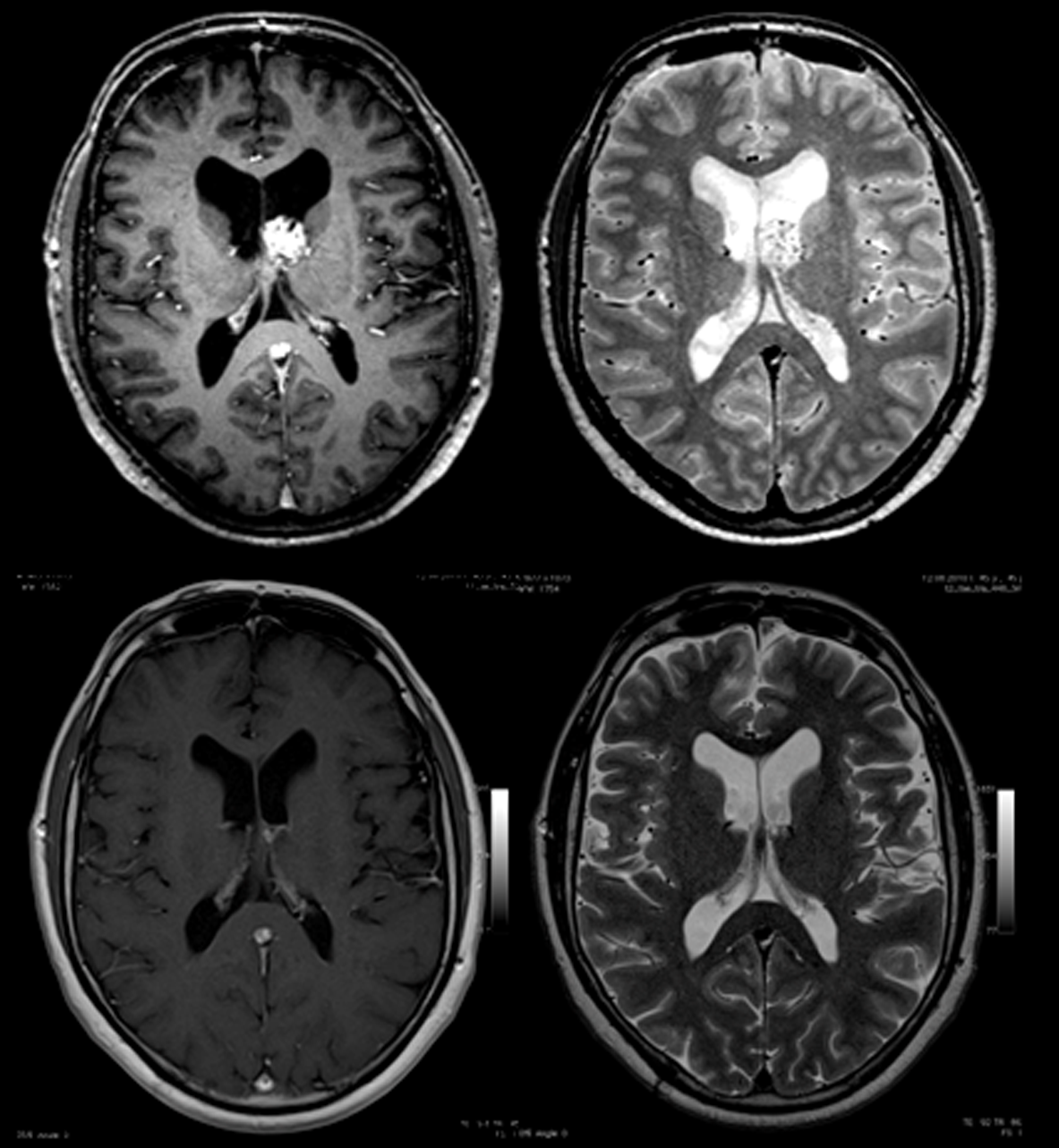

Supplement: Figure S2 — Follow-up MRI of a 36-year old male with an ependymoma III in the left ventricle prior to IBT (upper line) and complete tumor remission 5 years after treatment (bottom line). (TIF) [file pone.0047266.s002.tif]
