# Supplementary material for: Intracranial Ependymoma: Long-Term Results in a Series of 21 Patients Treated with Stereotactic 125Iodine Brachytherapy
Source: PLoS One. 2012 Nov 5;7(11):e47266. doi: 10.1371/journal.pone.0047266 (PMC3489891; doi:10.1371/journal.pone.0047266)
Supplement: File S1 — Statement of the Ethic Committee German. (PDF) [file pone.0047266.s003.pdf]

Universität zu Köln

Medizinische Fakultät der Universität zu Köln

Geschäftsstelle der Ethikkommission

Vorsitzender
Univ.-Prof. Dr. Walter Lehmacher

Leiter der Geschäftsstelle
Dr. med. Guido Grass
Telefon +49 221 478 87916

Wiss. Mitarbeiterinnen

Dipl.-Ges.-Ök. Karolina Mäder
Telefon +49 221 478 88844

Dipl.-Ges.-Ök. Christine Grimm
Telefon +49 221 478 97773

Dipl.-Ges.-Ök. Sabine Liese
Telefon +49 221 478 97772

Christin Willgrod M.A.
Telefon +49 221 478 97772

Sekretariat

Barbara Ulhardt M.A.
Telefon +49 221 478 4262
Telefax +49 221 478 6751

ek-med@uni-koeln.de
www.ek-koeln.de

To whom it may concern

The Ethics Committee of the Medical Faculty of the University of Cologne (Ethikkommission der Medizinischen Fakultät der Universität zu Köln) is the competent ethics committee for all clinical research involving human subjects performed at the University of Cologne and /or for all scientists and physicians of the University of Cologne including the University Hospital initiating or taking part in a clinical research project.

This is to state that under German Law no separate ethics application to and statement of ethical approval by the respective Local Institutional Board (in this case the Ethics Committee of the Medical Faculty of the University of Cologne) are required for performing purely retrospective clinical studies, i. e. studies extracting data from patient files, even if they are part of the individual case documentation.

Herewith we wish to point out that the authors of such studies are exempt from applying for ethical approval.

Köln, 29.03.2012

Prof. Dr. W. Lehmacher

Dr. med. G. Grass

Servicezeiten:
Mo. – Do. 9.00 – 16.00 Uhr
Fr. 9.00 – 12.00 Uhr
und nach Vereinbarung

Besucheradresse:
Gleueler Straße 70
Gebäude 5 / Eingang A
Etage 2a / Raum 015
50931 Köln (Lindenthal)

Postanschrift:
Gebäude 5, Kerpener Str. 62
50937 Köln

Bankverbindung:
Bank für Sozialwirtschaft Köln
BLZ 370 205 00
Kto.-Nr. 8 150 000
BIC BFSWDE31
