# Supplementary material for: Intracranial Ependymoma: Long-Term Results in a Series of 21 Patients Treated with Stereotactic 125Iodine Brachytherapy
Source: PLoS One. 2012 Nov 5;7(11):e47266. doi: 10.1371/journal.pone.0047266 (PMC3489891; doi:10.1371/journal.pone.0047266)
Supplement: File S2 — German Data Protection Legislation English Version. (PDF) [file pone.0047266.s004.pdf]

Federal Data Protection Act (BDSG)

In the version promulgated on 14 January 2003 (Federal Law Gazette I, p. 66),

last amended by Article 1 of the Act of 14 August 2009 (Federal Law Gazette I, p. 2814),

in force from 1 September 2009

The changes effective 1 April 2010 and 11 June 2010 are printed in italics. See footnote for date effective.

Table of Contents

Part I

General and common provisions

Section 1 Purpose and scope

Section 2 Public and private bodies

Section 3 Further definitions

Section 3a Data reduction and data economy

Section 4 Lawfulness of data collection, processing and use

Section 4a Consent

Section 4b Transfer of personal data abroad and to supranational or intergovernmental bodies

Section 4c Derogations

Section 4d Obligation to notify

Section 4e Contents of notification

Section 4f Data protection official

Section 4g Duties of the data protection official

Section 5 Confidentiality

Section 6 Inalienable rights of the data subject

Section 6 Rights of the data subject

Section 6a Automated individual decisions

Section 6b Monitoring of publicly accessible areas with optic-electronic devices

Section 6c Mobile storage and processing media for personal data

Section 7 Compensation

Section 8 Compensation in case of automated data processing by public bodies

Section 9 Technical and organizational measures

Section 9a Data protection audit

Section 10 Automated retrieval procedures

Section 11 Collection, processing or use of personal data on behalf of others

Part II

Data processing by public bodies

Chapter 1

Legal basis for data processing

Section 12 Scope

Section 13 Data collection

Section 14 Recording, alteration and use of data

Section 15 Transfer of data to public bodies

Section 16 Transfer of data to private bodies

Section 17 (deleted)

Section 18 Implementation of data protection in the federal administration

Chapter 2

Rights of the data subject

Section 19 Access to data

Section 19a Notification

Section 20 Rectification, erasure and blocking of data; right to object

Section 21 Appeals to the Federal Commissioner for Data Protection and Freedom of Information

Chapter 3

Federal Commissioner for Data Protection and Freedom of Information

Section 22 Election of the Federal Commissioner for Data Protection and Freedom of Information

Section 23 Legal status of the Federal Commissioner for Data Protection and Freedom of Information

Section 24 Monitoring by the Federal Commissioner for Data Protection and Freedom of Information

Section 25 Complaints lodged by the Federal Commissioner for Data Protection and Freedom of Information

Section 26 Additional duties of the Federal Commissioner for Data Protection and Freedom of Information

Part III

Data processing by private bodies and commercial enterprises under public law

Chapter 1

Legal basis for data processing

Section 27 Scope

Section 28 Collection and recording of data for own commercial purposes

Section 28a Data transfer to rating agencies

Section 28b Scoring

Section 29 Commercial data collection and recording for the purpose of transfer

Section 30 Commercial data collection and recording for the purpose of transfer in anonymous form

Section 30a Commercial data collection and recording for purposes of market or opinion research

Section 31 Special restrictions on use

Section 32 Data collection, processing and use for employment-related purposes

Chapter 2

Rights of the data subject

Section 33 Notification of the data subject

Section 34 Access to data

Section 34 Access to data

Section 35 Correction, deletion and blocking of data

Chapter 3

Supervisory authority

Section 36 (deleted)

Section 37 (deleted)

Section 38 Supervisory authority

Section 38a Codes of conduct to facilitate the application of data protection provisions

Part IV

Special provisions

Section 39 Restrictions on use of personal data subject to professional or special official secrecy

Section 40 Processing and use of personal data by research institutions

Section 41 Collection, processing and use of personal data by the media

Section 42 Data protection official of *Deutsche Welle*

Section 42a Obligation to notify in case of unlawful access to data

Part V

Final provisions

Section 43 Administrative offences

Section 44 Criminal offences

Part VI

Transitional provisions

Section 45 Current applications

Section 46 Continued validity of definitions

Section 47 Transitional provision

Section 48 Report of the Federal Government

Annex (to Section 9, first sentence)

Part I

General and common provisions

Section 1 Purpose and scope

(1) The purpose of this Act is to protect individuals against infringement of their right to privacy as the result of the handling of their personal data.

(2) This Act shall apply to the collection, processing and use of personal data by

1. public bodies of the Federation,
2. public bodies of the *Länder*, where data protection is not covered by *Land* legislation and where the *Länder*

a) execute federal law, or

b) act as judiciary bodies and administrative matters are not involved,

3. private bodies

that collect data for use in data processing systems, or use such systems to process or use data, or collect data in or from non-automated filing systems, or use such systems to process or use data, unless the data are collected, processed or used solely for personal or domestic activities.

(3) Where other federal laws apply to personal data and their publication, they shall take precedence over the provisions of this Act. The obligation to abide by legal obligations of secrecy or professional or special official secrecy not based on law shall remain unaffected.

(4) The provisions of this Act shall take precedence over those of the Administrative Procedures Act where personal data are processed in ascertaining the facts.

(5) This Act shall not apply in so far as a controller located in another European Union Member State or another state party to the Agreement on the European Economic Area collects, processes or uses personal data inside the country, except where such collection, processing or use is carried out by a branch inside the country. This Act shall apply in so far as a controller not located in a European Union Member State or other state party to the Agreement on the European Economic Area collects, processes or uses personal data inside the country. In so far as the controller is to be named under this Act, information on representatives located inside the country shall also be furnished. Sentences 2 and 3 shall not apply where data storage media are used solely for the purpose of transit through the country. Section 38 (1) first sentence shall remain unaffected.

Section 2 Public and private bodies

(1) “Public bodies of the Federation” shall mean the authorities, judiciary bodies and other public-law institutions of the Federation, of the direct federal corporations, institutions and foundations under public law as well as their associations irrespective of their legal forms. The successor companies created by law from the Special Fund “Deutsche Bundespost” shall be considered public bodies as long as they have an exclusive right under the Postal Act.

(2) “Public bodies of the *Länder*” shall mean the authorities, judiciary bodies and other public-law institutions of a *Land*, of a municipality, an association of

municipalities or other legal persons under public law subject to *Land* supervision as well as their associations irrespective of their legal forms.

(3) Private-law associations of public bodies of the Federation and the *Länder* performing public administration tasks shall be considered public bodies of the Federation irrespective of private shareholdings if

1. they operate in more than one *Land*, or
2. the Federation possesses the absolute majority of shares or votes.

Otherwise, they shall be regarded as public bodies of the *Länder*.

(4) “Private bodies” shall mean natural or legal persons, companies and other private-law associations not covered by subsections 1 through 3. If a private body performs sovereign public administration tasks, it shall be a public body within the meaning of this Act.

Section 3 Further definitions

(1) “Personal data” shall mean any information concerning the personal or material circumstances of an identified or identifiable natural person (“data subject”).

(2) “Automated processing” shall mean the collection, processing or use of personal data by means of data processing systems. A “non-automated filing system” shall mean any non-automated collection of personal data which is similarly structured and which can be accessed and evaluated according to specific characteristics.

(3) “Collection” shall mean the acquisition of data on the data subject.

(4) “Processing” shall mean the recording, alteration, transfer, blocking and erasure of personal data. Specifically, irrespective of the procedures applied,

1. “recording” shall mean the entry, recording or preservation of personal data on a storage medium so that they can be further processed or used,
2. “alteration” shall mean the modification of the substance of recorded personal data,
3. “transfer” shall mean the disclosure of personal data recorded or obtained by data processing to a third party either
   a) through transfer of the data to a third party, or
   b) by the third party inspecting or retrieving data available for inspection or retrieval,
4. “blocking” shall mean the identification of recorded personal data so as to restrict their further processing or use,
5. “erasure” shall mean the deletion of recorded personal data.

(5) “Use” shall mean any utilization of personal data other than processing.

(6) “Rendering anonymous” shall mean the alteration of personal data so that information concerning personal or material circumstances cannot be attributed to an identified or identifiable natural person or that such attribution would require a disproportionate amount of time, expense and effort.

(6a) “Aliasing” shall mean replacing the data subject’s name and other identifying features with another identifier in order to make it impossible or extremely difficult to identify the data subject.

(7) “Controller” shall mean any person or body which collects, processes or uses personal data on his, her or its own behalf, or which commissions others to do the same.

(8) ‘Recipient’ shall mean any person or body to whom or which data are disclosed. ‘Third party’ shall mean any person or body other than the controller. Third parties shall not mean the data subject or persons or bodies collecting, processing or using personal data in Germany, in another European Union Member State or another state party to the Agreement on the European Economic Area.

(9) ‘Special categories of personal data’ shall mean information on racial or ethnic origin, political opinions, religious or philosophical beliefs, trade-union membership, health or sex life.

(10) ‘Mobile personal storage and processing media’ shall mean data storage media

1. which are issued to the data subject,
2. on which personal data can be processed automatically, in addition to the storage function, by the issuing body or another body, and
3. which the data subject must use to influence such processing.

(11) ‘Employees’ shall mean

1. employees,
2. persons employed for the purpose of occupational training,
3. persons participating in measures to integrate them into the labour market or to clarify their ability or suitability for work (rehabilitation measures),
4. persons employed at certified workshops for persons with a disability,
5. persons employed under the Youth Volunteer Service Act,
6. persons comparable to employees due to their economic dependence, including home-based workers and those of similar status,
7. applicants for employment and those whose employment has ended,
8. civil servants, federal judges, military personnel and persons in the alternative civilian service.

Section 3a Data reduction and data economy

Personal data shall be collected, processed and used, and data processing systems shall be chosen and organized in accordance with the aim of collecting, processing and using as little personal data as possible. In particular, personal data shall be rendered anonymous or aliased as allowed by the purpose for which they are collected and/or further processed, and as far as the effort required is not disproportionate to the desired purpose of protection.

Section 4 Lawfulness of data collection, processing and use

(1) The collection, processing and use of personal data shall be lawful only if permitted or ordered by this Act or other law, or if the data subject has provided consent.

(2) Personal data shall be collected from the data subject. They may be collected without the data subject's participation only if

1. allowed or required by law, or
2. a) the data must be collected from other persons or bodies due to the nature of the administrative task to be performed or the commercial purpose, or

   b) collecting the data from the data subject would require disproportionate effort

and there are no indications that overriding legitimate interests of the data subject would be adversely affected.

(3) If personal data are collected from the data subject, the controller shall inform him/her as to

1. the identity of the controller,
2. the purposes of collection, processing or use, and
3. the categories of recipients only where, given the circumstances of the individual case, the data subject need not expect that his/her data will be transferred to such recipients,

unless the data subject is already aware of this information. If personal data are collected from the data subject pursuant to a law requiring the provision of such information, or if providing this information is required for the granting of legal benefits, the data subject shall be informed that providing this information is required or voluntary, as the case may be. The law and the consequences of refusing to provide information shall be explained to the data subject as necessary in the individual case.

Section 4a Consent

(1) Consent shall be effective only when based on the data subject's free decision. Data subjects shall be informed of the purpose of collection, processing or use and, as necessary in the individual case or on request, of the consequences of withholding consent. Consent shall be given in writing unless special circumstances warrant any other form. If consent is to be given together with other written declarations, it shall be made distinguishable in its appearance.

(2) In the field of scientific research, a special circumstance as referred to in subsection 1 third sentence shall be deemed to exist if the defined purpose of research would be seriously affected if consent were obtained in writing. In this case, the information referred to in subsection 1 second sentence and the reasons the defined purpose of research would be seriously affected shall be recorded in writing.

(3) Where special categories of personal data (Section 3 (9)) are collected, processed or used, the consent must also refer specifically to these data.

Section 4b Transfer of personal data abroad and to supranational or intergovernmental bodies

(1) The transfer of personal data to bodies

1. in other European Union Member States,
2. in other states parties to the Agreement on the European Economic Area, or
3. of the institutions of the European Communities

shall be subject to Section 15 (1), Section 16 (1) and Sections 28 through 30a in accordance with the laws and agreements applicable to such transfers, if data are transferred in connection with activities which fall fully or partly within the scope of the law of the European Communities.

(2) Subsection 1 shall apply accordingly to transfers of personal data to bodies under subsection 1, if data are transferred in connection with activities which do not fall within the scope of the law of the European Communities, and to transfers of personal data to other bodies abroad or to supranational or intergovernmental bodies. Personal data shall not be transferred if the data subject has a legitimate interest in ruling out the possibility of transfer, especially if the bodies listed in the first sentence fail to ensure an adequate level of data protection. The second sentence shall not apply if transfer is necessary for a public body of the Federation to carry out its duties for compelling reasons of defence or to fulfil supranational or intergovernmental obligations in the field of crisis management or conflict prevention or for humanitarian measures.

(3) The adequacy of the level of protection afforded shall be assessed in the light of all the circumstances surrounding a data transfer operation or set of data transfer operations; particular consideration shall be given to the nature of the data, the purpose and duration of the proposed processing, the country of origin and country of final destination, the rules of law, both general and sectoral, applicable to the recipient and the professional rules and security measures applicable to the recipient.

(4) In the cases referred to in Section 16 (1) no. 2, the body transferring the data shall inform the data subject of the data transfer. This shall not apply if it can be assumed that the data subject will otherwise acquire this information, or if informing the data subject would endanger public safety or otherwise be detrimental to the Federation or a *Land*.

(5) The body transferring the data shall be responsible for ensuring the lawfulness of the transfer.

(6) The body to which the data are transferred shall be informed of the purpose for which the data are being transferred.

Section 4c Derogations

(1) In connection with activities which fall fully or partly within the scope of the law of the European Communities, the transfer of personal data to bodies other than those listed in Section 4b (1) shall be lawful, even if they do not ensure an adequate level of data protection, if

1. the data subject has given his/her consent,

2. the transfer is necessary for the performance of a contract between the data subject and the controller or the implementation of precontractual measures taken in response to the data subject's request,
3. the transfer is necessary for the conclusion or performance of a contract which has been or is to be concluded in the interest of the data subject between the controller and a third party,
4. the transfer is necessary or legally required on important public interest grounds, or for the establishment, exercise or defence of legal claims,
5. the transfer is necessary in order to protect the vital interests of the data subject, or
6. the transfer is made from a register which is intended to provide information to the public and which is open to consultation either by the public in general or by any person who can demonstrate legitimate interest, to the extent that the conditions laid down in law are fulfilled in the particular case.

The body to which the data are transferred shall be informed that the transferred data may be processed or used only for the purpose for which they are being transferred.

(2) Without prejudice to subsection 1 first sentence, the competent supervisory authority may authorize a transfer or a set of transfers of personal data to bodies other than those listed in Section 4b (1), where the controller adduces adequate safeguards with respect to the protection of privacy and exercise of the corresponding rights; such safeguards may in particular result from contractual clauses or binding corporate regulations. The Federal Commissioner for Data Protection and Freedom of Information shall be responsible in the case of postal and telecommunications companies. Where public bodies are to transfer personal data, they shall undertake the examination referred to in the first sentence.

(3) The *Länder* shall notify the Federation of decisions made in accordance with subsection 2 first sentence.

Section 4d Obligation to notify

(1) Before carrying out any automated processing operations, private controllers shall notify the competent supervisory authority, while federal controllers and controllers of postal and telecommunications companies shall notify the Federal Commissioner for Data Protection and Freedom of Information in accordance with Section 4e.

(2) The obligation to notify shall not apply if the controller has appointed a data protection official.

(3) Further, the obligation to notify shall not apply if the controller collects, processes or uses personal data for its own persons and no more than nine employees are employed in collecting, processing or using personal data, and either the data subject has given his/her consent or the collection, processing or use is needed to create, carry out or terminate a legal obligation or a quasi-legal obligation with the data subject.

(3) Further, the obligation to notify shall not apply if the controller collects, processes or uses personal data for its own persons and, as a rule, no more than nine employees are permanently employed in collecting, processing or using personal data, and either the data subject has given his/her consent or the collection,

processing or use is needed to create, carry out or terminate a legal obligation or a quasi-legal obligation with the data subject.[1]

(4) Subsections 2 and 3 shall not apply in case of automated processing in which the controller commercially records personal data

1. for the purpose of transfer,
2. for the purpose of transfer in anonymous form, or
3. the purposes of market or opinion research.

(5) Where automated processing operations present special risks to the rights and freedoms of data subjects, these operations shall be examined before the start of processing (prior checking). Such prior checks shall be carried out in particular

1. if special categories of personal data (Section 3 (9)) are to be processed, or
2. the processing of personal data is intended to assess the data subject's personality and his/her abilities, performance or behaviour,

unless a statutory obligation applies, the data subject's consent has been given, or the collection, processing or use is needed to create, carry out or terminate a legal obligation or quasi-legal obligation with the data subject.

(6) The data protection official shall be responsible for conducting prior checks. The data protection official shall carry out prior checks following receipt of the overview in accordance with Section 4g (2) first sentence. In case of doubt, the data protection official shall consult the supervisory authority or, in case of postal and telecommunications companies, the Federal Commissioner for Data Protection and Freedom of Information.

Section 4e Contents of notification

Where automated processing operations are subject to the obligation to notify, they shall include the following information:

1. name or company of the controller,
2. owners, management boards, managing directors or other managers appointed in accordance with the law or company regulations, and the persons in charge of data processing,
3. the controller's address,
4. the purposes of the data collection, processing or use,
5. a description of the category or categories of data subject and of the data or categories of data relating to them,
6. the recipients or categories of recipient to whom the data might be disclosed,
7. standard data retention periods,
8. plans to transfer data to third countries,
9. a general description allowing a preliminary assessment to be made of the appropriateness of the measures taken pursuant to Section 9 to ensure security of processing.

---

[1] In force from 1 April 2010.

Section 4d (1) and (4) shall apply accordingly to the amendment of information provided under the first sentence and to the start and conclusion of the activity subject to the obligation to notify.

Section 4f Data protection official

(1) Public and private bodies which process personal data by automated means shall appoint in writing a data protection official. Private bodies shall be obligated to appoint a data protection official within one month of commencing their activities. The same shall apply where personal data are collected, processed or used by other means and, as a rule, at least 20 persons are employed for this purpose. The first and second sentences shall not apply to private bodies in which, as a rule, no more than nine persons are permanently employed in the automated processing of personal data. One data protection official may be appointed for several areas where the structure of a public body so requires. Where private bodies carry out automated processing subject to prior checking, or commercially carry out automated processing of personal data for the purpose of transfer, transfer in anonymous form or for purposes of market or opinion research, they shall appoint a data protection official irrespective of the number of persons employed in automated processing.

(2) Only persons with the specialized knowledge and reliability necessary to carry out their duties may be appointed to serve as data protection officials. The necessary level of specialized knowledge is determined in particular by the extent of data processing carried out by the controller and the protection required by the personal data collected or used by the controller. A person from outside the controller may be appointed data protection official; monitoring shall also extend to personal data which are subject to professional or special official secrecy, in particular tax secrecy under Section 30 of the German Fiscal Code. With the consent of their supervisory authority, public bodies may appoint an employee from another public body as their data protection official.

(3) Data protection officials shall be directly subordinate to the head of the public or private body. They shall be free to use their specialized knowledge in the area of data protection. They may not be penalized for performing their duties. The appointment of a data protection official may be revoked by applying Section 626 of the Civil Code accordingly; in the case of private bodies, the appointment may be revoked also at the request of the supervisory authority. If a data protection official is to be appointed under subsection 1, then this employment may not be terminated, unless the controller has just cause to terminate without notice. After the data protection official has been recalled, he or she may not be terminated for a year following the end of appointment, unless the controller has just cause to terminate without notice. The controller shall enable the data protection official to take part in advanced training measures and shall assume the expense of such measures in order for the data protection official to maintain the specialized knowledge to perform his or her duties.

(4) Data protection officials shall be obligated to secrecy concerning the identity of data subjects and concerning circumstances enabling data subjects to be identified, unless they are released from this obligation by the data subject.

(4a) Where in the course of their activities data protection officials become aware of data for which the head of a public or private body or a person employed by such a body has the right to refuse to give evidence, this right shall also apply to data protection officials and their assistants. The person to whom the right to refuse to give evidence applies for professional reasons shall decide whether to exercise this right unless it is impossible to effect such a decision in the foreseeable future. Where the right of data protection officials to refuse to give evidence applies, their files and other documents shall not be subject to seizure.

(5) The public and private bodies shall support data protection officials in performing their duties and shall provide assistants, premises, furnishings, equipment and other resources as needed to perform these duties. Data subjects may contact the data protection official at any time.

Section 4g Duties of the data protection official

(1) The data protection official shall work to ensure compliance with this Act and with other data protection provisions. For this purpose, in case of doubt the data protection official may consult the competent authority responsible for monitoring the controller's data protection. The data protection official may make use of advising as referred to in Section 38 (1) second sentence. In particular, the data protection official shall

1. monitor the proper use of data processing programs used to process personal data; for this purpose, the data protection official shall be informed in good time of projects for the automated processing of personal data,
2. take appropriate measures to familiarize persons employed in the processing of personal data with the provisions of this Act and other data protection provisions, and with the various special requirements of data protection.

(2) The controller shall provide the data protection official with an overview of the information listed in Section 4e, first sentence, and a list of persons entitled to access. The data protection official shall make the information referred to in Section 4e first sentence nos. 1 through 8 available in an appropriate form to any person on request.

(2a) Where no obligation to appoint a data protection official applies at a private body, the head of the private body shall ensure that the duties referred to in subsections 1 and 2 are performed by other means.

(3) Subsection 2 second sentence shall not apply to the authorities referred to in Section 6 (2) fourth sentence. Subsection 1 second sentence shall apply on the condition that the authority's data protection official contacts the head of the authority; any disagreements between the authority's data protection official and the head of the authority shall be settled by the supreme federal authority.

Section 5 Confidentiality

Persons employed in data processing shall not collect, process or use personal data without authorization (confidentiality). Such persons, when employed by private bodies, shall be obligated when taking up their duties to maintain confidentiality. The obligation of confidentiality shall continue after their employment ends.

Section 6 Inalienable rights of the data subject

Section 6 Rights of the data subject[2]

(1) The data subject's right of access (Sections 19, 34) and to rectification, erasure or blocking (Sections 20, 35) may not be excluded or restricted by a legal transaction.

(2) If the data subject's data are recorded automatically in such a way that more than one body is authorized to record the data, and the data subject is unable to determine which body recorded the data, the data subject may contact each of these bodies. Each body shall be obligated to forward the data subject's request to the body which recorded the data. The data subject shall be informed that the request has been forwarded and to which body it was forwarded. If they record personal data in performing their legally mandated duties within the scope of the German Fiscal Code for purposes of monitoring and examination, the bodies listed in Section 19 (3), the public prosecution authorities, the police and the public finance authorities may inform the Federal Commissioner for Data Protection and Freedom of Information instead of the data subject. In this case, the further procedure shall be based on Section 19 (6).

(3) Personal data concerning the data subject's exercise of a right based on this or other data protection provisions may be used only to fulfil obligations of the controller arising from the exercise of this right.[3]

Section 6a Automated individual decisions

(1) Decisions which produce legal effects concerning the data subject or significantly affect him/her may not be based solely on automated processing of personal data intended to evaluate certain personal aspects relating to him/her.

In particular, a decision not made by a natural person based on the evaluation of content shall constitute a decision based solely on automated processing.[4]

(2) This shall not apply if

1. the decision is taken in the course of entering into or performing a contractual relationship or other legal relationship, and the request lodged by the data subject has been satisfied, or
2. there are suitable measures to safeguard the data subject's legitimate interests and the controller informs the data subject that a decision within the meaning of subsection 1 has been taken. In particular, arrangements allowing data subjects to assert their point of view shall constitute suitable measures. The controller shall be obligated to review its decision.
2. *there are suitable measures to safeguard the data subject's legitimate interests, the controller informs the data subject that a decision within the*

---

[2] In force from 1 April 2010.

[3] In force from 1 April 2010.

[4] In force from 1 April 2010.

meaning of subsection 1 has been taken, and upon request explains the main reasons for this decision.[5]

(3) The data subject's right of access under Sections 19 and 34 shall also extend to the logic involved in the automated processing of his or her personal data.

Section 6b Monitoring of publicly accessible areas with optic-electronic devices

(1) Monitoring publicly accessible areas using optic-electronic devices (video surveillance) shall be lawful only as far as necessary

1. for public bodies to perform their duties,
2. to exercise the right to determine who shall be allowed or denied access, or
3. to pursue legitimate interests for specifically defined purposes,

and there are no indications of overriding legitimate interests of the data subject.

(2) Suitable measures shall be taken to make clear that the area is being monitored and to identify the controller.

(3) Data collected under subsection 1 may be processed or used if necessary to achieve the intended purposes and if there are no indications of overriding legitimate interests of the data subject. These data may be processed or used for another purpose only if necessary to prevent threats to state and public security or to prosecute crimes.

(4) Where a specific person can be identified using data collected through video surveillance, the person shall be notified of processing or use in accordance with Sections 19a and 33.

(5) The data shall be erased as soon as they are no longer needed to achieve the purpose or if further storage would conflict with legitimate interests of the data subject.

Section 6c Mobile storage and processing media for personal data

(1) A body which issues mobile storage and processing media for personal data or which applies to such media a procedure for the automated processing of personal data which runs wholly or partly on such media, or which alters or makes available such a procedure shall

1. inform the data subject of its identity and address,
2. explain to the data subject, in generally understandable terms, how the medium works, including the type of personal data to be processed,
3. inform the data subject how to exercise his or her rights under Sections 19, 20, 34 and 35, and
4. inform the data subject what measures are to be taken in case the medium is lost or destroyed, if the data subject is not already aware of this.

(2) The body subject to the obligations in subsection 1 shall ensure that devices or facilities necessary for data subjects to exercise their right of access are available in sufficient quantity for use free of charge.

---

[5] In force from 1 April 2010.

(3) Communication operations which initiate data processing on the medium must be clearly apparent to the data subject.

Section 7 Compensation

If a controller harms a data subject through collection, processing or use of his or her personal data which is unlawful or improper under this Act or other data protection provisions, the controller or its supporting organization shall be obligated to compensate the data subject for damage suffered. The obligation to provide compensation shall be waived if the controller exercised due care in the case.

Section 8 Compensation in case of automated data processing by public bodies

(1) If a public body harms a data subject through collection, processing or use of his or her personal data which is unlawful or improper under this Act or other data protection provisions, the body’s supporting organization shall be obligated to compensate the data subject for damage suffered irrespective of any fault.

(2) In case of a serious violation of privacy, the data subject shall receive appropriate financial compensation for non-financial damage suffered.

(3) Claims under subsections 1 and 2 shall be limited to a total of € 130,000. If compensation exceeding the maximum of € 130,000 is to be paid to more than one person due to the same incident, the compensation paid to each person shall be reduced in proportion to the maximum amount.

(4) If, in the case of automated processing, more than one body is authorized to record data and the injured person is unable to determine which body recorded his/her data, then each body shall be liable.

(5) Section 254 of the Civil Code shall apply to contributory negligence on the part of the data subject.

(6) The limitation provisions stipulated for tortious acts in the Civil Code shall apply accordingly with regard to statutory limitation.

Section 9 Technical and organizational measures

Public and private bodies which collect, process or use personal data on their own behalf or on behalf of others shall take the necessary technical and organizational measures to ensure the implementation of the provisions of this Act, especially the requirements listed in the Annex to this Act. Measures shall be necessary only if the effort required is in reasonable proportion to the desired purpose of protection.

Section 9a Data protection audit

In order to improve data protection and data security, suppliers of data processing systems and programs, and bodies conducting data processing may have independent and approved experts examine and evaluate their data protection strategy and their technical facilities and may publish the results of this examination. The detailed requirements pertaining to examination and evaluation, the procedure and the selection and approval of experts shall be covered in a separate law.

Section 10 Automated retrieval procedures

(1) It shall be lawful to establish an automated procedure to retrieve personal data as long as this procedure is appropriate in view of the legitimate interests of data

subjects and the tasks or commercial purposes of the bodies involved. Provisions on the lawfulness of individual retrieval shall remain unaffected.

(2) The bodies involved shall ensure that the lawfulness of the retrieval procedure can be monitored. For this purpose, they shall specify in writing:

1. the reason for and purpose of the retrieval procedure,
2. third parties to which data are transferred,
3. the type of data to be transferred,
4. technical and organizational measures required under Section 9.

In the public sector, the supervisory authorities may specify this information as necessary.

(3) In cases where the bodies referred to in Section 12 (1) are involved, the Federal Commissioner for Data Protection and Freedom of Information shall be informed when retrieval procedures are established and of the information specified under subsection 2. Establishing retrieval procedures in which the bodies referred to in Section 6 (2) and Section 19 (3) are involved shall be lawful only if the federal or *Land* ministry responsible for the recording body and the retrieving body has given its consent.

(4) The lawfulness of individual retrieval shall be the responsibility of the third party to which data are transferred. The recording body shall examine the lawfulness of retrieval only if there is cause for such examination. The recording body shall ensure that the transfer of personal data can be ascertained and checked at least by means of suitable random sampling procedures. If an entire collection of personal data is retrieved or transferred (batch processing), it shall be sufficient to ensure that the lawfulness of retrieval or transfer of the entire collection can be ascertained and checked.

(5) Subsections 1 through 4 shall not apply to the retrieval of generally accessible data. Generally accessible data are those which anyone can use, with or without prior registration, permission or the payment of a fee.

Section 11 Collection, processing or use of personal data on behalf of others

(1) If other bodies collect, process or use personal data on behalf of the controller, the controller shall be responsible for compliance with the provisions of this Act and other data protection provisions. The rights referred to in Sections 6, 7 and 8 shall be asserted with regard to the controller.

(2) The processor shall be chosen carefully, with special attention to the suitability of the technical and organizational measures applied by the processor. The work to be carried out by the processor shall be specified in writing, including in particular the following:

1. the subject and duration of the work to be carried out,
2. the extent, type and purpose of the intended collection, processing or use of data, the type of data and category of data subjects,
3. the technical and organizational measures to be taken under Section 9,
4. the rectification, erasure and blocking of data,
5. the processor’s obligations under subsection 4, in particular monitoring,

6. any right to issue subcontracts,
7. the controller's rights to monitor and the processor's corresponding obligations to accept and cooperate,
8. violations by the processor or its employees of provisions to protect personal data or of the terms specified by the controller which are subject to the obligation to notify,
9. the extent of the controller's authority to issue instructions to the processor,
10. the return of data storage media and the erasure of data recorded by the processor after the work has been carried out.

In case of public bodies, the work to be carried out may also be specified by the authority responsible for expert supervision. The controller shall verify compliance with the technical and organizational measures taken by the processor before data processing begins and regularly thereafter. The result shall be documented.

(3) The processor may collect, process or use the data only as instructed by the controller. If the processor believes that an instruction by the controller violates this Act or other data protection provisions, the processor shall inform the controller of this immediately.

(4) For the processor, other than Sections 5, 9, 43 (1) no. 2, Sections 10 and 11 (2) nos. 1 through 3 and (3), and Section 44, only the provisions on data protection monitoring or supervision shall apply, namely for

1. a) public bodies,
   b) private bodies, when the majority of shares or votes is publicly owned and the controller is a public body,

   Sections 18, 24 through 26 or the corresponding provisions of the data protection laws of the *Länder*,
2. other private bodies, where they collect, process or use personal data on behalf of others for commercial purposes as service providers, Sections 4f, 4g and 38.

(5) Subsections 1 through 4 shall apply accordingly if other bodies carry out the inspection or maintenance of automated procedures or data processing systems and the possibility of access to personal data during such inspection and maintenance cannot be ruled out.

Part II

Data processing by public bodies

Chapter 1

Legal basis for data processing

Section 12 Scope

(1) The provisions of this Part shall apply to public bodies of the Federation where they are not engaged in competition as commercial enterprises under public law.

(2) Where data protection is not governed by *Land* law, Sections 12 through 16 and Sections 19 and 20 shall also apply to the public bodies of the *Länder*, where they

1. execute federal law and are not engaged in competition as commercial enterprises under public law, or
2. act as judiciary bodies and administrative matters are not involved.

(3) Section 23 (4) shall apply accordingly to data protection officials at *Land* level.

(4) If personal data are collected, processed or used for the purpose of past, current or future employment contracts, Section 28 (2) no. 2 and Sections 32 through 35 shall apply in the place of Sections 13 through 16 and Sections 19 and 20.

Section 13 Data collection

(1) Collecting personal data shall be lawful when the knowledge of such data is necessary for the controller to perform its tasks.

(1 a) If personal data are collected from a private body rather than from the data subject, this body shall be informed of the legal provision requiring the supply of information or that such supply is voluntary.

(2) Collecting special categories of personal data (Section 3 (9)) shall be lawful only where

1. allowed by law or urgently required for reasons of important public interest,
2. the data subject has given his consent in accordance with Section 4a (3),
3. necessary to protect the vital interests of the data subject or of another person where the data subject is physically or legally incapable of giving his consent,
4. data are involved which the data subject has manifestly made public,
5. necessary to prevent a significant threat to the public security,
6. urgently required to prevent significant disadvantages to the common good or to preserve significant concerns of the common good,
7. required for the purposes of preventive medicine, medical diagnosis, the provision of care or treatment or the management of health-care services, and where these data are processed by health professionals or other persons subject to the obligation of professional secrecy,
8. necessary for the purposes of scientific research, where the scientific interest in carrying out the research project significantly outweighs the data subject’s interest in ruling out the possibility of collection and the purpose of the research cannot be achieved in any other way or would require a disproportionate effort, or
9. required for compelling reasons of defence or to fulfil supranational or intergovernmental obligations of a public body of the Federation in the field of crisis management or conflict prevention or for humanitarian measures.

Section 14 Recording, alteration and use of data

(1) The recording, alteration or use of personal data shall be lawful when required to carry out the tasks for which the controller is responsible and for the purpose for

which the data were collected. If no prior collection took place, the data may be altered or used only for the purpose for which they were recorded.

(2) Recording, alteration or use for other purposes shall be lawful only if

1. allowed or required by law,
2. the data subject has given his consent,
3. this is clearly in the interest of the data subject and there is no reason to assume that the data subject would refuse to give his consent if he knew of such other purpose,
4. information supplied by the data subject must be checked because there is reason to believe this information is incorrect,
5. the data are generally accessible or the controller would be allowed to publish them, unless the data subject has a clear and overriding legitimate interest in ruling out the possibility of a change of purpose,
6. required to prevent significant disadvantages to the common good or a threat to public security or to preserve significant concerns of the common good,
7. required to prosecute criminal or administrative offences, to enforce sentences or measures within the meaning of Section 11 (1) no. 8 of the Criminal Code or of reformatory or disciplinary measures within the meaning of the Youth Courts Act or to enforce decisions on fines,
8. required to prevent a serious infringement of the rights of another person, or
9. necessary for the purposes of scientific research, where the scientific interest in carrying out the research project significantly outweighs the data subject's interest in ruling out the possibility of collection and the purpose of the research cannot be achieved in any other way or would require a disproportionate effort.

(3) Processing or use for the purpose of exercising supervisory and monitoring authority, of auditing or conducting organizational studies for the controller shall not constitute processing or use for other purposes. This shall also apply to processing or use for training or examination purposes by the controller, unless the data subject has overriding legitimate interests.

(4) Personal data recorded exclusively for purposes of monitoring data protection, safeguarding data or ensuring proper operation of a data processing system may only be used for these purposes.

(5) Recording, altering or using special categories of personal data (Section 3 (9)) for other purposes shall be lawful only if

1. the conditions are met which would allow collection under Section 13 (2) nos. 1 through 6 or 9, or
2. necessary for the purposes of scientific research, where the public interest in carrying out the research project significantly outweighs the data subject's interest in ruling out the possibility of collection and the purpose of the research cannot be achieved in any other way or would require a disproportionate effort.

In weighing the public interest under the first sentence, no. 2, special attention shall be paid to the scientific interest in the research project.

(6) Recording, altering or using special categories of personal data (Section 3 (9)) for the purposes referred to in Section 13 (2) no. 7 shall be subject to the obligation of secrecy which applies to the persons referred to in Section 13 (2) no. 7.

Section 15 Transfer of data to public bodies

(1) Transfer of personal data to public bodies shall be lawful if

1. required to carry out the tasks for which the body transferring the data or the third party to which the data are transferred is responsible, and
2. the conditions are met which would allow use under Section 14.

(2) The body transferring the data shall be responsible for ensuring the lawfulness of the transfer. If the data are transferred at the request of the third party to which the data are transferred, the third party shall be responsible. In this case, the body transferring the data shall examine only whether the request for transfer lies within the remit of the third party to which the data are transferred, unless there is special reason to examine the lawfulness of transfer. Section 10 (4) shall remain unaffected.

(3) The third party to which the data are transferred may process or use these data for the purpose for which they were transferred. Processing or use for other purposes shall be lawful only under the conditions of Section 14 (2).

(4) Subsections 1 through 3 shall apply accordingly to the transfer of personal data to religious associations under public law, if it is ensured that these religious associations take sufficient data protection measures.

(5) If personal data which may be transferred under subsection 1 are linked to other personal data of the data subject or a third party in such a way that they cannot be separated without a disproportionate effort, then it shall be lawful to transfer also these data, unless the data subject or a third party clearly has an overriding legitimate interest in keeping them secret; use of these data shall not be lawful.

(6) Subsection 5 shall apply accordingly if personal data are transferred within the same public body.

Section 16 Transfer of data to private bodies

(1) Transfer of personal data to private bodies shall be lawful if

1. required to carry out the tasks for which the transferring party is responsible and the conditions are met which would allow use under Section 14, or
2. the third party to which the data are transferred provides credible evidence of its legitimate interest in knowledge of the data to be transferred and the data subject has no legitimate interest in ruling out the possibility of transfer. By way of derogation from the first sentence no. 2, the transfer of special categories of personal data (Section 3 (9)) shall be lawful only if the conditions are met which would allow use under Section 14 (5) and (6) or as far as necessary for the establishment, exercise or defence of legal claims.

(2) The body transferring the data shall be responsible for ensuring the lawfulness of the transfer.

(3) In the cases referred to in subsection 1 no. 2, the body transferring the data shall inform the data subject of the transfer of his/her data. This shall not apply if it can be assumed that the data subject will otherwise acquire this information, or if informing the data subject would endanger public safety or otherwise be detrimental to the Federation or a *Land*.

(4) The third party to which the data are transferred may process or use these data only for the purpose for which they were transferred. The transferring body shall point this out to the third party. Processing or use for other purposes shall be lawful if transfer would be lawful under subsection 1 and the transferring body has consented.

Section 17 (deleted)

Section 18 Implementation of data protection in the federal administration

(1) The supreme federal authorities, the president of the Federal Railway Property Agency, and the direct federal corporations, institutions and foundations under public law subject only to legal supervision by the Federal Government or a supreme federal authority shall ensure that this Act and other data protection provisions are implemented within their area of responsibility. The same shall apply to the boards of directors of the successor companies created by law from the Special Fund "Deutsche Bundespost" as long as they have an exclusive right under the Postal Act.

(2) The public bodies shall keep a register of the data processing systems used. For their automated processing, they shall record in writing the information under Section 4e and the legal basis for processing. This requirement may be waived in the case of automated processing for administrative purposes which does not restrict the data subject's right of access under Section 19 (3) or (4). The written records may be combined for automated processing conducted more than once in the same or similar manner.

Chapter 2

Rights of the data subject

Section 19 Access to data

(1) Upon request, data subjects shall be given information on

1. recorded data relating to them, including information relating to the source of the data,
2. the recipients or categories of recipients to which the data are transferred, and
3. the purpose of recording the data.

The request should specify the type of personal data on which information is to be given. If the personal data are recorded neither in automated form nor in non-automated filing systems, this information shall be provided only if the data subject provides information enabling the data to be located and if the effort required is not disproportionate to the data subject's interest in the information. The controller shall exercise due discretion in determining the procedure for providing such information and in particular the form in which it is provided.

(2) Subsection 1 shall not apply to personal data recorded only because they may not be erased due to legal, statutory or contractual provisions on retention, or only for purposes of monitoring data protection or safeguarding data, and providing information would require a disproportionate effort.

(3) If the provision of information relates to the transfer of personal data to authorities for the protection of the constitution, to the Federal Intelligence Service, the Military Counterintelligence Service and, as far as the security of the Federation is concerned, other agencies of the Federal Ministry of Defence, such provision shall be lawful only with the consent of these bodies.

(4) Information shall not be provided if

1. the information would endanger the orderly performance of tasks for which the controller is responsible,
2. the information would threaten the public security or order or otherwise be detrimental to the Federation or a *Land*, or
3. the data or the fact of their recording, in particular due to the overriding legitimate interests of a third party, must be kept secret by law or due to the nature of the data,

and therefore the data subject's interest in obtaining information shall not take precedence.

(5) No reasons must be given for refusing to provide information if stating the actual and legal grounds for refusal would threaten the purpose of refusing to provide information. In this case, data subjects shall be informed of the possibility to contact the Federal Commissioner for Data Protection and Freedom of Information.

(6) If no information is provided to the data subject, at the data subject's request this information shall be supplied to the Federal Commissioner for Data Protection and Freedom of Information unless the relevant supreme federal authority finds in the individual case that doing so would endanger the security of the Federation or a *Land*. The information provided by the Federal Commissioner to the data subject may not provide any indication of the knowledge available to the controller without its consent.

(7) Information shall be provided free of charge.

Section 19a Notification

(1) If data are collected without the data subject's knowledge, he or she shall be notified of such recording, the identity of the controller and the purposes of collection, processing or use. The data subject shall also be notified of recipients or categories of recipients except where he or she must expect transfer to such recipients. If a transfer is planned, notification shall be provided no later than the first transfer.

(2) Notification shall not be required if

1. the data subject already has this information,
2. notifying the data subject would involve a disproportionate effort, or
3. recording or transfer of personal data is expressly laid down by law.

The controller shall stipulate in writing the conditions under which notification shall not be provided in accordance with no. 2 or 3.

(3) Section 19 (2) through (4) shall apply accordingly.

Section 20 Rectification, erasure and blocking of data; right to object

(1) Personal data shall be rectified if they are inaccurate. If personal data neither processed in automated form nor recorded in non-automated filing systems are found to be inaccurate, or if the data subject disputes their accuracy, this shall be documented in an appropriate manner.

(2) Personal data processed in automated form or recorded in non-automated filing systems shall be erased if

1. unlawfully recorded, or
2. the controller no longer needs them to carry out the tasks for which it is responsible.

(3) Instead of being erased, data shall be blocked if

1. erasure would violate retention periods set by law, statute or contract,
2. there is reason to believe that erasure would adversely affect legitimate interests of the data subject, or
3. erasure would be impossible or would involve a disproportionate effort due to the special category of recording.

(4) Further, personal data processed in automated form or recorded in non-automated filing systems shall be blocked if the data subject disputes their accuracy and their accuracy or inaccuracy cannot be verified.

(5) Personal data may not be collected, processed or used for processing in automated form or in non-automated filing systems if the data subject lodges an objection with the controller and examination indicates that legitimate interests of the data subject due to his particular personal situation override the interest of the controller in such collection, processing or use. The first sentence shall not apply if collection, processing or use is required by law.

(6) Personal data which are neither processed in automated form nor recorded in non-automated filing systems shall be blocked if the authority finds in the particular case that, in the absence of blocking, legitimate interests of the data subject would be adversely affected and the data are no longer needed for the authority to carry out its tasks.

(7) Blocked data may be transferred or used without the data subject's consent only if

1. it is vital for scientific purposes, to supply necessary evidence, or for other reasons in the overriding interest of the controller or a third party, and
2. the transfer or use of the data for this purpose would be allowed if the data were not blocked.

(8) The bodies to which these data were transferred for recording shall be informed of the rectification of inaccurate data, the blocking of disputed data and erasure or blocking due to unlawful recording, if this does not involve a disproportionate effort and does not conflict with legitimate interests of the data subject.

(9) Section 2 (1) through (6), (8) and (9) of the Federal Archives Act shall be applied accordingly.

Section 21 Appeals to the Federal Commissioner for Data Protection and Freedom of Information

Anyone who believes his or her rights have been infringed through the collection, processing or use of his or her personal data by public bodies of the Federation may appeal to the Federal Commissioner for Data Protection and Freedom of Information. This shall apply to the collection, processing or use of personal data by federal courts only where they are active in administrative matters.

Chapter 3

Federal Commissioner for Data Protection and Freedom of Information

Section 22 Election of the Federal Commissioner for Data Protection and Freedom of Information

(1) At the proposal of the Federal Government, the German Bundestag shall elect the Federal Commissioner for Data Protection and Freedom of Information with more than half of the statutory number of its members. The Federal Commissioner must be at least 35 years old at the time of election. The person elected shall be appointed by the Federal President.

(2) The Federal Commissioner shall swear the following oath before the Federal Minister of the Interior:

> “I swear to do everything in my power to further the good and the benefit of the German people, to protect them from harm and to defend the Basic Law and the laws of the Federation, to perform my duties conscientiously and to exercise justice in all my dealings, so help me God.”

The reference to God may be omitted from the oath.

(3) The Federal Commissioner’s term of office shall be five years. It may be renewed once.

(4) The Federal Commissioner shall, in accordance with this Act, have official federal status under public law. He or she shall be independent in the performance of his or her duties and subject only to the law. He or she shall be subject to the legal supervision of the Federal Government.

(5) The Federal Commissioner shall be established at the Federal Ministry of the Interior. He or she shall be subject to the administrative supervision of the Federal Ministry of the Interior. The Federal Commissioner shall be provided with the staff and material resources necessary to carry out his or her tasks; these resources shall be shown in a separate chapter of the budget of the Federal Minister of the Interior. The posts shall be filled in agreement with the Federal Commissioner. Staff members who do not agree to the intended measure may be transferred, seconded or reassigned only in agreement with the Federal Commissioner.

(6) If the Federal Commissioner is temporarily prevented from performing his or her duties, the Federal Minister of the Interior may appoint a deputy to perform these duties. The Federal Commissioner shall be consulted on the appointment.

Section 23 Legal status of the Federal Commissioner for Data Protection and Freedom of Information

(1) The mandate of the Federal Commissioner for Data Protection and Freedom of Information shall commence on delivery of the certificate of appointment. It shall end

1. on expiry of the term of office,
2. on dismissal.

The Federal President shall dismiss the Federal Commissioner at the latter's request or at the suggestion of the Federal Government where there are grounds which would justify dismissal from service in the case of a judge with life tenure. In the event that the appointment is ended, the Federal Commissioner shall be given a document signed by the Federal President. Dismissal shall be effective on delivery of this document. At the request of the Federal Minister of the Interior, the Federal Commissioner shall be obligated to continue his or her work until a successor has been appointed.

(2) The Federal Commissioner shall not hold any other paid office or pursue any commercial activity or occupation in addition to his or her official duties and shall not belong to the management or supervisory board of a profit-oriented enterprise, nor to a government or legislative body of the Federation or a *Land*. The Federal Commissioner may not deliver extra-judicial opinions in exchange for payment.

(3) The Federal Commissioner shall inform the Federal Ministry of the Interior of any gifts received in connection with his or her office. The Federal Ministry of the Interior shall decide how such gifts shall be used.

(4) The Federal Commissioner shall have the right to refuse to give testimony concerning persons who have confided in him/her in his/her capacity as Federal Commissioner and concerning the information confided. This shall also apply to the staff of the Federal Commissioner, on the condition that the Federal Commissioner decides on the exercise of this right. Within the scope of the Federal Commissioner's right of refusal to give testimony, he or she may not be required to submit or surrender files or other documents.

(5) Even after his or her appointment has ended, the Federal Commissioner shall be obligated to secrecy concerning matters of which he/she is aware by reason of his/her duties. This shall not apply to official communications or to matters which are common knowledge or which by their nature do not require confidentiality. Even after leaving office, the Federal Commissioner may not testify in or outside court or make statements concerning such matters without the permission of the Federal Ministry of the Interior. This shall not affect the legal obligation to report crimes and to uphold the free and democratic order wherever it is threatened. Sections 93, 97, 105 (1), Section 111 (5) in conjunction with Section 105 (1) and Section 116 (1) of the German Fiscal Code shall not apply to the Federal Commissioner and his or her staff. The fifth sentence shall not apply where the financial authorities require such knowledge in order to conduct legal proceedings due to a tax offence and related tax proceedings, in the prosecution of which there is compelling public interest, or where the person required to provide information or persons acting on his/her behalf have intentionally provided false information. If the Federal Commissioner determines that data protection provisions have been violated, he or she shall be authorized to report the violation and inform the data subject accordingly.

(6) Permission to testify as a witness may be refused only if the testimony would be detrimental to the welfare of the Federation or a *Land*, or would seriously endanger or significantly interfere with the execution of public duties. Permission to provide a report may be refused if providing a report would be detrimental to official interests. Section 28 of the Act on the Federal Constitutional Court shall remain unaffected.

(7) From the start of the calendar month in which the Federal Commissioner's appointment commences until the end of the calendar month in which his/her appointment ends, or, in the case of subsection 1 sixth sentence, until the end of the month in which he/she ceases his/her work, the Federal Commissioner shall be paid at the level of a federal civil servant in pay grade B 9. The Federal Travel Expenses Act and the Federal Relocation Expenses Act shall apply accordingly. In all other respects, Section 12 (6), Sections 13 through 20 and 21a (5) of the Act on Federal Ministers shall apply, except that the four-year term of office stipulated in Section 15 (1) of the Act on Federal Ministers shall be replaced by a five-year term and pay grade B 11 stipulated in Section 21a (5) of the Act on Federal Ministers shall be replaced by pay grade B 9. By way of derogation from the third sentence in conjunction with Sections 15 through 17 and 21a (5) of the Act on Federal Ministers, the Federal Commissioner's pension shall be calculated, counting his or her term as Federal Commissioner as a pensionable period of service, on the basis of the Federal Act Governing Civil Servants' Pensions and Allowances, if this is more favourable and if, before his or election as Federal Commissioner, he or she was a civil servant or judge in at least the last position to be held before reaching pay grade B 9.

(8) Subsection 5, fifth through seventh sentences shall apply accordingly to the public bodies responsible for monitoring compliance with data protection provisions in the *Länder*.

Section 24 Monitoring by the Federal Commissioner for Data Protection and Freedom of Information

(1) The Federal Commissioner for Data Protection and Freedom of Information shall monitor compliance by the public bodies of the Federation with the provisions of this Act and other data protection provisions.

(2) Monitoring by the Federal Commissioner shall also extend to

1. personal data obtained by public bodies of the Federation concerning the contents of and specific circumstances relating to postal communications and telecommunications, and
2. personal data subject to professional or special official secrecy, especially tax secrecy under Section 30 of the German Fiscal Code.

The fundamental right to privacy of correspondence, posts and telecommunications in Article 10 of the Basic Law shall be thus restricted. Personal data subject to monitoring by the commission established under Section 15 of the Act to Restrict the Privacy of Correspondence, Posts and Telecommunications shall not be subject to monitoring by the Federal Commissioner unless the commission requests the Federal Commissioner to monitor compliance with data protection provisions in connection with specific procedures or in specific areas and to report solely to the commission. Personal data in files on background security checks shall not be subject to monitoring by the Federal Commissioner if the data subject lodges an

objection with the Federal Commissioner concerning the monitoring of data relating to the data subject in the particular case.

(3) The federal courts shall be subject to monitoring by the Federal Commissioner only where they are active in administrative matters.

(4) The public bodies of the Federation shall be obligated to assist the Federal Commissioner and his or her assistants in performing their duties. In particular, they shall be given

1. information in reply to their questions, as well as the opportunity to inspect all documents and especially recorded data and data processing programs in connection with monitoring under subsection 1,
2. access to all official premises at all times.

The authorities referred to in Section 6 (2) and Section 19 (3) shall provide assistance only to the Federal Commissioner him- or herself and assistants specially designated by him or her in writing. The second sentence shall not apply to these authorities where the supreme federal authority finds in the particular case that the information or inspection would endanger the security of the Federation or a *Land*.

(5) The Federal Commissioner shall inform the public body of the monitoring results. The Federal Commissioner may include recommendations for improving data protection, especially for remedying problems found in the processing or use of personal data. Section 25 shall remain unaffected.

(6) Subsection 2 shall apply accordingly to the public bodies responsible for monitoring compliance with data protection provisions in the *Länder*.

Section 25 Complaints lodged by the Federal Commissioner for Data Protection and Freedom of Information

(1) If the Federal Commissioner for Data Protection and Freedom of Information finds violations of the provisions of this Act or other data protection provisions or other problems with the processing or use of personal data, he or she shall lodge a complaint

1. in the case of the federal administration: with the competent supreme federal authority,
2. in the case of the Federal Railway Property Agency: with its president,
3. in the case of successor companies created by law from the Special Fund “Deutsche Bundespost” as long as they have an exclusive right under the Postal Act: with their boards of directors,
4. in the case of direct federal corporations, institutions and foundations under public law as well as their associations: their boards of directors or other bodies authorized to represent them,

and shall require them to respond within a period to be determined by the Federal Commissioner. In the cases of the first sentence no. 4, the Federal Commissioner shall inform the responsible supervisory authority at the same time.

(2) The Federal Commissioner may dispense with a complaint or a response from the body concerned, especially if the problems involved are insignificant or have been remedied in the meantime.

(3) The response should also describe the measures taken as a result of the Federal Commissioner’s complaint. The bodies referred to in subsection 1, first sentence no. 4 shall provide the competent supervisory authority a copy of their response to the Federal Commissioner at the same time.

Section 26 Additional duties of the Federal Commissioner for Data Protection and Freedom of Information

(1) The Federal Commissioner for Data Protection and Freedom of Information shall submit an activity report to the German Bundestag every two years. The report shall inform the German Bundestag and the public about important developments in the field of data protection.

(2) At the request of the German Bundestag or the Federal Government, the Federal Commissioner shall draft expert opinions and provide reports. At the request of the German Bundestag, the Petitions Committee, the Bundestag’s Committee on Internal Affairs or the Federal Government, the Federal Commissioner shall also investigate data protection matters and incidents at public bodies of the Federation. The Federal Commissioner may consult the German Bundestag at any time.

(3) The Federal Commissioner may make recommendations to the Federal Government and the federal bodies referred to in Section 12 (1) for improving data protection and may advise them in matters of data protection. The Federal Commissioner shall inform the bodies referred to in Section 25 (1) nos. 1 through 4 if the recommendation or advice does not concern them directly.

(4) The Federal Commissioner shall work to cooperate with the public bodies responsible for monitoring compliance with data protection provisions in the *Länder* and with the supervisory authorities under Section 38. Section 38 (1) third and fourth sentences shall apply accordingly.

(4) The Federal Commissioner shall work to cooperate with the public bodies responsible for monitoring compliance with data protection provisions in the Länder and with the supervisory authorities under Section 38. Section 38 (1) fourth and fifth sentences shall apply accordingly.[6]

Part III

Data processing by private bodies and commercial enterprises under public law

Chapter 1

Legal basis for data processing

Section 27 Scope

(1) The provisions of this Part shall apply as far as personal data are processed or used by means of data processing systems or collected for that purpose, or data are processed or used in or from non-automated filing systems or collected for that purpose by

---

[6] In force from 1 April 2010.

1. private bodies,
2. a) public bodies of the Federation, where they are engaged in competition as commercial enterprises under public law,

   b) public bodies of the *Länder*, where they are engaged in competition as commercial enterprises under public law, execute federal law and data protection is not governed by *Land* law.

This shall not apply where data are collected, processed or used solely for personal or domestic activities. In cases of no. 2 a, Sections 18, 21 and 24 through 26 shall apply in place of Section 38.

(2) The provisions of this Part shall not apply to the processing and use of personal data outside of non-automated filing systems, as long as the personal data have not clearly been obtained from automated processing.

Section 28 Collection and recording of data for own commercial purposes

(1) The collection, recording, alteration or transfer of personal data or their use as a means to pursue own commercial purposes shall be lawful

1. if necessary to create, perform or terminate a legal obligation or quasi-legal obligation with the data subject,
2. as far as necessary to safeguard legitimate interests of the controller and there is no reason to assume that the data subject has an overriding legitimate interest in ruling out the possibility of processing or use, or
3. if the data are generally accessible or the controller would be allowed to publish them, unless the data subject has a clear and overriding legitimate interest in ruling out the possibility of processing or use.

When personal data are collected, the purposes for which the data are to be processed or used shall be specifically defined.

(2) The transfer or use for another purpose shall be lawful

1. under the conditions given in subsection 1 first sentence no. 2 or no. 3,
2. where necessary

   a) to safeguard legitimate interests of a third party, or

   b) to prevent threats to state or public security or to prosecute crimes

   and there is no reason to believe that the data subject has a legitimate interest in ruling out the possibility of transfer or use, or
3. if necessary in the interest of a research institution for the purpose of scientific research, where the scientific interest in carrying out the research project significantly outweighs the data subject’s interest in ruling out the possibility of collection, and the purpose of the research cannot be achieved in any other way or would require a disproportionate effort.

(3) The processing or use of personal data for purposes of advertising or trading in addresses shall be lawful if the data subject has given his or her consent and, if such consent was not given in written form, the controller proceeds in accordance with subsection 3a. In addition, processing or use of personal data shall be lawful where the data consist of lists or other summaries of data from members of a category of

persons defined only in terms of the data subject's membership of this category, his or her occupation, name, title, academic degree(s), address and year of birth, and where processing or use is necessary

1. for purposes of advertising from the controller which collected the data, except for information on membership of the category, from the data subject under subsection 1 first sentence no. 1 or from publicly accessible sources such as telephone or business directories,
2. for purposes of advertising in view of the data subject's occupation and at his or her work address, or
3. for purposes of soliciting donations eligible for tax concessions under Section 10b (1) and Section 34g of the Income Tax Act.

For purposes under the second sentence no. 1, the controller may record data in addition to the data referred to there. Summarized personal data under the second sentence may also be transferred for advertising purposes if the transfer is recorded in accordance with Section 34 (1a) first sentence; in this case, the advertisement must clearly identify the body which first collected the data. Regardless of whether the conditions of the second sentence are met, personal data may be used for advertising third-party offers if the data subject can clearly identify from the advertisement the controller responsible for using the data. Processing or use as referred to in the second through fourth sentences shall be lawful only where it does not conflict with legitimate interests of the data subject. Data transferred under the first, second and fourth sentences may be processed or used only for the purpose for which they were transferred.

(3a) If consent under Section 4a (1) third sentence is given in a form other than writing, the controller shall provide the data subject with written confirmation of the substance of the consent unless consent was given in electronic form and the controller ensures that the declaration of consent is recorded and the data subject can access and revoke it at any time with future effect. If consent is to be given together with other written declarations, it shall be made distinguishable in its printing and format.

(3b) The controller may not make the conclusion of a contract dependent on the data subject's consent under subsection 3 first sentence, if access to equivalent contractual benefits is impossible or unreasonable without providing consent. Consent provided under such circumstances shall be invalid.

(4) If the data subject lodges an objection with the controller regarding the processing or use of his or her data for advertising purposes or market or opinion research, processing or use for these purposes shall be unlawful. In approaching the data subject for the purpose of advertising or market or opinion research, and in the cases of subsection 1 first sentence no. 1 also when creating a legal or quasi-legal obligation, the data subject shall be informed of the identity of the controller and of the right to object under the first sentence; where the party approaching the data subject uses the data subject's personal data recorded by a body unknown to him or her, the approaching party shall also ensure that the data subject may obtain information about the source of the data. If the data subject lodges an objection with the third party to which the data were transferred in connection with purposes under subsection 3 as to the processing or use for purposes of advertising or market or opinion research, the third party shall block the data for these purposes. In the cases

of subsection 1 first sentence no. 1, the requirements as to the form of the objection may not be stricter than for the creation of a legal or quasi-legal obligation.

(5) The third party to which the data are transferred may process or use these data only for the purpose for which they were transferred. Private bodies may process or use the data for other purposes only subject to the conditions of subsections 2 and 3, and public bodies may process or use the data for other purposes only subject to the conditions of Section 14 (2). The transferring body shall point this out to the third party.

(6) The collection, processing and use of special categories of personal data (Section 3 (9)) for own commercial purposes shall be lawful without the data subject's consent in accordance with Section 4a (3) if

1. necessary to protect the vital interests of the data subject or of another person where the data subject is physically or legally incapable of giving his or her consent,
2. data are involved which the data subject has manifestly made public,
3. necessary to assert, exercise or defend legal claims and there is no reason to assume that the data subject has an overriding legitimate interest in ruling out the possibility of collection, processing or use, or
4. necessary for the purposes of scientific research, where the scientific interest in carrying out the research project significantly outweighs the data subject's interest in ruling out the possibility of collection, processing and use and the purpose of the research cannot be achieved in any other way or would require a disproportionate effort.

(7) The collection of special categories of personal data (Section 3 (9)) shall further be lawful when required for the purposes of preventive medicine, medical diagnosis, the provision of care or treatment or the management of health-care services, and where these data are processed by health professionals subject to the obligation of professional secrecy or by other persons also subject to an equivalent obligation of secrecy. The processing and use of data for the purposes referred to in the first sentence shall be subject to the obligation of secrecy which applies to the persons referred to in the first sentence. The collection, processing or use of data concerning the health of persons for a purpose listed in the first sentence by members of a profession other than those listed in Section 203 (1) and (3) of the Criminal Code which involves identifying, healing or relieving illnesses, or producing or selling aids shall be lawful only subject to the same conditions authorizing a physician to do so.

(8) Special categories of personal data (Section 3 (9)) may be transferred or used for other purposes only subject to the conditions of subsection 6 nos. 1 through 4 or of subsection 7 first sentence. Transfer or use shall be lawful also if necessary to prevent threats to state and public security or to prosecute serious crimes.

(9) Non-profit-seeking organizations with a political, philosophical, religious or trade-union aim may collect, process or use special categories of personal data (Section 3 (9)) as needed for their activities. This shall apply only to the personal data of their members or to persons who have regular contact with them in connection with their purposes. Transferring these personal data to persons or bodies outside the organization shall be lawful only subject to the conditions of Section 4a (3). Subsection 2 no. 2 b shall apply accordingly.

Section 28a Data transfer to rating agencies

(1) Transferring personal data concerning a claim to rating agencies shall be lawful only if the performance owed has not been rendered on time, the transfer is necessary to safeguard the legitimate interests of the controller or a third party and

1. *the claim has been established by a final decision or a decision declared enforceable for the time being, or if an executory title has been issued under Section 794 of the Code of Civil Procedures,*
2. *the claim has been established under Section 178 of the Insolvency Act and has not been disputed by the debtor at the verification meeting,*
3. *the data subject has expressly acknowledged the claim,*
4. *a) the data subject received at least two written reminders after the due date,*
   b) at least four weeks elapsed between the first reminder and the data transfer,
   c) the controller gave the data subject sufficient notice before transferring the information, or at least informed the data subject of the impending transfer in the first reminder, and
   d) the data subject did not dispute the claim, or
5. *the contractual relationship on which the claim is based can be terminated without prior notice for payment in arrears and the controller has informed the data subject of the impending transfer.*

The first sentence shall apply accordingly if the controller itself uses the data under Section 29.

(2) For the future transfer under Section 29 (2), credit institutions may transfer to rating agencies personal data about the creation, orderly execution and termination of a contractual relationship concerning a bank transaction as referred to in Section 1 (1) second sentence, no. 2, 8, or 9 of the Banking Act unless the data subject's legitimate interest in ruling out the possibility of transfer manifestly overrides the interest of the rating agency in knowledge of the data. The data subject shall be informed of this before the contract is concluded. The first sentence shall not apply to contracts concerning current accounts without overdraft protection. For the future transfer under Section 29 (2), the transfer of data concerning the behaviour of data subjects which serve to create market transparency in the context of pre-contractual relationships of trust shall be unlawful, even with the data subject's consent.

(3) Within one month of becoming aware of any subsequent alteration of facts based on a transfer conducted in accordance with subsection 1 or 2, the controller shall inform the rating agency of such alteration, as long as the rating agency still has the data originally transferred. The rating agency shall inform the body which transferred the data when it has erased the data originally transferred.[7]

[7] *In force from 1 April 2010.*

Section 28b Scoring

For the purpose of deciding on the creation, execution or termination of a contractual relationship with the data subject, a probability value for certain future action by the data subject may be calculated or used if

1. *the data used to calculate the probability value are demonstrably essential for calculating the probability of the action on the basis of a scientifically recognized mathematic-statistical procedure,*
2. *if the probability value is calculated by a rating agency, the conditions for transferring the data used under Section 29, and in all other cases the conditions of lawful use of data under Section 28 are met,*
3. *the probability value is not calculated solely on the basis of address data,*
4. *if address data are used, the data subject shall be notified in advance of the planned use of these data; this notification shall be documented.*[8]

Section 29 Commercial data collection and recording for the purpose of transfer

(1) Commercial collection, recording or alteration of personal data for the purpose of transfer, in particular for the purpose of advertising, the activities of rating agencies or trade in addresses, shall be lawful if

1. there is no reason to believe that the data subject has a legitimate interest in ruling out the possibility of collection, recording or alteration, or
2. the data can be acquired from generally accessible sources or the controller would be allowed to publish them, unless the data subject has a clear and overriding legitimate interest in ruling out the possibility of collection, recording or alteration.

Section 28 (1) first sentence and subsections 3 through 3b shall apply.

(1) The commercial collection, recording, alteration or use of personal data for the purpose of transfer, in particular for the purpose of advertising, the activities of rating agencies or trade in addresses, shall be lawful if

1. *there is no reason to believe that the data subject has a legitimate interest in ruling out the possibility of collection, recording or alteration,*
2. *the data can be acquired from generally accessible sources or the controller would be allowed to publish them, unless the data subject has a clear and overriding legitimate interest in ruling out the possibility of collection, recording or alteration, or*
3. *the conditions of Section 28a (1) or (2) are met; data as defined in Section 28a (2) fourth sentence may not be collected or recorded.*

Section 28 (1) second sentence and subsections 3 through 3b shall apply.[9]

(2) Transfer for the purposes specified in subsection 1 shall be lawful if

---

[8] In force from 1 April 2010.

[9] In force from 1 April 2010.

1. the third party to which the data are transferred has presented credible evidence of legitimate interest in knowledge of the data, and
2. there is no reason to believe that the data subject has a legitimate interest in ruling out the possibility of transfer.

Section 28 (3) through (3b) shall apply accordingly. In the case of transfer in accordance with no. 1, the evidence of legitimate interest and the means of presenting this evidence in a credible way shall be documented by the transferring body. In the case of transfer using automated retrieval, the third party to which the data are transferred shall be responsible for documentation.

(2) Transfer for the purposes specified in subsection 1 shall be lawful if

1. *the third party to which the data are transferred has presented credible evidence of legitimate interest in knowledge of the data, and*
2. *there is no reason to believe that the data subject has a legitimate interest in ruling out the possibility of transfer.*

Section 28 (3) through (3b) shall apply accordingly. In the case of transfer in accordance with the first sentence no. 1, the evidence of legitimate interest and the means of presenting this evidence in a credible way shall be documented by the transferring body. In the case of transfer using automated retrieval, the third party to which the data are transferred shall be responsible for documentation. The transferring body shall take random samples in accordance with Section 10 (4) third sentence and in doing so also determine whether a legitimate interest exists in the particular case.[10]

(3) Personal data shall not be included in electronic or printed address, telephone, business or similar directories if it is clear from the electronic or printed directory that such inclusion is contrary to the data subject’s will. The recipient of the data shall ensure that identifiers from electronic or printed directories or registers are retained upon their inclusion in directories or registers.

(4) Section 28 (4) and (5) shall apply to the processing or use of the transferred data.

(5) Section 28 (6) through (9) shall apply accordingly.

(6) Any body which for the purpose of transfer commercially collects, records or alters personal data which may be used to evaluate the creditworthiness of consumers shall treat requests for information from lenders in other European Union Member States or other states party to the Agreement on the European Economic Area the same way it treats information requests from domestic lenders.[11]

(7) Anyone who refuses to conclude a consumer loan contract or a contract concerning financial assistance for payment with a consumer as the result of information provided by a body as referred to in subsection 6 shall immediately notify the consumer of this refusal and the information received. Such notification shall not be made if it would endanger public security or order. Section 6a shall remain unaffected.[12]

---

[10] In force from 1 April 2010.

[11] In force from 11 June 2010.

[12] In force from 11 June 2010.

Section 30 Commercial data collection and recording for the purpose of transfer in anonymous form

(1) If personal data are commercially collected and recorded for the purpose of transfer in anonymous form, those features enabling personal or material circumstances to be attributed to an identified or identifiable natural person shall be recorded separately. These features may be combined with the information only where necessary for the purpose for which the data are recorded or for scientific purposes.

(2) The alteration of personal data shall be lawful if

1. there is no reason to believe that the data subject has a legitimate interest in ruling out the possibility of alteration, or
2. the data can be acquired from generally accessible sources or the controller would be allowed to publish them, unless the data subject has a clear and overriding legitimate interest in ruling out the possibility of alteration.

(3) The personal data shall be erased if they were unlawfully recorded.

(4) Section 29 shall not apply.

(5) Section 28 (6) through (9) shall apply accordingly.

Section 30a Commercial data collection and recording for purposes of market or opinion research

(1) The commercial collection, processing or use of personal data for purposes of market or opinion research shall be lawful if

1. there is no reason to believe that the data subject has a legitimate interest in ruling out the possibility of collection, processing or use, or
2. the data can be acquired from generally accessible sources or the controller would be allowed to publish them, and the data subject's legitimate interest in ruling out the possibility of collection, processing or use does not clearly override the interest of the controller.

Special categories of personal data (Section 3 (9)) may be collected, processed or used only for a specific research project.

(2) Personal data collected or recorded for purposes of market or opinion research may be processed or used only for these purposes. Data which were not acquired from generally accessible sources and which the controller may not publish may be processed or used only for the research project for which they were collected. They may be processed or used for a different purpose only if they have been rendered anonymous in such a way that the identification of the data subject is no longer possible.

(3) Personal data shall be rendered anonymous as soon as allowed by the purpose of the research project for which they were collected. Until then, the features enabling information concerning personal or material circumstances attributable to an identified or identifiable person shall be kept separately. These features may be combined with the information only where necessary for the purpose of the research project.

(4) Section 29 shall not apply.

(5) Section 28 (4) and (6) through (9) shall apply accordingly.

Section 31 Special restrictions on use

Personal data recorded exclusively for purposes of monitoring data protection, safeguarding data or ensuring proper operation of a data processing system may only be used for these purposes.

Section 32 Data collection, processing and use for employment-related purposes

(1) An employee's personal data may be collected, processed or used for employment-related purposes where necessary for hiring decisions or, after hiring, for carrying out or terminating the employment contract. Employees' personal data may be collected, processed or used to investigate crimes only if there is a documented reason to believe the data subject has committed a crime while employed, the collection, processing or use of such data is necessary to investigate the crime, and the employee does not have an overriding legitimate interest in ruling out the possibility of collection, processing or use, and in particular the type and extent are not disproportionate to the reason.

(2) Subsection 1 shall also apply when personal data are collected, processed or used without the help of automated processing systems, or are processed or used in or from a non-automated filing system or collected in such a filing system for processing or use.

(3) The rights of participation of employee staff councils shall remain unaffected.

Chapter 2

Rights of the data subject

Section 33 Notification of the data subject

(1) If personal data are recorded for own purposes for the first time without the data subject's knowledge, the data subject shall be notified of such recording, the type of data, the purpose of collection, processing or use and the identity of the controller. If personal data are commercially recorded for the purpose of transfer without the data subject's knowledge, the data subject shall be notified of their initial transfer and of the type of data transferred. In the cases covered by the first and second sentences above, the data subject shall also be notified of the categories of recipients, where, given the circumstances of the individual case, the data subject need not expect that his/her data will be transferred to such recipients.

(2) Notification shall not be required if

1. the data subject has become aware of the recording or transfer by other means,
2. the data were recorded only because they may not be erased due to legal, statutory or contractual provisions on retention, or only for purposes of monitoring data protection or safeguarding data, and providing information would require a disproportionate effort,
3. the data must be kept secret by law or due to the nature of the data, namely due to the overriding legal interests of a third party,

4. recording or transfer is expressly laid down by law,
5. recording or transfer is necessary for the purposes of scientific research and notification would require a disproportionate effort,
6. the responsible public body has informed the controller that disclosure of the data would threaten the public security or order or otherwise be detrimental to the Federation or a *Land*, or
7. the data were recorded for own purposes and
   a) were acquired from generally accessible sources and notification would require a disproportionate effort due to the large number of cases concerned, or
   b) notification would seriously endanger the commercial purposes of the controller, unless the interest in notification overrides this danger,
8. The data were commercially recorded for the purpose of transfer, and
   a) were acquired from generally accessible sources, where they related to the persons who published the data, or
   b) the data are compiled in lists or otherwise summarized (Section 29 (2) second sentence)

   and notification would require a disproportionate effort due to the large number of cases concerned,
9. data acquired from generally accessible sources recorded commercially for the purpose of market or opinion research and notification would require a disproportionate effort due to the large number of cases concerned.

The controller shall stipulate in writing the conditions under which notification shall not be provided in accordance with the first sentence, nos. 2 through 7.

Section 34 Access to data

(1) Data subjects may request information about

1. recorded data relating to them, including information relating to the source of the data,
2. the recipients or categories of recipients to which the data are transferred, and
3. the purpose of recording the data.

Data subjects should specify the type of personal data on which information is to be given. If the personal data are commercially recorded for the purpose of transfer, data subjects may request information about the source and recipients only if there is no overriding interest in protecting trade secrets. In this case, data subjects shall be given information about the source and recipients even if this information was not recorded.

(2) Data subjects may request information about their personal data from bodies which commercially record personal data for the purpose of providing information even when the data are not recorded in an automated processing system or a non-automated filing system. Data subjects may request information about the source and recipients only if there is no overriding interest in protecting trade secrets.

(3) Information shall be provided in writing unless special circumstances warrant any other form.

(4) There shall be no obligation to provide information if Section 33 (2) first sentence nos. 2, 3 and 5 through 7 do not require notifying the data subject.

(5) Information shall be provided free of charge. However, if personal data are commercially recorded for the purpose of transfer, a fee may be charged if the data subject can use the information vis-à-vis third parties for commercial purposes. The fee shall not exceed the costs resulting directly from the provision of information. No fee may be charged in cases where special circumstances give reason to believe that the data are incorrectly or unlawfully recorded, or where the information provided indicates that the data shall be rectified or erased subject to Section 35 (2) second sentence no. 1.

(6) Where a fee is charged for providing information, data subjects shall have the option of personal access to the data and information concerning them within the framework of their right of access. Data subjects shall be informed of this option in an appropriate form.

Section 34 Access to data

(1) The controller shall provide information to data subjects at their request concerning

1. *recorded data relating to them, including information relating to the source of the data,*
2. *the recipients or categories of recipients to which the data are transferred, and*
3. *the purpose of recording the data.*

Data subjects should specify the type of personal data about which information is to be given. If the personal data are commercially recorded for the purpose of transfer, information shall be provided about the source and recipients even if this information is not recorded. Information about the source and recipients may be withheld if the interest in protecting trade secrets overrides the data subject's interest in the information.

(1a) In the case of Section 28 (3) fourth sentence, the transferring body shall record the source of the data and the recipient for two years following transfer and shall provide the data subject with information about the source of the data and the recipient upon request. The first sentence shall apply to the recipient accordingly.

(2) In the case of Section 28b, the body responsible for the decision shall provide information to data subjects at their request concerning

1. *probability values calculated or recorded for the first time within the six months preceding the receipt of the information request,*
2. *the types of data used to calculate the probability values, and*
3. *how probability values are calculated and their significance, with reference to the individual case and in generally understandable terms.*

The first sentence shall apply accordingly if the body responsible for the decision

1. records the data used to calculate the probability values without reference to specific persons but creates such a reference when calculating the probability value, or

2. uses data recorded by another body.

If a body other than the body responsible for the decision calculates

1. the probability value or

2. a component of the probability value,

it shall provide the body responsible for the decision with the information necessary to answer the request as referred to in the first and second sentences upon request.

In the case of the third sentence no. 1, in responding to a request for information, the body responsible for the decision shall, without delay, provide the data subject with the name and address of the other body as well as the information necessary to reference the individual case, unless the body responsible for the decision provides the requested information itself. In this case, the other body which calculated the probability value shall provide the data subject with the desired information as referred to in the first and second sentences free of charge. The body responsible for calculating the probability value shall not be subject to the obligation referred to in the third sentence if the body responsible for the decision uses its right referred to in the fourth sentence.

(3) A body which commercially records personal data for the purpose of transfer shall provide data subjects with information about data relating to them, even if these data are neither processed in automated form nor recorded in non-automated filing systems. Data subjects shall also be given information about data

1. which currently demonstrate no reference to specific persons although the controller is to create such a reference in connection with providing information,

2. which the controller does not record but uses for the purpose of providing information.

Information about the source and recipients may be withheld if the interest in protecting trade secrets overrides the data subject's interest in the information.

(4) A body which commercially collects, records or alters personal data for the purpose of transfer shall, upon request, provide data subjects with information about

1. probability values for certain future action by the data subject which were transferred within the twelve months preceding the receipt of the information request, as well as the names and last-known addresses of third parties to which the values were transferred,

2. the probability values at the time of the information request calculated according to the method used by the calculating body,

3. the types of data used to calculate the probability values referred to in nos. 1 and 2, and

4. how probability values are calculated and their significance, with reference to the individual case and in generally understandable terms.

The first sentence shall apply accordingly if the controller

1. *records the data used to calculate the probability value without reference to specific persons but creates such a reference when calculating the probability value, or*
2. *uses data recorded by another body.*

(5) Data recorded for the purpose of providing information to data subjects in accordance with subsections 1a through 4 may be used only for this purpose and for the purpose of monitoring data protection; they shall be blocked for other purposes.

(6) Information shall be provided in writing upon request unless special circumstances make another form appropriate.

(7) There shall be no obligation to provide information if Section 33 (2) first sentence nos. 2, 3 and 5 through 7 do not require notifying the data subject.

(8) Information shall be provided free of charge. If the personal data are commercially recorded for the purpose of transfer, the data subject may request information in writing free of charge once per calendar year. A fee may be charged for each additional request if the data subject can use the information vis-à-vis third parties for commercial purposes. The fee shall not exceed the costs resulting directly from the provision of information. No fee may be charged if

1. *special circumstances give reason to believe that the data are incorrectly or unlawfully recorded, or*
2. *the information provided indicates that the data shall be rectified in accordance with Section 35 (1) or erased in accordance with Section 35 (2) second sentence no. 1.*

(9) Where a fee is charged for providing information, data subjects shall have the option of personal access to the data concerning them within the framework of their right of access. Data subjects shall be informed of this option.[13]

Section 35 Correction, deletion and blocking of data

(1) Personal data shall be rectified when they are inaccurate.

Estimated data shall be clearly identified as such.[14]

(2) Personal data may be erased at any time, except in the cases referred to in subsection 3 nos. 1 and 2. Personal data shall be erased if

1. unlawfully recorded, or
2. they concern racial or ethnic origin, political opinions, religious or philosophical beliefs, trade-union membership, health or sex life, punishable actions or administrative offences, and the controller is unable to prove their accuracy,
3. they are processed for own purposes, as soon as knowledge of them is no longer needed to carry out the purpose for which they were recorded, or

[13] In force from 1 April 2010.

[14] In force from 1 April 2010.

4. they are commercially processed for the purpose of transfer and an examination at the end of the fourth calendar year from their initial recording shows that further retention is unnecessary.

(2) Personal data may be erased at any time, except in the cases referred to in subsection 3 nos. 1 and 2. Personal data shall be erased if

1. unlawfully recorded, or

2. the data concern racial or ethnic origin, political opinions, religious or philosophical beliefs, trade-union membership, health, sex life, punishable actions or administrative offences, and the controller is unable to prove their accuracy,

3. they are processed for own purposes, as soon as knowledge of them is no longer needed to carry out the purpose for which they were recorded, or

4. they are processed commercially for the purpose of transfer and an examination at the end of the fourth calendar year following their initial recording, or at the end of the third calendar year in the case of data concerning matters that have been concluded, shows that further retention is unnecessary.

Personal data recorded on the basis of Section 28a (2) first sentence or Section 29 (1) first sentence no. 3 shall be erased after the termination of the contract, if the data subject so requests.[15]

(3) Instead of being erased, data shall be blocked where

1. in the case of subsection 2 no. 3, erasure would violate retention periods set by law, statute or contract,

2. there is reason to believe that erasure would be detrimental to legitimate interests of the data subject, or

3. erasure would be impossible or would involve a disproportionate effort due to the special category of recording.

(3) Instead of being erased, data shall be blocked where

1. in the case of subsection 2 second sentence no. 3, erasure would violate retention periods set by law, statute or contract,

2. there is reason to believe that erasure would be detrimental to legitimate interests of the data subject, or

3. erasure would be impossible or would involve a disproportionate effort due to the special category of recording.[16]

(4) Further, personal data shall be blocked if the data subject disputes their accuracy and their accuracy or inaccuracy cannot be verified.

(4a) The fact that the data are blocked shall not be disclosed.[17]

---

[15] In force from 1 April 2010.

[16] In force from 1 April 2010.

[17] In force from 1 April 2010.

(5) Personal data may not be collected, processed or used for processing in automated form or in non-automated filing systems if the data subject lodges an objection with the controller and examination indicates that legitimate interests of the data subject due to his or her particular personal situation override the interest of the controller in such collection, processing or use. The first sentence shall not apply if collection, processing or use is required by law.

(6) Where they are commercially recorded for the purpose of transfer, except in the cases referred to in subsection 2 no. 2, personal data which are incorrect or whose accuracy is disputed need not be rectified, blocked or erased if they were acquired from generally accessible sources and recorded for documentation purposes. At the data subject's request, his or her counter-statement shall be kept with these data for the duration of their retention. The data may not be transferred without this counter-statement.

(7) The bodies to which these data are transferred for recording shall be informed of the rectification of inaccurate data, the blocking of disputed data and erasure or blocking due to unlawful recording, if this does not involve a disproportionate effort and does not conflict with legitimate interests of the data subject.

(7) The bodies to which these data were transferred for recording shall be informed of the rectification of inaccurate data, the blocking of disputed data and erasure or blocking due to unlawful recording, if this does not involve a disproportionate effort and does not conflict with legitimate interests of the data subject.[18]

(8) Blocked data may be transferred or used without the data subject's consent only if

1. it is vital for scientific purposes, to supply necessary evidence, or for other reasons in the overriding interest of the controller or a third party, and
2. the transfer or use of the data for this purpose would be allowed if the data were not blocked.

Chapter 3

Supervisory authority

Section 36 (deleted)

Section 37 (deleted)

Section 38 Supervisory authority

(1) The supervisory authority shall monitor the implementation of this Act and other data protection provisions governing the automated processing of personal data or the processing or use of personal data in or from non-automated filing systems, including the rights of the Member States in the cases of Section 1 (5). It shall advise and support the data protection officials and controllers with due regard to their typical requirements. The supervisory authority may process and use data it has recorded for supervisory purposes only; Section 14 (2) nos. 1 through 3, 6 and 7 shall apply accordingly. In particular, the supervisory authority may transfer data to

[18] In force from 1 April 2010.

other supervisory authorities for supervisory purposes. On request, it shall provide supplementary assistance (administrative assistance) to the supervisory authorities of other Member States of the European Union. If the supervisory authority finds that this Act or other data protection provisions have been violated, it shall be authorized to notify the data subjects, to report the violation to the bodies responsible for prosecution or punishment, and, in case of serious violations, to notify the trade supervisory authority in order to initiate measures under trade law. It shall publish an activity report regularly and at least every two years. Section 21 first sentence and Section 23 (5) fourth through seventh sentences shall apply accordingly.

(2) The supervisory authority shall keep a register of the automated processing operations subject to the obligation to notify under Section 4d, to include the information specified in Section 4e first sentence. The register may be inspected by any person. The right to inspection shall not extend to the information referred to in Section 4e first sentence no. 9, nor to the identity of the persons entitled to access.

(3) The bodies subject to monitoring and the persons responsible for their management shall provide the supervisory authority, on request and without delay, the information necessary to perform its duties. The person required to provide information may refuse to answer those questions which would expose him-/herself or a relative as referred to in Section 383 (1) nos. 1 through 3 of the Code of Civil Procedure to the risk of criminal prosecution or proceedings under the Administrative Offences Act. The person required to provide information shall be informed accordingly.

(4) Persons appointed by the supervisory authority to conduct monitoring shall be authorized, where necessary for them to perform the duties assigned by the supervisory authority, to enter the property and premises of the body during business hours and to carry out checks and inspections there. They may inspect business documents, especially the list referred to in Section 4g (2) first sentence and the recorded personal data and data processing programs. Section 24 (6) shall apply accordingly. The person required to provide information shall allow these measures.

(5) To ensure compliance with this Act and other data protection provisions, the supervisory authority may order measures to remedy violations identified in the collection, processing or use of personal data, or technical or organizational problems. In case of serious violations or problems, especially those related to a special threat to privacy, the supervisory authority may prohibit collection, processing or use, or the use of particular procedures if the violations or problems are not remedied within a reasonable time despite orders as referred to in the first sentence and despite the imposition of a fine. The supervisory authority may demand the dismissal of a data protection official if he or she does not have the necessary specialized knowledge and reliability to perform his or her duties.

(6) The *Land* governments or the bodies authorized by them shall designate the supervisory authorities responsible for monitoring the implementation of data protection within the scope of this part.

(7) The application of the Industrial Code to commercial enterprises subject to the provisions of this Part shall remain unaffected.

Section 38a Codes of conduct to facilitate the application of data protection provisions

(1) Trade associations and other bodies representing specific categories of controllers may submit to the responsible supervisory authority draft codes of conduct to facilitate the application of data protection provisions.

(2) The supervisory authority shall examine the drafts submitted for compliance with applicable data protection law.

Part IV

Special provisions

Section 39 Restrictions on use of personal data subject to professional or special official secrecy

(1) Personal data subject to professional or special official secrecy and provided by the body obligated to secrecy in the performance of its professional or official duties may be processed or used by the controller only for the purpose for which they were received. The body obligated to secrecy must give its consent to any transfer to a private body.

(2) The data may be processed or used for another purpose only if the change of purpose is permitted by special legislation.

Section 40 Processing and use of personal data by research institutions

(1) Personal data collected or recorded for purposes of scientific research may be processed or used only for purposes of scientific research.

(2) Personal data shall be rendered anonymous as soon as the research purpose allows. Until then, the features enabling the attribution of information concerning personal or material circumstances to an identified or identifiable person shall be kept separately. They may be combined with the information only to the extent required by the research purpose.

(3) Bodies conducting scientific research may publish personal data only if

1. the data subject has consented,
2. this is essential to present research findings concerning events of contemporary history.

Section 41 Collection, processing and use of personal data by the media

(1) In their legislation, the *Länder* shall ensure that regulations corresponding to the provisions of Sections 5, 9 and 38a, including rules on liability in accordance with Section 7, apply to the collection, processing and use of personal data by media enterprises and auxiliary media enterprises exclusively for their own journalistic, editorial or literary purposes.

(2) If the journalistic-editorial collection, processing or use of personal data by *Deutsche Welle* leads to the publication of counter-statements by the data subject, these counter-statements shall be added to the recorded data and retained for the same length of time as the data themselves.

(3) If reporting by *Deutsche Welle* infringes the privacy of an individual, this person may request information about the recorded data relating to him/her on which the reporting was based. This information may be refused after considering the legitimate interests of the parties concerned, where

1. the data allow the identification of persons who are or were professionally involved as journalists in preparing, producing or disseminating broadcasts,
2. the data allow the identification of the supplier or source of contributions, documents and communications for the editorial part,
3. disclosure of the data obtained by research or other means would compromise *Deutsche Welle*'s journalistic duty by divulging its information resources.

The data subject may request that inaccurate data be corrected.

(4) In all other respects, Sections 5, 7, 9 and 38a shall apply to *Deutsche Welle*. Section 42 shall apply in place of Sections 24 through 26, even where administrative matters are concerned.

Section 42 Data protection official of *Deutsche Welle*

(1) *Deutsche Welle* shall appoint a data protection official who shall take the place of the Federal Commissioner for Data Protection and Information Freedom. The data protection official shall be nominated by the director-general and appointed by the administrative board for a term of four years; the appointment may be renewed. The office of data protection official may be held in combination with other duties within the broadcaster.

(2) The data protection official shall monitor compliance with this Act and other data protection provisions. He or she shall be independent in performing the duties of this office and subject only to the law. In all other respects, he or she shall be subject to the administrative and legal supervision of the administrative board.

(3) Anyone may appeal to the data protection official in accordance with Section 21 first sentence.

(4) The data protection official of *Deutsche Welle* shall submit an activity report to the organs of *Deutsche Welle* every two years, starting on 1 January 1994. In addition, the data protection official shall submit special reports at the decision of an organ of *Deutsche Welle*. The data protection official shall provide a copy of his or her activity reports to the Federal Commissioner for Data Protection and Freedom of Information as well.

(5) *Deutsche Welle* shall make further arrangements for its area of activity in accordance with Sections 23 through 26 of the Act. Sections 4f and 4g shall remain unaffected.

Section 42a Obligation to notify in case of unlawful access to data

If a private body as defined in Section 2 (4) or a public body as referred to in Section 27 (1) first sentence no. 2 determines that

1. special categories of personal data (Section 3 (9)),
2. personal data subject to professional secrecy,

3. personal data referring to criminal or administrative offences or to suspected criminal or administrative offences, or
4. personal data concerning bank or credit card accounts

it has recorded have been unlawfully transferred or otherwise unlawfully disclosed to third parties, threatening serious harm to the rights or legitimate interests of data subjects, then the private body shall notify the competent supervisory and the data subjects without delay in accordance with the second through fifth sentences. Data subjects shall be informed as soon as appropriate measures to safeguard the data have been taken and notification would no longer endanger criminal prosecution. The notification of data subjects shall describe the nature of the unlawful disclosure and recommend measures to minimize possible harm. The notification of the competent supervisory authority shall in addition describe possible harmful consequences of the unlawful disclosure and measures taken by the body as a result. Where notifying the data subjects would require a disproportionate effort, in particular due to the large number of persons affected, such notification may be replaced by public advertisements of at least one-half page in at least two national daily newspapers, or by another equally effective measure for notifying data subjects. Notification distributed by the body required to provide notification may be used against that body in criminal proceedings or proceedings under the Administrative Offences Act, or against an associate of the body required to provide notification as defined in Section 52 (1) of the Code of Criminal Procedure only with the consent of the body required to provide notification.

Part V

Final provisions

Section 43 Administrative offences

(1) An administrative offence shall be deemed to have been committed by anyone who, whether intentionally or through negligence

1. in violation of Section 4d (1), also in conjunction with Section 4e second sentence, fails to notify, fails to do so within the prescribed time limit or fails to provide complete information,
2. in violation of Section 4f (1) first or second sentence, in each case also in conjunction with the third and sixth sentences, fails to appoint a data protection official or fails to do so within the prescribed time limit or in the prescribed manner,

2 a. in violation of Section 10 (4) third sentence fails to ensure that the transfer of data can be ascertained and checked,

2 b. in violation of Section 11 (2) second sentence fails to assign work correctly, completely or in accordance with the rules, or in violation of Section 11 (2) fourth sentence fails to verify compliance with the technical and organizational measures taken by the processor before data processing begins,

3. in violation of Section 28 (4) second sentence fails to notify the data subject, or fails to do so correctly or within the prescribed time limit, or fails to ensure that the data subject may obtain the relevant information,

3 a. in violation of Section 28 (4) fourth sentence requires a stricter form,

4. in violation of Section 28 (5) second sentence transfers or uses personal data,

4 a. in violation of Section 28a (3) first sentence fails to notify, or fails to do so correctly, completely or within the prescribed time limit,[19]

5. in violation of Section 29 (2) third or fourth sentence fails to record the evidence described there or the means of presenting it in a credible way,

6. in violation of Section 29 (3) first sentence, incorporates personal data into electronic or printed address, telephone, business or similar directories,

7. in violation of Section 29 (3) second sentence fails to ensure the inclusion of identifiers,

7 a. in violation of Section 29 (6) fails to treat a request for information properly,[20]

7 b. in violation of Section 29 (7) first sentence fails to notify a consumer, or fails to do so correctly, completely or within the prescribed time limit,[21]

8. in violation of Section 33 (1) fails to notify the data subject, or fails to do so correctly or completely,

8 a. in violation of Section 34 (1) first sentence, also in conjunction with the third sentence, in violation of Section 34 (1a), in violation of Section 34 (2) first sentence, also in conjunction with the second sentence, or in violation of Section 34 (2) fifth sentence, (3) first sentence or second sentence or (4) first sentence, also in conjunction with the second sentence, fails to provide information, or fails to do so correctly, completely or within the prescribed time limit, or in violation of Section 34 (1a) fails to record data,[22]

8 b. in violation of Section 34 (2) third sentence fails to provide information, or fails to do so correctly, completely or within the prescribed time limit,[23]

8 c. in violation of Section 34 (2) fourth sentence fails to provide the data subject with the information referred to, or fails to do so within the prescribed time limit,[24]

9. in violation of Section 35 (6) third sentence transfers data without providing the counter-statement,

10. in violation of Section 38 (3) first sentence or (4) first sentence fails to provide information, or fails to do so correctly, completely or within the prescribed time limit, or fails to allow a measure, or

---

[19] In force from 1 April 2010.

[20] In force from 11 June 2010.

[21] In force from 11 June 2010.

[22] In force from 1 April 2010.

[23] In force from 1 April 2010.

[24] In force from 1 April 2010.

11. fails to comply with an executable instruction under Section 38 (5) first sentence.

(2) An administrative offence shall be deemed to have been committed by anyone who, whether intentionally or through negligence

1. collects or processes personal data which are not generally accessible without authorization,
2. makes available personal data which are not generally accessible by means of automated retrieval without authorization,
3. retrieves personal data which are not generally accessible without authorization, or obtains such data for themselves or others from automated processing operations or non-automated files without authorization,
4. obtains transfer of personal data which are not generally accessible by providing false information,
5. in violation of Section 16 (4) first sentence, Section 28 (5) first sentence, also in conjunction with Section 29 (4), Section 39 (1) first sentence or Section 40 (1), uses transferred data for other purposes,

5 a. in violation of Section 28 (3b) makes the conclusion of a contract dependent on the consent of the data subject,

5 b. in violation of Section 28 (4) first sentence processes or uses data for purposes of advertising or market or opinion research,

6. in violation of Section 30 (1) second sentence, Section 30a (3) third sentence or Section 40 (2) third sentence combines a feature referred to there with specific information, or
7. in violation of Section 42a first sentence, fails to notify or fails to do so correctly, completely or within the prescribed time limit.

(3) Administrative offences may be punished by a fine of up to € 50,000 in the case of subsection 1, and a fine of up to € 300,000 in the cases of subsection 2. The fine should exceed the financial benefit to the perpetrator derived from the administrative offence. If the amounts mentioned in the first sentence are not sufficient to do so, they may be increased.

Section 44 Criminal offences

(1) Anyone who wilfully commits an offence described in Section 43 (2) in exchange for payment or with the intention of enriching him-/herself or another person, or of harming another person shall be liable to imprisonment for up to two years or to a fine.

(2) Such offences shall be prosecuted only if a complaint is filed. Complaints may be filed by the data subject, the controller, the Federal Commissioner for Data Protection and Freedom of Information and the supervisory authority.

Part VI

Transitional provisions

Section 45 Current applications

The collection, processing or use of personal data already under way on 23 May 2001 shall be brought into compliance with the provisions of this Act within three years of that date. Where provisions of this Act are applied in legal provisions outside the scope of Directive 95/46/EC of the European Parliament and the Council of 24 October 1995 on the protection of individuals with regard to the processing of personal data and on the free movement of such data, the collection, processing or use of personal data already under way on 23 May 2001 shall be brought into compliance with the provisions of this Act within five years of that date.

Section 46 Continued validity of definitions

(1) Where used in special federal law, the term “filing system” shall mean

1. a collection of personal data which can be evaluated with reference to specific features using automated procedures (automated filing system), or
2. any other collection of personal data which is similarly structured and which can be arranged, re-arranged and evaluated according to specific characteristics (non-automated filing system).

This shall not include files and sets of files unless they can be rearranged and evaluated using automated procedures.

(2) Where used in special federal law, the term “file” shall mean any document serving official purposes to which the definition of a filing system under subsection 1 does not apply; this shall include image and sound recording media. It shall not include preliminary drafts and notes that are not intended to be part of an operation.

(3) Where used in special federal law, the term “recipient” shall mean any person or body other than the controller. Recipients shall not mean the data subject or persons or bodies collecting, processing or using personal data in Germany, in another European Union Member State or another state party to the Agreement on the European Economic Area on behalf of another.

Section 47 Transitional provision

For the processing and use of data collected or recorded prior to 1 September 2009, Section 28 in the version in force until that date shall continue to apply

1. to purposes of market or opinion research until 31 August 2010,
2. to advertising purposes until 31 August 2012.

Section 28 in the version previously in force

Section 28 Storage, communication and use of data for own purposes

(1) The collection, storage, modification or transfer of personal data or their use as a means of fulfilling one’s own business purposes shall be admissible

1. in accordance with the purposes of a contract or a quasi-contractual fiduciary relationship with the data subject,

2. in so far as this is necessary to safeguard justified interests of the controller of the filing system and there is no reason to assume that the data subject has an overriding legitimate interest in his data being excluded from processing or use,
3. if the data is generally accessible or the controller of the filing system would be entitled to publish them, unless the data subject's legitimate interest in his data being excluded from processing or use clearly outweighs the justified interest of the controller of the filing system.

In connection with the collection of personal data, the purposes for which the data are to be processed or used are to be stipulated in concrete terms.

(2) Transfer or use for another purpose shall be admissible only if the requirements of Section 1, sentence 1, Nos. 2 and 3 are met.

(3) Transfer or use for another purpose shall also be admissible:

1. in so far as it is necessary to protect the justified interests of a third party or
2. to avert threats to the state security and public safety and to prosecute criminal offences or
3. for purposes of advertising, market and opinion research if the data, compiled in lists or otherwise combined, concern members of a group of persons and are restricted to
   a) the data subject’s membership of this group of persons,
   b) occupation or type of business,
   c) name,
   d) title,
   e) academic degrees,
   f) address and
   g) year of birth

   and if there is no reason to assume that the data subject has a legitimate interest in his data being excluded from transfer, or
4. if this is necessary in the interest of a research institute for the conduct of scientific research, if scientific interest in conduct of the research project substantially outweighs the interest of the data subject in excluding the change of purpose and if the research purpose cannot be attained by other means or can be attained thus only with disproportionate effort.

In the cases referred to in the first sentence of No. 3, it is to be assumed that such interest exists where data are to be transferred which were stored for the purposes of a contract or a quasi-contractual fiduciary relationship and which concern

1. criminal offences,
2. administrative offences and,
3. when transferred by the employer, to the legal status under labour law.

(4) If the data subject objects vis-à-vis the controller of the filing system to the use or transfer of his data for purposes of advertising or of market opinion research, use or transfer for such purposes shall be inadmissible. In approaching the data subject for the purpose of advertising or market or opinion research, the data subject shall be informed of the identity of the controller and the right of objections in accordance with sentence 1 above; in so far as the party approaching the data subject uses personal data of the latter which are stored by a body which is unknown to him, he shall also ensure that the data subject is able to obtain information on the origin of the data. If the data subject lodges an objection to the processing or use of the data for the purpose of advertising or market or opinion research with the third party to whom the data are transferred pursuant to sub-section 3, the latter shall block the data for these purposes.

(5) The third party to whom the data have been transferred may process or use the transferred data only for the purpose for which they were transferred to him. Processing or use for other purposes shall be admissible for private bodies only if the requirements of sub-sections 1 and 2 above are met and for public bodies only if the requirements of Section 14 (2) are met. The transferring body shall point this out to the third party.

(6) The collection, processing and use of special types of personal data (Section 3 (9)) for own business purposes shall be admissible when the data subject has not consented in accordance with Section 4a (3) if

1. this is necessary in order to protect vital interests of the data subject or of a third party, in so far as the data subject is unable to give his consent for physical or legal reasons,
2. the data concerned has evidently been made public by the data subject,
3. this is necessary in order to assert, exercise or defend legal claims and there is no reason to assume that the data subject has an overriding legitimate interest in excluding such collection, processing or use, or
4. this is necessary for the purposes of scientific research, where the scientific interest in carrying out the research project substantially outweighs the data subject's interest in excluding collection, processing and use and the purpose of the research cannot be achieved in any other way or would otherwise necessitate disproportionate effort.

(7) The collection of special types of personal data (Section 3 (9)) shall further be admissible if this is necessary for the purposes of preventive medicine, medical diagnosis, health care or treatment or the administration of health services and the processing of these data is carried out by medical personnel or other persons who are subject to an obligation to maintain secrecy. The processing and use of data for the purposes stated in sentence 1 shall be subject to the obligations to maintain secrecy which apply to the persons stated in sentence 1. The collection, processing or use of data on the health of persons by members of a profession other than those stipulated in Section 203 (1) and (3) of the Penal Code, the exercising of which profession involves determining, curing or alleviating illnesses or producing or selling aids shall be admissible only under those conditions according to which a doctor would also be authorised for these purposes.

(8) Special types of personal data (Section 3 (9)) may be transferred or used only if the requirements of sub-section 6, Nos. 1 to 4 or the first sentence of sub-section 7 are met. Transfer or use shall also be admissible if necessary to avert substantial threats to state security or public safety and to prosecute major criminal offences.

(9) Organisations of a political, philosophical or religious nature and trade union organisations may collect, process or use special types of personal data (Section 3 (9)) in so far as this is necessary for the organisation's activities. This shall apply only to personal data of their members or of persons who maintain regular contact with the organisations in connection with the purposes of their activities. The transfer of these personal data to persons or bodies outside of the organisation concerned shall be admissible only if the requirements of Section 4a (3) are met. Sub-section 3, No. 2 shall apply mutatis mutandis.

Section 48 Report of the Federal Government

The Federal Government shall report to the German Bundestag

1. by 31 December 2012 on the impacts of Sections 30a and 42a,
2. by 31 December 2014 on the impacts of the amendments to Sections 28 and 29.

If the Federal Government is of the view that legislative measures are advisable, the report shall contain a recommendation.

Annex (to Section 9, first sentence)

Where personal data are processed or used in automated form, the internal organization of authorities or enterprises is to be such that it meets the specific requirements of data protection. In particular, measures suited to the type of personal data or categories of data to be protected shall be taken

1. to prevent unauthorized persons from gaining access to data processing systems for processing or using personal data (access control),
2. to prevent data processing systems from being used without authorization (access control),
3. to ensure that persons authorized to use a data processing system have access only to those data they are authorized to access, and that personal data cannot be read, copied, altered or removed without authorization during processing, use and after recording (access control),
4. to ensure that personal data cannot be read, copied, altered or removed without authorization during electronic transfer or transport or while being recorded onto data storage media, and that it is possible to ascertain and check which bodies are to be transferred personal data using data transmission facilities (disclosure control),
5. to ensure that it is possible after the fact to check and ascertain whether personal data have been entered into, altered or removed from data processing systems and if so, by whom (input control),
6. to ensure that personal data processed on behalf of others are processed strictly in compliance with the controller’s instructions (job control),
7. to ensure that personal data are protected against accidental destruction or loss (availability control),
8. to ensure that data collected for different purposes can be processed separately.

One measure in accordance with the second sentence Nos. 2 to 4 is in particular the use of the latest encryption procedures.
