# Supplementary material for: Intracranial Ependymoma: Long-Term Results in a Series of 21 Patients Treated with Stereotactic 125Iodine Brachytherapy
Source: PLoS One. 2012 Nov 5;7(11):e47266. doi: 10.1371/journal.pone.0047266 (PMC3489891; doi:10.1371/journal.pone.0047266)
Supplement: File S3 — Data Protection Legislation. (PDF) [file pone.0047266.s005.pdf]

Bundesdatenschutzgesetz (BDSG)

BDSG

Ausfertigungsdatum: 20.12.1990

Vollzitat:

"Bundesdatenschutzgesetz in der Fassung der Bekanntmachung vom 14. Januar 2003 (BGBl. I S. 66), das zuletzt durch Artikel 1 des Gesetzes vom 14. August 2009 (BGBl. I S. 2814) geändert worden ist"

**Stand:**	Neugefasst durch Bek. v. 14.1.2003 I 66;<br>Zuletzt geändert durch Art. 1 G v. 14.8.2009 I 2814

Fußnote

```
(+++ Textnachweis ab: 1.6.1991 +++)
```

Das G wurde als Art. 1 des G v. 20.12.1990 I 2954 vom Bundestag mit Zustimmung des Bundesrates beschlossen; § 10 Abs. 4 Satz 3 und 4 ist am ersten Tage des vierundzwanzigsten auf die Verkündung folgenden Kalendermonats, im übrigen am ersten Tage des sechsten auf die Verkündung folgenden Kalendermonats gem. Art. 6 Abs. 2 Satz 1 u. 2 G v. 20.12.1990 I 2954 in Kraft getreten. Das G wurde am 29.12.1990 verkündet.

Inhaltsübersicht

Erster Abschnitt
Allgemeine und gemeinsame Bestimmungen

§ 1	Zweck und Anwendungsbereich des Gesetzes
§ 2	Öffentliche und nicht-öffentliche Stellen
§ 3	Weitere Begriffsbestimmungen
§ 3a	Datenvermeidung und Datensparsamkeit
§ 4	Zulässigkeit der Datenerhebung, -verarbeitung und -nutzung
§ 4a	Einwilligung
§ 4b	Übermittlung personenbezogener Daten ins Ausland sowie an über- und zwischenstaatliche Stellen
§ 4c	Ausnahmen
§ 4d	Meldepflicht
§ 4e	Inhalt der Meldepflicht
§ 4f	Beauftragter für den Datenschutz
§ 4g	Aufgaben des Beauftragten für den Datenschutz
§ 5	Datengeheimnis
§ 6	Rechte des Betroffenen
§ 6a	Automatisierte Einzelentscheidung
§ 6b	Beobachtung öffentlich zugänglicher Räume mit optisch-elektronischen Einrichtungen
§ 6c	Mobile personenbezogene Speicher- und Verarbeitungsmedien
§ 7	Schadensersatz
§ 8	Schadensersatz bei automatisierter Datenverarbeitung durch öffentliche Stellen
§ 9	Technische und organisatorische Maßnahmen
§ 9a	Datenschutzaudit
§ 10	Einrichtung automatisierter Abrufverfahren
§ 11	Erhebung, Verarbeitung oder Nutzung personenbezogener Daten im Auftrag

Zweiter Abschnitt
Datenverarbeitung der öffentlichen Stellen

Erster Unterabschnitt
Rechtsgrundlagen der Datenverarbeitung
§ 12 Anwendungsbereich
§ 13 Datenerhebung
§ 14 Datenspeicherung, -veränderung und -nutzung
§ 15 Datenübermittlung an öffentliche Stellen
§ 16 Datenübermittlung an nicht-öffentliche Stellen
§ 17 (weggefallen)
§ 18 Durchführung des Datenschutzes in der Bundesverwaltung
Zweiter Unterabschnitt
Rechte des Betroffenen
§ 19 Auskunft an den Betroffenen
§ 19a Benachrichtigung
§ 20 Berichtigung, Löschung und Sperrung von Daten; Widerspruchsrecht
§ 21 Anrufung des Bundesbeauftragten für den Datenschutz und die Informationsfreiheit
Dritter Unterabschnitt
Bundesbeauftragter für den Datenschutz und die Informationsfreiheit
§ 22 Wahl des Bundesbeauftragten für den Datenschutz und die Informationsfreiheit
§ 23 Rechtsstellung des Bundesbeauftragten für den Datenschutz und die Informationsfreiheit
§ 24 Kontrolle durch den Bundesbeauftragten für den Datenschutz und die Informationsfreiheit
§ 25 Beanstandungen durch den Bundesbeauftragten für den Datenschutz und die Informationsfreiheit
§ 26 Weitere Aufgaben des Bundesbeauftragten für den Datenschutz und die Informationsfreiheit
Dritter Abschnitt
Datenverarbeitung nicht-öffentlicher Stellen und öffentlich-rechtlicher Wettbewerbsunternehmen
Erster Unterabschnitt
Rechtsgrundlagen der Datenverarbeitung
§ 27 Anwendungsbereich
§ 28 Datenerhebung und -speicherung für eigene Geschäftszwecke
§ 28a Datenübermittlung an Auskunfteien
§ 28b Scoring
§ 29 Geschäftsmäßige Datenerhebung und -speicherung zum Zweck der Übermittlung
§ 30 Geschäftsmäßige Datenerhebung und -speicherung zum Zweck der Übermittlung in anonymisierter Form
§ 30a Geschäftsmäßige Datenerhebung und -speicherung für Zwecke der Markt- oder Meinungsforschung
§ 31 Besondere Zweckbindung
§ 32 Datenerhebung, -verarbeitung und -nutzung für Zwecke des Beschäftigungsverhältnisses
Zweiter Unterabschnitt
Rechte des Betroffenen
§ 33 Benachrichtigung des Betroffenen
§ 34 Auskunft an den Betroffenen
§ 35 Berichtigung, Löschung und Sperrung von Daten
Dritter Unterabschnitt
Aufsichtsbehörde
§§ 36 und 37 (weggefallen)
§ 38 Aufsichtsbehörde
§ 38a Verhaltensregeln zur Förderung der Durchführung datenschutzrechtlicher Regelungen
Vierter Abschnitt
Sondervorschriften
§ 39 Zweckbindung bei personenbezogenen Daten, die einem Berufs- oder besonderen Amtsgeheimnis unterliegen
§ 40 Verarbeitung und Nutzung personenbezogener Daten durch Forschungseinrichtungen

§ 41 Erhebung, Verarbeitung und Nutzung personenbezogener Daten durch die Medien
§ 42 Datenschutzbeauftragter der Deutschen Welle
§ 42a Informationspflicht bei unrechtmäßiger Kenntniserlangung von Daten
Fünfter Abschnitt
Schlussvorschriften
§ 43 Bußgeldvorschriften
§ 44 Strafvorschriften
Sechster Abschnitt
Übergangsvorschriften
§ 45 Laufende Verwendungen
§ 46 Weitergeltung von Begriffsbestimmungen
§ 47 Übergangsregelung
§ 48 Bericht der Bundesregierung
Anlage (zu § 9 Satz 1)

Erster Abschnitt
Allgemeine und gemeinsame Bestimmungen

§ 1 Zweck und Anwendungsbereich des Gesetzes

(1) Zweck dieses Gesetzes ist es, den Einzelnen davor zu schützen, dass er durch den Umgang mit seinen personenbezogenen Daten in seinem Persönlichkeitsrecht beeinträchtigt wird.

(2) Dieses Gesetz gilt für die Erhebung, Verarbeitung und Nutzung personenbezogener Daten durch

1. öffentliche Stellen des Bundes,
2. öffentliche Stellen der Länder, soweit der Datenschutz nicht durch Landesgesetz geregelt ist und soweit sie
   a) Bundesrecht ausführen oder
   b) als Organe der Rechtspflege tätig werden und es sich nicht um Verwaltungsangelegenheiten handelt,
3. nicht-öffentliche Stellen, soweit sie die Daten unter Einsatz von Datenverarbeitungsanlagen verarbeiten, nutzen oder dafür erheben oder die Daten in oder aus nicht automatisierten Dateien verarbeiten, nutzen oder dafür erheben, es sei denn, die Erhebung, Verarbeitung oder Nutzung der Daten erfolgt ausschließlich für persönliche oder familiäre Tätigkeiten.

(3) Soweit andere Rechtsvorschriften des Bundes auf personenbezogene Daten einschließlich deren Veröffentlichung anzuwenden sind, gehen sie den Vorschriften dieses Gesetzes vor. Die Verpflichtung zur Wahrung gesetzlicher Geheimhaltungspflichten oder von Berufs- oder besonderen Amtsgeheimnissen, die nicht auf gesetzlichen Vorschriften beruhen, bleibt unberührt.

(4) Die Vorschriften dieses Gesetzes gehen denen des Verwaltungsverfahrensgesetzes vor, soweit bei der Ermittlung des Sachverhalts personenbezogene Daten verarbeitet werden.

(5) Dieses Gesetz findet keine Anwendung, sofern eine in einem anderen Mitgliedstaat der Europäischen Union oder in einem anderen Vertragsstaat des Abkommens über den Europäischen Wirtschaftsraum belegene verantwortliche Stelle personenbezogene Daten im Inland erhebt, verarbeitet oder nutzt, es sei denn, dies erfolgt durch eine Niederlassung im Inland. Dieses Gesetz findet Anwendung, sofern eine verantwortliche Stelle, die nicht in einem Mitgliedstaat der Europäischen Union oder in einem anderen Vertragsstaat des Abkommens über den Europäischen Wirtschaftsraum belegen ist, personenbezogene Daten im Inland erhebt, verarbeitet oder nutzt. Soweit die verantwortliche Stelle nach diesem Gesetz zu nennen ist, sind auch Angaben über im Inland ansässige Vertreter zu machen. Die Sätze 2 und 3 gelten nicht, sofern Datenträger nur zum Zweck des Transits durch das Inland eingesetzt werden. § 38 Abs. 1 Satz 1 bleibt unberührt.

§ 2 Öffentliche und nicht-öffentliche Stellen

(1) Öffentliche Stellen des Bundes sind die Behörden, die Organe der Rechtspflege und andere öffentlich-rechtlich organisierte Einrichtungen des Bundes, der bundesunmittelbaren Körperschaften, Anstalten und Stiftungen des öffentlichen Rechts sowie deren Vereinigungen ungeachtet ihrer Rechtsform. Als öffentliche Stellen gelten die aus

dem Sondervermögen Deutsche Bundespost durch Gesetz hervorgegangenen Unternehmen, solange ihnen ein ausschließliches Recht nach dem Postgesetz zusteht.

(2) Öffentliche Stellen der Länder sind die Behörden, die Organe der Rechtspflege und andere öffentlich-rechtlich organisierte Einrichtungen eines Landes, einer Gemeinde, eines Gemeindeverbandes und sonstiger der Aufsicht des Landes unterstehender juristischer Personen des öffentlichen Rechts sowie deren Vereinigungen ungeachtet ihrer Rechtsform.

(3) Vereinigungen des privaten Rechts von öffentlichen Stellen des Bundes und der Länder, die Aufgaben der öffentlichen Verwaltung wahrnehmen, gelten ungeachtet der Beteiligung nicht-öffentlicher Stellen als öffentliche Stellen des Bundes, wenn

1. sie über den Bereich eines Landes hinaus tätig werden oder
2. dem Bund die absolute Mehrheit der Anteile gehört oder die absolute Mehrheit der Stimmen zusteht.

Andernfalls gelten sie als öffentliche Stellen der Länder.

(4) Nicht-öffentliche Stellen sind natürliche und juristische Personen, Gesellschaften und andere Personenvereinigungen des privaten Rechts, soweit sie nicht unter die Absätze 1 bis 3 fallen. Nimmt eine nicht-öffentliche Stelle hoheitliche Aufgaben der öffentlichen Verwaltung wahr, ist sie insoweit öffentliche Stelle im Sinne dieses Gesetzes.

§ 3 Weitere Begriffsbestimmungen

(1) Personenbezogene Daten sind Einzelangaben über persönliche oder sachliche Verhältnisse einer bestimmten oder bestimmbaren natürlichen Person (Betroffener).

(2) Automatisierte Verarbeitung ist die Erhebung, Verarbeitung oder Nutzung personenbezogener Daten unter Einsatz von Datenverarbeitungsanlagen. Eine nicht automatisierte Datei ist jede nicht automatisierte Sammlung personenbezogener Daten, die gleichartig aufgebaut ist und nach bestimmten Merkmalen zugänglich ist und ausgewertet werden kann.

(3) Erheben ist das Beschaffen von Daten über den Betroffenen.

(4) Verarbeiten ist das Speichern, Verändern, Übermitteln, Sperren und Löschen personenbezogener Daten. Im Einzelnen ist, ungeachtet der dabei angewendeten Verfahren:

1. Speichern das Erfassen, Aufnehmen oder Aufbewahren personenbezogener Daten auf einem Datenträger zum Zweck ihrer weiteren Verarbeitung oder Nutzung,
2. Verändern das inhaltliche Umgestalten gespeicherter personenbezogener Daten,
3. Übermitteln das Bekanntgeben gespeicherter oder durch Datenverarbeitung gewonnener personenbezogener Daten an einen Dritten in der Weise, dass
   a) die Daten an den Dritten weitergegeben werden oder
   b) der Dritte zur Einsicht oder zum Abruf bereitgehaltene Daten einsieht oder abruft,
4. Sperren das Kennzeichnen gespeicherter personenbezogener Daten, um ihre weitere Verarbeitung oder Nutzung einzuschränken,
5. Löschen das Unkenntlichmachen gespeicherter personenbezogener Daten.

(5) Nutzen ist jede Verwendung personenbezogener Daten, soweit es sich nicht um Verarbeitung handelt.

(6) Anonymisieren ist das Verändern personenbezogener Daten derart, dass die Einzelangaben über persönliche oder sachliche Verhältnisse nicht mehr oder nur mit einem unverhältnismäßig großen Aufwand an Zeit, Kosten und Arbeitskraft einer bestimmten oder bestimmbaren natürlichen Person zugeordnet werden können.

(6a) Pseudonymisieren ist das Ersetzen des Namens und anderer Identifikationsmerkmale durch ein Kennzeichen zu dem Zweck, die Bestimmung des Betroffenen auszuschließen oder wesentlich zu erschweren.

(7) Verantwortliche Stelle ist jede Person oder Stelle, die personenbezogene Daten für sich selbst erhebt, verarbeitet oder nutzt oder dies durch andere im Auftrag vornehmen lässt.

(8) Empfänger ist jede Person oder Stelle, die Daten erhält. Dritter ist jede Person oder Stelle außerhalb der verantwortlichen Stelle. Dritte sind nicht der Betroffene sowie Personen und Stellen, die im Inland, in einem

anderen Mitgliedstaat der Europäischen Union oder in einem anderen Vertragsstaat des Abkommens über den Europäischen Wirtschaftsraum personenbezogene Daten im Auftrag erheben, verarbeiten oder nutzen.

(9) Besondere Arten personenbezogener Daten sind Angaben über die rassische und ethnische Herkunft, politische Meinungen, religiöse oder philosophische Überzeugungen, Gewerkschaftszugehörigkeit, Gesundheit oder Sexualleben.

(10) Mobile personenbezogene Speicher- und Verarbeitungsmedien sind Datenträger,

1. die an den Betroffenen ausgegeben werden,
2. auf denen personenbezogene Daten über die Speicherung hinaus durch die ausgebende oder eine andere Stelle automatisiert verarbeitet werden können und
3. bei denen der Betroffene diese Verarbeitung nur durch den Gebrauch des Mediums beeinflussen kann.

(11) Beschäftigte sind:

1. Arbeitnehmerinnen und Arbeitnehmer,
2. zu ihrer Berufsbildung Beschäftigte,
3. Teilnehmerinnen und Teilnehmer an Leistungen zur Teilhabe am Arbeitsleben sowie an Abklärungen der beruflichen Eignung oder Arbeitserprobung (Rehabilitandinnen und Rehabilitanden),
4. in anerkannten Werkstätten für behinderte Menschen Beschäftigte,
5. nach dem Jugendfreiwilligendienstegesetz Beschäftigte,
6. Personen, die wegen ihrer wirtschaftlichen Unselbständigkeit als arbeitnehmerähnliche Personen anzusehen sind; zu diesen gehören auch die in Heimarbeit Beschäftigten und die ihnen Gleichgestellten,
7. Bewerberinnen und Bewerber für ein Beschäftigungsverhältnis sowie Personen, deren Beschäftigungsverhältnis beendet ist,
8. Beamtinnen, Beamte, Richterinnen und Richter des Bundes, Soldatinnen und Soldaten sowie Zivildienstleistende.

§ 3a Datenvermeidung und Datensparsamkeit

Die Erhebung, Verarbeitung und Nutzung personenbezogener Daten und die Auswahl und Gestaltung von Datenverarbeitungssystemen sind an dem Ziel auszurichten, so wenig personenbezogene Daten wie möglich zu erheben, zu verarbeiten oder zu nutzen. Insbesondere sind personenbezogene Daten zu anonymisieren oder zu pseudonymisieren, soweit dies nach dem Verwendungszweck möglich ist und keinen im Verhältnis zu dem angestrebten Schutzzweck unverhältnismäßigen Aufwand erfordert.

§ 4 Zulässigkeit der Datenerhebung, -verarbeitung und -nutzung

(1) Die Erhebung, Verarbeitung und Nutzung personenbezogener Daten sind nur zulässig, soweit dieses Gesetz oder eine andere Rechtsvorschrift dies erlaubt oder anordnet oder der Betroffene eingewilligt hat.

(2) Personenbezogene Daten sind beim Betroffenen zu erheben. Ohne seine Mitwirkung dürfen sie nur erhoben werden, wenn

1. eine Rechtsvorschrift dies vorsieht oder zwingend voraussetzt oder
2.
   a) die zu erfüllende Verwaltungsaufgabe ihrer Art nach oder der Geschäftszweck eine Erhebung bei anderen Personen oder Stellen erforderlich macht oder
   b) die Erhebung beim Betroffenen einen unverhältnismäßigen Aufwand erfordern würde

   und keine Anhaltspunkte dafür bestehen, dass überwiegende schutzwürdige Interessen des Betroffenen beeinträchtigt werden.

(3) Werden personenbezogene Daten beim Betroffenen erhoben, so ist er, sofern er nicht bereits auf andere Weise Kenntnis erlangt hat, von der verantwortlichen Stelle über

1. die Identität der verantwortlichen Stelle,
2. die Zweckbestimmungen der Erhebung, Verarbeitung oder Nutzung und

3. die Kategorien von Empfängern nur, soweit der Betroffene nach den Umständen des Einzelfalles nicht mit der Übermittlung an diese rechnen muss,

zu unterrichten. Werden personenbezogene Daten beim Betroffenen aufgrund einer Rechtsvorschrift erhoben, die zur Auskunft verpflichtet, oder ist die Erteilung der Auskunft Voraussetzung für die Gewährung von Rechtsvorteilen, so ist der Betroffene hierauf, sonst auf die Freiwilligkeit seiner Angaben hinzuweisen. Soweit nach den Umständen des Einzelfalles erforderlich oder auf Verlangen, ist er über die Rechtsvorschrift und über die Folgen der Verweigerung von Angaben aufzuklären.

§ 4a Einwilligung

(1) Die Einwilligung ist nur wirksam, wenn sie auf der freien Entscheidung des Betroffenen beruht. Er ist auf den vorgesehenen Zweck der Erhebung, Verarbeitung oder Nutzung sowie, soweit nach den Umständen des Einzelfalles erforderlich oder auf Verlangen, auf die Folgen der Verweigerung der Einwilligung hinzuweisen. Die Einwilligung bedarf der Schriftform, soweit nicht wegen besonderer Umstände eine andere Form angemessen ist. Soll die Einwilligung zusammen mit anderen Erklärungen schriftlich erteilt werden, ist sie besonders hervorzuheben.

(2) Im Bereich der wissenschaftlichen Forschung liegt ein besonderer Umstand im Sinne von Absatz 1 Satz 3 auch dann vor, wenn durch die Schriftform der bestimmte Forschungszweck erheblich beeinträchtigt würde. In diesem Fall sind der Hinweis nach Absatz 1 Satz 2 und die Gründe, aus denen sich die erhebliche Beeinträchtigung des bestimmten Forschungszwecks ergibt, schriftlich festzuhalten.

(3) Soweit besondere Arten personenbezogener Daten (§ 3 Abs. 9) erhoben, verarbeitet oder genutzt werden, muss sich die Einwilligung darüber hinaus ausdrücklich auf diese Daten beziehen.

§ 4b Übermittlung personenbezogener Daten ins Ausland sowie an über- oder zwischenstaatliche Stellen

(1) Für die Übermittlung personenbezogener Daten an Stellen

1. in anderen Mitgliedstaaten der Europäischen Union,
2. in anderen Vertragsstaaten des Abkommens über den Europäischen Wirtschaftsraum oder
3. der Organe und Einrichtungen der Europäischen Gemeinschaften

gelten § 15 Abs. 1, § 16 Abs. 1 und §§ 28 bis 30a nach Maßgabe der für diese Übermittlung geltenden Gesetze und Vereinbarungen, soweit die Übermittlung im Rahmen von Tätigkeiten erfolgt, die ganz oder teilweise in den Anwendungsbereich des Rechts der Europäischen Gemeinschaften fallen.

(2) Für die Übermittlung personenbezogener Daten an Stellen nach Absatz 1, die nicht im Rahmen von Tätigkeiten erfolgt, die ganz oder teilweise in den Anwendungsbereich des Rechts der Europäischen Gemeinschaften fallen, sowie an sonstige ausländische oder über- oder zwischenstaatliche Stellen gilt Absatz 1 entsprechend. Die Übermittlung unterbleibt, soweit der Betroffene ein schutzwürdiges Interesse an dem Ausschluss der Übermittlung hat, insbesondere wenn bei den in Satz 1 genannten Stellen ein angemessenes Datenschutzniveau nicht gewährleistet ist. Satz 2 gilt nicht, wenn die Übermittlung zur Erfüllung eigener Aufgaben einer öffentlichen Stelle des Bundes aus zwingenden Gründen der Verteidigung oder der Erfüllung über- oder zwischenstaatlicher Verpflichtungen auf dem Gebiet der Krisenbewältigung oder Konfliktverhinderung oder für humanitäre Maßnahmen erforderlich ist.

(3) Die Angemessenheit des Schutzniveaus wird unter Berücksichtigung aller Umstände beurteilt, die bei einer Datenübermittlung oder einer Kategorie von Datenübermittlungen von Bedeutung sind; insbesondere können die Art der Daten, die Zweckbestimmung, die Dauer der geplanten Verarbeitung, das Herkunfts- und das Endbestimmungsland, die für den betreffenden Empfänger geltenden Rechtsnormen sowie die für ihn geltenden Standesregeln und Sicherheitsmaßnahmen herangezogen werden.

(4) In den Fällen des § 16 Abs. 1 Nr. 2 unterrichtet die übermittelnde Stelle den Betroffenen von der Übermittlung seiner Daten. Dies gilt nicht, wenn damit zu rechnen ist, dass er davon auf andere Weise Kenntnis erlangt, oder wenn die Unterrichtung die öffentliche Sicherheit gefährden oder sonst dem Wohl des Bundes oder eines Landes Nachteile bereiten würde.

(5) Die Verantwortung für die Zulässigkeit der Übermittlung trägt die übermittelnde Stelle.

(6) Die Stelle, an die die Daten übermittelt werden, ist auf den Zweck hinzuweisen, zu dessen Erfüllung die Daten übermittelt werden.

§ 4c Ausnahmen

(1) Im Rahmen von Tätigkeiten, die ganz oder teilweise in den Anwendungsbereich des Rechts der Europäischen Gemeinschaften fallen, ist eine Übermittlung personenbezogener Daten an andere als die in § 4b Abs. 1 genannten Stellen, auch wenn bei ihnen ein angemessenes Datenschutzniveau nicht gewährleistet ist, zulässig, sofern

1. der Betroffene seine Einwilligung gegeben hat,
2. die Übermittlung für die Erfüllung eines Vertrags zwischen dem Betroffenen und der verantwortlichen Stelle oder zur Durchführung von vorvertraglichen Maßnahmen, die auf Veranlassung des Betroffenen getroffen worden sind, erforderlich ist,
3. die Übermittlung zum Abschluss oder zur Erfüllung eines Vertrags erforderlich ist, der im Interesse des Betroffenen von der verantwortlichen Stelle mit einem Dritten geschlossen wurde oder geschlossen werden soll,
4. die Übermittlung für die Wahrung eines wichtigen öffentlichen Interesses oder zur Geltendmachung, Ausübung oder Verteidigung von Rechtsansprüchen vor Gericht erforderlich ist,
5. die Übermittlung für die Wahrung lebenswichtiger Interessen des Betroffenen erforderlich ist oder
6. die Übermittlung aus einem Register erfolgt, das zur Information der Öffentlichkeit bestimmt ist und entweder der gesamten Öffentlichkeit oder allen Personen, die ein berechtigtes Interesse nachweisen können, zur Einsichtnahme offen steht, soweit die gesetzlichen Voraussetzungen im Einzelfall gegeben sind.

Die Stelle, an die die Daten übermittelt werden, ist darauf hinzuweisen, dass die übermittelten Daten nur zu dem Zweck verarbeitet oder genutzt werden dürfen, zu dessen Erfüllung sie übermittelt werden.

(2) Unbeschadet des Absatzes 1 Satz 1 kann die zuständige Aufsichtsbehörde einzelne Übermittlungen oder bestimmte Arten von Übermittlungen personenbezogener Daten an andere als die in § 4b Abs. 1 genannten Stellen genehmigen, wenn die verantwortliche Stelle ausreichende Garantien hinsichtlich des Schutzes des Persönlichkeitsrechts und der Ausübung der damit verbundenen Rechte vorweist; die Garantien können sich insbesondere aus Vertragsklauseln oder verbindlichen Unternehmensregelungen ergeben. Bei den Post- und Telekommunikationsunternehmen ist der Bundesbeauftragte für den Datenschutz und die Informationsfreiheit zuständig. Sofern die Übermittlung durch öffentliche Stellen erfolgen soll, nehmen diese die Prüfung nach Satz 1 vor.

(3) Die Länder teilen dem Bund die nach Absatz 2 Satz 1 ergangenen Entscheidungen mit.

§ 4d Meldepflicht

(1) Verfahren automatisierter Verarbeitungen sind vor ihrer Inbetriebnahme von nicht-öffentlichen verantwortlichen Stellen der zuständigen Aufsichtsbehörde und von öffentlichen verantwortlichen Stellen des Bundes sowie von den Post- und Telekommunikationsunternehmen dem Bundesbeauftragten für den Datenschutz und die Informationsfreiheit nach Maßgabe von § 4e zu melden.

(2) Die Meldepflicht entfällt, wenn die verantwortliche Stelle einen Beauftragten für den Datenschutz bestellt hat.

(3) Die Meldepflicht entfällt ferner, wenn die verantwortliche Stelle personenbezogene Daten für eigene Zwecke erhebt, verarbeitet oder nutzt, hierbei in der Regel höchstens neun Personen ständig mit der Erhebung, Verarbeitung oder Nutzung personenbezogener Daten beschäftigt und entweder eine Einwilligung des Betroffenen vorliegt oder die Erhebung, Verarbeitung oder Nutzung für die Begründung, Durchführung oder Beendigung eines rechtsgeschäftlichen oder rechtsgeschäftsähnlichen Schuldverhältnisses mit dem Betroffenen erforderlich ist.

(4) Die Absätze 2 und 3 gelten nicht, wenn es sich um automatisierte Verarbeitungen handelt, in denen geschäftsmäßig personenbezogene Daten von der jeweiligen Stelle

1. zum Zweck der Übermittlung,
2. zum Zweck der anonymisierten Übermittlung oder
3. für Zwecke der Markt- oder Meinungsforschung

gespeichert werden.

(5) Soweit automatisierte Verarbeitungen besondere Risiken für die Rechte und Freiheiten der Betroffenen aufweisen, unterliegen sie der Prüfung vor Beginn der Verarbeitung (Vorabkontrolle). Eine Vorabkontrolle ist insbesondere durchzuführen, wenn

1. besondere Arten personenbezogener Daten (§ 3 Abs. 9) verarbeitet werden oder
2. die Verarbeitung personenbezogener Daten dazu bestimmt ist, die Persönlichkeit des Betroffenen zu bewerten einschließlich seiner Fähigkeiten, seiner Leistung oder seines Verhaltens,

es sei denn, dass eine gesetzliche Verpflichtung oder eine Einwilligung des Betroffenen vorliegt oder die Erhebung, Verarbeitung oder Nutzung für die Begründung, Durchführung oder Beendigung eines rechtsgeschäftlichen oder rechtsgeschäftsähnlichen Schuldverhältnisses mit dem Betroffenen erforderlich ist.

(6) Zuständig für die Vorabkontrolle ist der Beauftragte für den Datenschutz. Dieser nimmt die Vorabkontrolle nach Empfang der Übersicht nach § 4g Abs. 2 Satz 1 vor. Er hat sich in Zweifelsfällen an die Aufsichtsbehörde oder bei den Post- und Telekommunikationsunternehmen an den Bundesbeauftragten für den Datenschutz und die Informationsfreiheit zu wenden.

§ 4e Inhalt der Meldepflicht

Sofern Verfahren automatisierter Verarbeitungen meldepflichtig sind, sind folgende Angaben zu machen:

1. Name oder Firma der verantwortlichen Stelle,
2. Inhaber, Vorstände, Geschäftsführer oder sonstige gesetzliche oder nach der Verfassung des Unternehmens berufene Leiter und die mit der Leitung der Datenverarbeitung beauftragten Personen,
3. Anschrift der verantwortlichen Stelle,
4. Zweckbestimmungen der Datenerhebung, -verarbeitung oder -nutzung,
5. eine Beschreibung der betroffenen Personengruppen und der diesbezüglichen Daten oder Datenkategorien,
6. Empfänger oder Kategorien von Empfängern, denen die Daten mitgeteilt werden können,
7. Regelfristen für die Löschung der Daten,
8. eine geplante Datenübermittlung in Drittstaaten,
9. eine allgemeine Beschreibung, die es ermöglicht, vorläufig zu beurteilen, ob die Maßnahmen nach § 9 zur Gewährleistung der Sicherheit der Verarbeitung angemessen sind.

§ 4d Abs. 1 und 4 gilt für die Änderung der nach Satz 1 mitgeteilten Angaben sowie für den Zeitpunkt der Aufnahme und der Beendigung der meldepflichtigen Tätigkeit entsprechend.

§ 4f Beauftragter für den Datenschutz

(1) Öffentliche und nicht-öffentliche Stellen, die personenbezogene Daten automatisiert verarbeiten, haben einen Beauftragten für den Datenschutz schriftlich zu bestellen. Nicht-öffentliche Stellen sind hierzu spätestens innerhalb eines Monats nach Aufnahme ihrer Tätigkeit verpflichtet. Das Gleiche gilt, wenn personenbezogene Daten auf andere Weise erhoben, verarbeitet oder genutzt werden und damit in der Regel mindestens 20 Personen beschäftigt sind. Die Sätze 1 und 2 gelten nicht für die nichtöffentlichen Stellen, die in der Regel höchstens neun Personen ständig mit der automatisierten Verarbeitung personenbezogener Daten beschäftigen. Soweit aufgrund der Struktur einer öffentlichen Stelle erforderlich, genügt die Bestellung eines Beauftragten für den Datenschutz für mehrere Bereiche. Soweit nicht-öffentliche Stellen automatisierte Verarbeitungen vornehmen, die einer Vorabkontrolle unterliegen, oder personenbezogene Daten geschäftsmäßig zum Zweck der Übermittlung, der anonymisierten Übermittlung oder für Zwecke der Markt- oder Meinungsforschung automatisiert verarbeiten, haben sie unabhängig von der Anzahl der mit der automatisierten Verarbeitung beschäftigten Personen einen Beauftragten für den Datenschutz zu bestellen.

(2) Zum Beauftragten für den Datenschutz darf nur bestellt werden, wer die zur Erfüllung seiner Aufgaben erforderliche Fachkunde und Zuverlässigkeit besitzt. Das Maß der erforderlichen Fachkunde bestimmt sich insbesondere nach dem Umfang der Datenverarbeitung der verantwortlichen Stelle und dem Schutzbedarf der personenbezogenen Daten, die die verantwortliche Stelle erhebt oder verwendet. Zum Beauftragten für den Datenschutz kann auch eine Person außerhalb der verantwortlichen Stelle bestellt werden; die Kontrolle erstreckt sich auch auf personenbezogene Daten, die einem Berufs- oder besonderen Amtsgeheimnis, insbesondere dem Steuergeheimnis nach § 30 der Abgabenordnung, unterliegen. Öffentliche Stellen können mit Zustimmung ihrer Aufsichtsbehörde einen Bediensteten aus einer anderen öffentlichen Stelle zum Beauftragten für den Datenschutz bestellen.

(3) Der Beauftragte für den Datenschutz ist dem Leiter der öffentlichen oder nicht-öffentlichen Stelle unmittelbar zu unterstellen. Er ist in Ausübung seiner Fachkunde auf dem Gebiet des Datenschutzes weisungsfrei. Er darf wegen der Erfüllung seiner Aufgaben nicht benachteiligt werden. Die Bestellung zum Beauftragten für den Datenschutz kann in entsprechender Anwendung von § 626 des Bürgerlichen Gesetzbuches, bei nicht-öffentlichen Stellen auch auf Verlangen der Aufsichtsbehörde, widerrufen werden. Ist nach Absatz 1 ein Beauftragter für den Datenschutz zu bestellen, so ist die Kündigung des Arbeitsverhältnisses unzulässig, es sei denn, dass Tatsachen vorliegen, welche die verantwortliche Stelle zur Kündigung aus wichtigem Grund ohne Einhaltung einer Kündigungsfrist berechtigen. Nach der Abberufung als Beauftragter für den Datenschutz ist die Kündigung innerhalb eines Jahres nach der Beendigung der Bestellung unzulässig, es sei denn, dass die verantwortliche Stelle zur Kündigung aus wichtigem Grund ohne Einhaltung einer Kündigungsfrist berechtigt ist. Zur Erhaltung der zur Erfüllung seiner Aufgaben erforderlichen Fachkunde hat die verantwortliche Stelle dem Beauftragten für den Datenschutz die Teilnahme an Fort- und Weiterbildungsveranstaltungen zu ermöglichen und deren Kosten zu übernehmen.

(4) Der Beauftragte für den Datenschutz ist zur Verschwiegenheit über die Identität des Betroffenen sowie über Umstände, die Rückschlüsse auf den Betroffenen zulassen, verpflichtet, soweit er nicht davon durch den Betroffenen befreit wird.

(4a) Soweit der Beauftragte für den Datenschutz bei seiner Tätigkeit Kenntnis von Daten erhält, für die dem Leiter oder einer bei der öffentlichen oder nichtöffentlichen Stelle beschäftigten Person aus beruflichen Gründen ein Zeugnisverweigerungsrecht zusteht, steht dieses Recht auch dem Beauftragten für den Datenschutz und dessen Hilfspersonal zu. Über die Ausübung dieses Rechts entscheidet die Person, der das Zeugnisverweigerungsrecht aus beruflichen Gründen zusteht, es sei denn, dass diese Entscheidung in absehbarer Zeit nicht herbeigeführt werden kann. Soweit das Zeugnisverweigerungsrecht des Beauftragten für den Datenschutz reicht, unterliegen seine Akten und andere Schriftstücke einem Beschlagnahmeverbot.

(5) Die öffentlichen und nicht-öffentlichen Stellen haben den Beauftragten für den Datenschutz bei der Erfüllung seiner Aufgaben zu unterstützen und ihm insbesondere, soweit dies zur Erfüllung seiner Aufgaben erforderlich ist, Hilfspersonal sowie Räume, Einrichtungen, Geräte und Mittel zur Verfügung zu stellen. Betroffene können sich jederzeit an den Beauftragten für den Datenschutz wenden.

§ 4g Aufgaben des Beauftragten für den Datenschutz

(1) Der Beauftragte für den Datenschutz wirkt auf die Einhaltung dieses Gesetzes und anderer Vorschriften über den Datenschutz hin. Zu diesem Zweck kann sich der Beauftragte für den Datenschutz in Zweifelsfällen an die für die Datenschutzkontrolle bei der verantwortlichen Stelle zuständige Behörde wenden. Er kann die Beratung nach § 38 Abs. 1 Satz 2 in Anspruch nehmen. Er hat insbesondere

1. die ordnungsgemäße Anwendung der Datenverarbeitungsprogramme, mit deren Hilfe personenbezogene Daten verarbeitet werden sollen, zu überwachen; zu diesem Zweck ist er über Vorhaben der automatisierten Verarbeitung personenbezogener Daten rechtzeitig zu unterrichten,
2. die bei der Verarbeitung personenbezogener Daten tätigen Personen durch geeignete Maßnahmen mit den Vorschriften dieses Gesetzes sowie anderen Vorschriften über den Datenschutz und mit den jeweiligen besonderen Erfordernissen des Datenschutzes vertraut zu machen.

(2) Dem Beauftragten für den Datenschutz ist von der verantwortlichen Stelle eine Übersicht über die in § 4e Satz 1 genannten Angaben sowie über zugriffsberechtigte Personen zur Verfügung zu stellen. Der Beauftragte für den Datenschutz macht die Angaben nach § 4e Satz 1 Nr. 1 bis 8 auf Antrag jedermann in geeigneter Weise verfügbar.

(2a) Soweit bei einer nichtöffentlichen Stelle keine Verpflichtung zur Bestellung eines Beauftragten für den Datenschutz besteht, hat der Leiter der nichtöffentlichen Stelle die Erfüllung der Aufgaben nach den Absätzen 1 und 2 in anderer Weise sicherzustellen.

(3) Auf die in § 6 Abs. 2 Satz 4 genannten Behörden findet Absatz 2 Satz 2 keine Anwendung. Absatz 1 Satz 2 findet mit der Maßgabe Anwendung, dass der behördliche Beauftragte für den Datenschutz das Benehmen mit dem Behördenleiter herstellt; bei Unstimmigkeiten zwischen dem behördlichen Beauftragten für den Datenschutz und dem Behördenleiter entscheidet die oberste Bundesbehörde.

§ 5 Datengeheimnis

Den bei der Datenverarbeitung beschäftigten Personen ist untersagt, personenbezogene Daten unbefugt zu erheben, zu verarbeiten oder zu nutzen (Datengeheimnis). Diese Personen sind, soweit sie bei nicht-öffentlichen Stellen beschäftigt werden, bei der Aufnahme ihrer Tätigkeit auf das Datengeheimnis zu verpflichten. Das Datengeheimnis besteht auch nach Beendigung ihrer Tätigkeit fort.

§ 6 Rechte des Betroffenen

(1) Die Rechte des Betroffenen auf Auskunft (§§ 19, 34) und auf Berichtigung, Löschung oder Sperrung (§§ 20, 35) können nicht durch Rechtsgeschäft ausgeschlossen oder beschränkt werden.

(2) Sind die Daten des Betroffenen automatisiert in der Weise gespeichert, dass mehrere Stellen speicherungsberechtigt sind, und ist der Betroffene nicht in der Lage festzustellen, welche Stelle die Daten gespeichert hat, so kann er sich an jede dieser Stellen wenden. Diese ist verpflichtet, das Vorbringen des Betroffenen an die Stelle, die die Daten gespeichert hat, weiterzuleiten. Der Betroffene ist über die Weiterleitung und jene Stelle zu unterrichten. Die in § 19 Abs. 3 genannten Stellen, die Behörden der Staatsanwaltschaft und der Polizei sowie öffentliche Stellen der Finanzverwaltung, soweit sie personenbezogene Daten in Erfüllung ihrer gesetzlichen Aufgaben im Anwendungsbereich der Abgabenordnung zur Überwachung und Prüfung speichern, können statt des Betroffenen den Bundesbeauftragten für den Datenschutz und die Informationsfreiheit unterrichten. In diesem Fall richtet sich das weitere Verfahren nach § 19 Abs. 6.

(3) Personenbezogene Daten über die Ausübung eines Rechts des Betroffenen, das sich aus diesem Gesetz oder aus einer anderen Vorschrift über den Datenschutz ergibt, dürfen nur zur Erfüllung der sich aus der Ausübung des Rechts ergebenden Pflichten der verantwortlichen Stelle verwendet werden.

§ 6a Automatisierte Einzelentscheidung

(1) Entscheidungen, die für den Betroffenen eine rechtliche Folge nach sich ziehen oder ihn erheblich beeinträchtigen, dürfen nicht ausschließlich auf eine automatisierte Verarbeitung personenbezogener Daten gestützt werden, die der Bewertung einzelner Persönlichkeitsmerkmale dienen. Eine ausschließlich auf eine automatisierte Verarbeitung gestützte Entscheidung liegt insbesondere dann vor, wenn keine inhaltliche Bewertung und darauf gestützte Entscheidung durch eine natürliche Person stattgefunden hat.

(2) Dies gilt nicht, wenn

1. die Entscheidung im Rahmen des Abschlusses oder der Erfüllung eines Vertragsverhältnisses oder eines sonstigen Rechtsverhältnisses ergeht und dem Begehren des Betroffenen stattgegeben wurde oder
2. die Wahrung der berechtigten Interessen des Betroffenen durch geeignete Maßnahmen gewährleistet ist und die verantwortliche Stelle dem Betroffenen die Tatsache des Vorliegens einer Entscheidung im Sinne des Absatzes 1 mitteilt sowie auf Verlangen die wesentlichen Gründe dieser Entscheidung mitteilt und erläutert.

(3) Das Recht des Betroffenen auf Auskunft nach den §§ 19 und 34 erstreckt sich auch auf den logischen Aufbau der automatisierten Verarbeitung der ihn betreffenden Daten.

§ 6b Beobachtung öffentlich zugänglicher Räume mit optisch-elektronischen Einrichtungen

(1) Die Beobachtung öffentlich zugänglicher Räume mit optisch-elektronischen Einrichtungen (Videoüberwachung) ist nur zulässig, soweit sie

1. zur Aufgabenerfüllung öffentlicher Stellen,
2. zur Wahrnehmung des Hausrechts oder
3. zur Wahrnehmung berechtigter Interessen für konkret festgelegte Zwecke

erforderlich ist und keine Anhaltspunkte bestehen, dass schutzwürdige Interessen der Betroffenen überwiegen.

(2) Der Umstand der Beobachtung und die verantwortliche Stelle sind durch geeignete Maßnahmen erkennbar zu machen.

(3) Die Verarbeitung oder Nutzung von nach Absatz 1 erhobenen Daten ist zulässig, wenn sie zum Erreichen des verfolgten Zwecks erforderlich ist und keine Anhaltspunkte bestehen, dass schutzwürdige Interessen der Betroffenen überwiegen. Für einen anderen Zweck dürfen sie nur verarbeitet oder genutzt werden, soweit

dies zur Abwehr von Gefahren für die staatliche und öffentliche Sicherheit sowie zur Verfolgung von Straftaten erforderlich ist.

(4) Werden durch Videoüberwachung erhobene Daten einer bestimmten Person zugeordnet, ist diese über eine Verarbeitung oder Nutzung entsprechend den §§ 19a und 33 zu benachrichtigen.

(5) Die Daten sind unverzüglich zu löschen, wenn sie zur Erreichung des Zwecks nicht mehr erforderlich sind oder schutzwürdige Interessen der Betroffenen einer weiteren Speicherung entgegenstehen.

§ 6c Mobile personenbezogene Speicher- und Verarbeitungsmedien

(1) Die Stelle, die ein mobiles personenbezogenes Speicher- und Verarbeitungsmedium ausgibt oder ein Verfahren zur automatisierten Verarbeitung personenbezogener Daten, das ganz oder teilweise auf einem solchen Medium abläuft, auf das Medium aufbringt, ändert oder hierzu bereithält, muss den Betroffenen

1. über ihre Identität und Anschrift,
2. in allgemein verständlicher Form über die Funktionsweise des Mediums einschließlich der Art der zu verarbeitenden personenbezogenen Daten,
3. darüber, wie er seine Rechte nach den §§ 19, 20, 34 und 35 ausüben kann, und
4. über die bei Verlust oder Zerstörung des Mediums zu treffenden Maßnahmen

unterrichten, soweit der Betroffene nicht bereits Kenntnis erlangt hat.

(2) Die nach Absatz 1 verpflichtete Stelle hat dafür Sorge zu tragen, dass die zur Wahrnehmung des Auskunftsrechts erforderlichen Geräte oder Einrichtungen in angemessenem Umfang zum unentgeltlichen Gebrauch zur Verfügung stehen.

(3) Kommunikationsvorgänge, die auf dem Medium eine Datenverarbeitung auslösen, müssen für den Betroffenen eindeutig erkennbar sein.

§ 7 Schadensersatz

Fügt eine verantwortliche Stelle dem Betroffenen durch eine nach diesem Gesetz oder nach anderen Vorschriften über den Datenschutz unzulässige oder unrichtige Erhebung, Verarbeitung oder Nutzung seiner personenbezogenen Daten einen Schaden zu, ist sie oder ihr Träger dem Betroffenen zum Schadensersatz verpflichtet. Die Ersatzpflicht entfällt, soweit die verantwortliche Stelle die nach den Umständen des Falles gebotene Sorgfalt beachtet hat.

§ 8 Schadensersatz bei automatisierter Datenverarbeitung durch öffentliche Stellen

(1) Fügt eine verantwortliche öffentliche Stelle dem Betroffenen durch eine nach diesem Gesetz oder nach anderen Vorschriften über den Datenschutz unzulässige oder unrichtige automatisierte Erhebung, Verarbeitung oder Nutzung seiner personenbezogenen Daten einen Schaden zu, ist ihr Träger dem Betroffenen unabhängig von einem Verschulden zum Schadensersatz verpflichtet.

(2) Bei einer schweren Verletzung des Persönlichkeitsrechts ist dem Betroffenen der Schaden, der nicht Vermögensschaden ist, angemessen in Geld zu ersetzen.

(3) Die Ansprüche nach den Absätzen 1 und 2 sind insgesamt auf einen Betrag von 130.000 Euro begrenzt. Ist aufgrund desselben Ereignisses an mehrere Personen Schadensersatz zu leisten, der insgesamt den Höchstbetrag von 130.000 Euro übersteigt, so verringern sich die einzelnen Schadensersatzleistungen in dem Verhältnis, in dem ihr Gesamtbetrag zu dem Höchstbetrag steht.

(4) Sind bei einer automatisierten Verarbeitung mehrere Stellen speicherungsberechtigt und ist der Geschädigte nicht in der Lage, die speichernde Stelle festzustellen, so haftet jede dieser Stellen.

(5) Hat bei der Entstehung des Schadens ein Verschulden des Betroffenen mitgewirkt, gilt § 254 des Bürgerlichen Gesetzbuchs.

(6) Auf die Verjährung finden die für unerlaubte Handlungen geltenden Verjährungsvorschriften des Bürgerlichen Gesetzbuchs entsprechende Anwendung.

§ 9 Technische und organisatorische Maßnahmen

Öffentliche und nicht-öffentliche Stellen, die selbst oder im Auftrag personenbezogene Daten erheben, verarbeiten oder nutzen, haben die technischen und organisatorischen Maßnahmen zu treffen, die erforderlich sind, um die Ausführung der Vorschriften dieses Gesetzes, insbesondere die in der Anlage zu diesem Gesetz genannten Anforderungen, zu gewährleisten. Erforderlich sind Maßnahmen nur, wenn ihr Aufwand in einem angemessenen Verhältnis zu dem angestrebten Schutzzweck steht.

§ 9a Datenschutzaudit

Zur Verbesserung des Datenschutzes und der Datensicherheit können Anbieter von Datenverarbeitungssystemen und -programmen und datenverarbeitende Stellen ihr Datenschutzkonzept sowie ihre technischen Einrichtungen durch unabhängige und zugelassene Gutachter prüfen und bewerten lassen sowie das Ergebnis der Prüfung veröffentlichen. Die näheren Anforderungen an die Prüfung und Bewertung, das Verfahren sowie die Auswahl und Zulassung der Gutachter werden durch besonderes Gesetz geregelt.

§ 10 Einrichtung automatisierter Abrufverfahren

(1) Die Einrichtung eines automatisierten Verfahrens, das die Übermittlung personenbezogener Daten durch Abruf ermöglicht, ist zulässig, soweit dieses Verfahren unter Berücksichtigung der schutzwürdigen Interessen der Betroffenen und der Aufgaben oder Geschäftszwecke der beteiligten Stellen angemessen ist. Die Vorschriften über die Zulässigkeit des einzelnen Abrufs bleiben unberührt.

(2) Die beteiligten Stellen haben zu gewährleisten, dass die Zulässigkeit des Abrufverfahrens kontrolliert werden kann. Hierzu haben sie schriftlich festzulegen:

1. Anlass und Zweck des Abrufverfahrens,
2. Dritte, an die übermittelt wird,
3. Art der zu übermittelnden Daten,
4. nach § 9 erforderliche technische und organisatorische Maßnahmen.

Im öffentlichen Bereich können die erforderlichen Festlegungen auch durch die Fachaufsichtsbehörden getroffen werden.

(3) Über die Einrichtung von Abrufverfahren ist in Fällen, in denen die in § 12 Abs. 1 genannten Stellen beteiligt sind, der Bundesbeauftragte für den Datenschutz und die Informationsfreiheit unter Mitteilung der Festlegungen nach Absatz 2 zu unterrichten. Die Einrichtung von Abrufverfahren, bei denen die in § 6 Abs. 2 und in § 19 Abs. 3 genannten Stellen beteiligt sind, ist nur zulässig, wenn das für die speichernde und die abrufende Stelle jeweils zuständige Bundes- oder Landesministerium zugestimmt hat.

(4) Die Verantwortung für die Zulässigkeit des einzelnen Abrufs trägt der Dritte, an den übermittelt wird. Die speichernde Stelle prüft die Zulässigkeit der Abrufe nur, wenn dazu Anlass besteht. Die speichernde Stelle hat zu gewährleisten, dass die Übermittlung personenbezogener Daten zumindest durch geeignete Stichprobenverfahren festgestellt und überprüft werden kann. Wird ein Gesamtbestand personenbezogener Daten abgerufen oder übermittelt (Stapelverarbeitung), so bezieht sich die Gewährleistung der Feststellung und Überprüfung nur auf die Zulässigkeit des Abrufes oder der Übermittlung des Gesamtbestandes.

(5) Die Absätze 1 bis 4 gelten nicht für den Abruf allgemein zugänglicher Daten. Allgemein zugänglich sind Daten, die jedermann, sei es ohne oder nach vorheriger Anmeldung, Zulassung oder Entrichtung eines Entgelts, nutzen kann.

§ 11 Erhebung, Verarbeitung oder Nutzung personenbezogener Daten im Auftrag

(1) Werden personenbezogene Daten im Auftrag durch andere Stellen erhoben, verarbeitet oder genutzt, ist der Auftraggeber für die Einhaltung der Vorschriften dieses Gesetzes und anderer Vorschriften über den Datenschutz verantwortlich. Die in den §§ 6, 7 und 8 genannten Rechte sind ihm gegenüber geltend zu machen.

(2) Der Auftragnehmer ist unter besonderer Berücksichtigung der Eignung der von ihm getroffenen technischen und organisatorischen Maßnahmen sorgfältig auszuwählen. Der Auftrag ist schriftlich zu erteilen, wobei insbesondere im Einzelnen festzulegen sind:

1. der Gegenstand und die Dauer des Auftrags,
2. der Umfang, die Art und der Zweck der vorgesehenen Erhebung, Verarbeitung oder Nutzung von Daten, die Art der Daten und der Kreis der Betroffenen,

3. die nach § 9 zu treffenden technischen und organisatorischen Maßnahmen,
4. die Berichtigung, Löschung und Sperrung von Daten,
5. die nach Absatz 4 bestehenden Pflichten des Auftragnehmers, insbesondere die von ihm vorzunehmenden Kontrollen,
6. die etwaige Berechtigung zur Begründung von Unterauftragsverhältnissen,
7. die Kontrollrechte des Auftraggebers und die entsprechenden Duldungs- und Mitwirkungspflichten des Auftragnehmers,
8. mitzuteilende Verstöße des Auftragnehmers oder der bei ihm beschäftigten Personen gegen Vorschriften zum Schutz personenbezogener Daten oder gegen die im Auftrag getroffenen Festlegungen,
9. der Umfang der Weisungsbefugnisse, die sich der Auftraggeber gegenüber dem Auftragnehmer vorbehält,
10. die Rückgabe überlassener Datenträger und die Löschung beim Auftragnehmer gespeicherter Daten nach Beendigung des Auftrags.

Er kann bei öffentlichen Stellen auch durch die Fachaufsichtsbehörde erteilt werden. Der Auftraggeber hat sich vor Beginn der Datenverarbeitung und sodann regelmäßig von der Einhaltung der beim Auftragnehmer getroffenen technischen und organisatorischen Maßnahmen zu überzeugen. Das Ergebnis ist zu dokumentieren.

(3) Der Auftragnehmer darf die Daten nur im Rahmen der Weisungen des Auftraggebers erheben, verarbeiten oder nutzen. Ist er der Ansicht, dass eine Weisung des Auftraggebers gegen dieses Gesetz oder andere Vorschriften über den Datenschutz verstößt, hat er den Auftraggeber unverzüglich darauf hinzuweisen.

(4) Für den Auftragnehmer gelten neben den §§ 5, 9, 43 Abs. 1 Nr. 2, 10 und 11, Abs. 2 Nr. 1 bis 3 und Abs. 3 sowie § 44 nur die Vorschriften über die Datenschutzkontrolle oder die Aufsicht, und zwar für

1.
   a) öffentliche Stellen,
   b) nicht-öffentliche Stellen, bei denen der öffentlichen Hand die Mehrheit der Anteile gehört oder die Mehrheit der Stimmen zusteht und der Auftraggeber eine öffentliche Stelle ist,

   die §§ 18, 24 bis 26 oder die entsprechenden Vorschriften der Datenschutzgesetze der Länder,
2. die übrigen nicht-öffentlichen Stellen, soweit sie personenbezogene Daten im Auftrag als Dienstleistungsunternehmen geschäftsmäßig erheben, verarbeiten oder nutzen, die §§ 4f, 4g und 38.

(5) Die Absätze 1 bis 4 gelten entsprechend, wenn die Prüfung oder Wartung automatisierter Verfahren oder von Datenverarbeitungsanlagen durch andere Stellen im Auftrag vorgenommen wird und dabei ein Zugriff auf personenbezogene Daten nicht ausgeschlossen werden kann.

Zweiter Abschnitt
Datenverarbeitung der öffentlichen Stellen

Erster Unterabschnitt
Rechtsgrundlagen der Datenverarbeitung

§ 12 Anwendungsbereich

(1) Die Vorschriften dieses Abschnittes gelten für öffentliche Stellen des Bundes, soweit sie nicht als öffentlich-rechtliche Unternehmen am Wettbewerb teilnehmen.

(2) Soweit der Datenschutz nicht durch Landesgesetz geregelt ist, gelten die §§ 12 bis 16, 19 bis 20 auch für die öffentlichen Stellen der Länder, soweit sie

1. Bundesrecht ausführen und nicht als öffentlich-rechtliche Unternehmen am Wettbewerb teilnehmen oder
2. als Organe der Rechtspflege tätig werden und es sich nicht um Verwaltungsangelegenheiten handelt.

(3) Für Landesbeauftragte für den Datenschutz gilt § 23 Abs. 4 entsprechend.

(4) Werden personenbezogene Daten für frühere, bestehende oder zukünftige Beschäftigungsverhältnisse erhoben, verarbeitet oder genutzt, gelten § 28 Absatz 2 Nummer 2 und die §§ 32 bis 35 anstelle der §§ 13 bis 16 und 19 bis 20.

§ 13 Datenerhebung

(1) Das Erheben personenbezogener Daten ist zulässig, wenn ihre Kenntnis zur Erfüllung der Aufgaben der verantwortlichen Stelle erforderlich ist.

(1a) Werden personenbezogene Daten statt beim Betroffenen bei einer nicht-öffentlichen Stelle erhoben, so ist die Stelle auf die Rechtsvorschrift, die zur Auskunft verpflichtet, sonst auf die Freiwilligkeit ihrer Angaben hinzuweisen.

(2) Das Erheben besonderer Arten personenbezogener Daten (§ 3 Abs. 9) ist nur zulässig, soweit

1. eine Rechtsvorschrift dies vorsieht oder aus Gründen eines wichtigen öffentlichen Interesses zwingend erfordert,
2. der Betroffene nach Maßgabe des § 4a Abs. 3 eingewilligt hat,
3. dies zum Schutz lebenswichtiger Interessen des Betroffenen oder eines Dritten erforderlich ist, sofern der Betroffene aus physischen oder rechtlichen Gründen außerstande ist, seine Einwilligung zu geben,
4. es sich um Daten handelt, die der Betroffene offenkundig öffentlich gemacht hat,
5. dies zur Abwehr einer erheblichen Gefahr für die öffentliche Sicherheit erforderlich ist,
6. dies zur Abwehr erheblicher Nachteile für das Gemeinwohl oder zur Wahrung erheblicher Belange des Gemeinwohls zwingend erforderlich ist,
7. dies zum Zweck der Gesundheitsvorsorge, der medizinischen Diagnostik, der Gesundheitsversorgung oder Behandlung oder für die Verwaltung von Gesundheitsdiensten erforderlich ist und die Verarbeitung dieser Daten durch ärztliches Personal oder durch sonstige Personen erfolgt, die einer entsprechenden Geheimhaltungspflicht unterliegen,
8. dies zur Durchführung wissenschaftlicher Forschung erforderlich ist, das wissenschaftliche Interesse an der Durchführung des Forschungsvorhabens das Interesse des Betroffenen an dem Ausschluss der Erhebung erheblich überwiegt und der Zweck der Forschung auf andere Weise nicht oder nur mit unverhältnismäßigem Aufwand erreicht werden kann oder
9. dies aus zwingenden Gründen der Verteidigung oder der Erfüllung über- oder zwischenstaatlicher Verpflichtungen einer öffentlichen Stelle des Bundes auf dem Gebiet der Krisenbewältigung oder Konfliktverhinderung oder für humanitäre Maßnahmen erforderlich ist.

§ 14 Datenspeicherung, -veränderung und -nutzung

(1) Das Speichern, Verändern oder Nutzen personenbezogener Daten ist zulässig, wenn es zur Erfüllung der in der Zuständigkeit der verantwortlichen Stelle liegenden Aufgaben erforderlich ist und es für die Zwecke erfolgt, für die die Daten erhoben worden sind. Ist keine Erhebung vorausgegangen, dürfen die Daten nur für die Zwecke geändert oder genutzt werden, für die sie gespeichert worden sind.

(2) Das Speichern, Verändern oder Nutzen für andere Zwecke ist nur zulässig, wenn

1. eine Rechtsvorschrift dies vorsieht oder zwingend voraussetzt,
2. der Betroffene eingewilligt hat,
3. offensichtlich ist, dass es im Interesse des Betroffenen liegt, und kein Grund zu der Annahme besteht, dass er in Kenntnis des anderen Zwecks seine Einwilligung verweigern würde,
4. Angaben des Betroffenen überprüft werden müssen, weil tatsächliche Anhaltspunkte für deren Unrichtigkeit bestehen,
5. die Daten allgemein zugänglich sind oder die verantwortliche Stelle sie veröffentlichen dürfte, es sei denn, dass das schutzwürdige Interesse des Betroffenen an dem Ausschluss der Zweckänderung offensichtlich überwiegt,
6. es zur Abwehr erheblicher Nachteile für das Gemeinwohl oder einer Gefahr für die öffentliche Sicherheit oder zur Wahrung erheblicher Belange des Gemeinwohls erforderlich ist,

7. es zur Verfolgung von Straftaten oder Ordnungswidrigkeiten, zur Vollstreckung oder zum Vollzug von Strafen oder Maßnahmen im Sinne des § 11 Abs. 1 Nr. 8 des Strafgesetzbuchs oder von Erziehungsmaßregeln oder Zuchtmitteln im Sinne des Jugendgerichtsgesetzes oder zur Vollstreckung von Bußgeldentscheidungen erforderlich ist,
8. es zur Abwehr einer schwerwiegenden Beeinträchtigung der Rechte einer anderen Person erforderlich ist oder
9. es zur Durchführung wissenschaftlicher Forschung erforderlich ist, das wissenschaftliche Interesse an der Durchführung des Forschungsvorhabens das Interesse des Betroffenen an dem Ausschluss der Zweckänderung erheblich überwiegt und der Zweck der Forschung auf andere Weise nicht oder nur mit unverhältnismäßigem Aufwand erreicht werden kann.

(3) Eine Verarbeitung oder Nutzung für andere Zwecke liegt nicht vor, wenn sie der Wahrnehmung von Aufsichts- und Kontrollbefugnissen, der Rechnungsprüfung oder der Durchführung von Organisationsuntersuchungen für die verantwortliche Stelle dient. Das gilt auch für die Verarbeitung oder Nutzung zu Ausbildungs- und Prüfungszwecken durch die verantwortliche Stelle, soweit nicht überwiegende schutzwürdige Interessen des Betroffenen entgegenstehen.

(4) Personenbezogene Daten, die ausschließlich zu Zwecken der Datenschutzkontrolle, der Datensicherung oder zur Sicherstellung eines ordnungsgemäßen Betriebes einer Datenverarbeitungsanlage gespeichert werden, dürfen nur für diese Zwecke verwendet werden.

(5) Das Speichern, Verändern oder Nutzen von besonderen Arten personenbezogener Daten (§ 3 Abs. 9) für andere Zwecke ist nur zulässig, wenn

1. die Voraussetzungen vorliegen, die eine Erhebung nach § 13 Abs. 2 Nr. 1 bis 6 oder 9 zulassen würden oder
2. dies zur Durchführung wissenschaftlicher Forschung erforderlich ist, das öffentliche Interesse an der Durchführung des Forschungsvorhabens das Interesse des Betroffenen an dem Ausschluss der Zweckänderung erheblich überwiegt und der Zweck der Forschung auf andere Weise nicht oder nur mit unverhältnismäßigem Aufwand erreicht werden kann.

Bei der Abwägung nach Satz 1 Nr. 2 ist im Rahmen des öffentlichen Interesses das wissenschaftliche Interesse an dem Forschungsvorhaben besonders zu berücksichtigen.

(6) Die Speicherung, Veränderung oder Nutzung von besonderen Arten personenbezogener Daten (§ 3 Abs. 9) zu den in § 13 Abs. 2 Nr. 7 genannten Zwecken richtet sich nach den für die in § 13 Abs. 2 Nr. 7 genannten Personen geltenden Geheimhaltungspflichten.

§ 15 Datenübermittlung an öffentliche Stellen

(1) Die Übermittlung personenbezogener Daten an öffentliche Stellen ist zulässig, wenn

1. sie zur Erfüllung der in der Zuständigkeit der übermittelnden Stelle oder des Dritten, an den die Daten übermittelt werden, liegenden Aufgaben erforderlich ist und
2. die Voraussetzungen vorliegen, die eine Nutzung nach § 14 zulassen würden.

(2) Die Verantwortung für die Zulässigkeit der Übermittlung trägt die übermittelnde Stelle. Erfolgt die Übermittlung auf Ersuchen des Dritten, an den die Daten übermittelt werden, trägt dieser die Verantwortung. In diesem Fall prüft die übermittelnde Stelle nur, ob das Übermittlungsersuchen im Rahmen der Aufgaben des Dritten, an den die Daten übermittelt werden, liegt, es sei denn, dass besonderer Anlass zur Prüfung der Zulässigkeit der Übermittlung besteht. § 10 Abs. 4 bleibt unberührt.

(3) Der Dritte, an den die Daten übermittelt werden, darf diese für den Zweck verarbeiten oder nutzen, zu dessen Erfüllung sie ihm übermittelt werden. Eine Verarbeitung oder Nutzung für andere Zwecke ist nur unter den Voraussetzungen des § 14 Abs. 2 zulässig.

(4) Für die Übermittlung personenbezogener Daten an Stellen der öffentlich-rechtlichen Religionsgesellschaften gelten die Absätze 1 bis 3 entsprechend, sofern sichergestellt ist, dass bei diesen ausreichende Datenschutzmaßnahmen getroffen werden.

(5) Sind mit personenbezogenen Daten, die nach Absatz 1 übermittelt werden dürfen, weitere personenbezogene Daten des Betroffenen oder eines Dritten so verbunden, dass eine Trennung nicht oder nur mit unvertretbarem Aufwand möglich ist, so ist die Übermittlung auch dieser Daten zulässig, soweit nicht berechtigte Interessen des

Betroffenen oder eines Dritten an deren Geheimhaltung offensichtlich überwiegen; eine Nutzung dieser Daten ist unzulässig.

(6) Absatz 5 gilt entsprechend, wenn personenbezogene Daten innerhalb einer öffentlichen Stelle weitergegeben werden.

§ 16 Datenübermittlung an nicht-öffentliche Stellen

(1) Die Übermittlung personenbezogener Daten an nicht-öffentliche Stellen ist zulässig, wenn

1. sie zur Erfüllung der in der Zuständigkeit der übermittelnden Stelle liegenden Aufgaben erforderlich ist und die Voraussetzungen vorliegen, die eine Nutzung nach § 14 zulassen würden, oder
2. der Dritte, an den die Daten übermittelt werden, ein berechtigtes Interesse an der Kenntnis der zu übermittelnden Daten glaubhaft darlegt und der Betroffene kein schutzwürdiges Interesse an dem Ausschluss der Übermittlung hat. Das Übermitteln von besonderen Arten personenbezogener Daten (§ 3 Abs. 9) ist abweichend von Satz 1 Nr. 2 nur zulässig, wenn die Voraussetzungen vorliegen, die eine Nutzung nach § 14 Abs. 5 und 6 zulassen würden oder soweit dies zur Geltendmachung, Ausübung oder Verteidigung rechtlicher Ansprüche erforderlich ist.

(2) Die Verantwortung für die Zulässigkeit der Übermittlung trägt die übermittelnde Stelle.

(3) In den Fällen der Übermittlung nach Absatz 1 Nr. 2 unterrichtet die übermittelnde Stelle den Betroffenen von der Übermittlung seiner Daten. Dies gilt nicht, wenn damit zu rechnen ist, dass er davon auf andere Weise Kenntnis erlangt, oder wenn die Unterrichtung die öffentliche Sicherheit gefährden oder sonst dem Wohle des Bundes oder eines Landes Nachteile bereiten würde.

(4) Der Dritte, an den die Daten übermittelt werden, darf diese nur für den Zweck verarbeiten oder nutzen, zu dessen Erfüllung sie ihm übermittelt werden. Die übermittelnde Stelle hat ihn darauf hinzuweisen. Eine Verarbeitung oder Nutzung für andere Zwecke ist zulässig, wenn eine Übermittlung nach Absatz 1 zulässig wäre und die übermittelnde Stelle zugestimmt hat.

§ 17

(weggefallen)

§ 18 Durchführung des Datenschutzes in der Bundesverwaltung

(1) Die obersten Bundesbehörden, der Präsident des Bundeseisenbahnvermögens sowie die bundesunmittelbaren Körperschaften, Anstalten und Stiftungen des öffentlichen Rechts, über die von der Bundesregierung oder einer obersten Bundesbehörde lediglich die Rechtsaufsicht ausgeübt wird, haben für ihren Geschäftsbereich die Ausführung dieses Gesetzes sowie anderer Rechtsvorschriften über den Datenschutz sicherzustellen. Das Gleiche gilt für die Vorstände der aus dem Sondervermögen Deutsche Bundespost durch Gesetz hervorgegangenen Unternehmen, solange diesen ein ausschließliches Recht nach dem Postgesetz zusteht.

(2) Die öffentlichen Stellen führen ein Verzeichnis der eingesetzten Datenverarbeitungsanlagen. Für ihre automatisierten Verarbeitungen haben sie die Angaben nach § 4e sowie die Rechtsgrundlage der Verarbeitung schriftlich festzulegen. Bei allgemeinen Verwaltungszwecken dienenden automatisierten Verarbeitungen, bei welchen das Auskunftsrecht des Betroffenen nicht nach § 19 Abs. 3 oder 4 eingeschränkt wird, kann hiervon abgesehen werden. Für automatisierte Verarbeitungen, die in gleicher oder ähnlicher Weise mehrfach geführt werden, können die Festlegungen zusammengefasst werden.

Zweiter Unterabschnitt
Rechte des Betroffenen

§ 19 Auskunft an den Betroffenen

(1) Dem Betroffenen ist auf Antrag Auskunft zu erteilen über

1. die zu seiner Person gespeicherten Daten, auch soweit sie sich auf die Herkunft dieser Daten beziehen,
2. die Empfänger oder Kategorien von Empfängern, an die die Daten weitergegeben werden, und

3. den Zweck der Speicherung.

In dem Antrag soll die Art der personenbezogenen Daten, über die Auskunft erteilt werden soll, näher bezeichnet werden. Sind die personenbezogenen Daten weder automatisiert noch in nicht automatisierten Dateien gespeichert, wird die Auskunft nur erteilt, soweit der Betroffene Angaben macht, die das Auffinden der Daten ermöglichen, und der für die Erteilung der Auskunft erforderliche Aufwand nicht außer Verhältnis zu dem vom Betroffenen geltend gemachten Informationsinteresse steht. Die verantwortliche Stelle bestimmt das Verfahren, insbesondere die Form der Auskunftserteilung, nach pflichtgemäßem Ermessen.

(2) Absatz 1 gilt nicht für personenbezogene Daten, die nur deshalb gespeichert sind, weil sie aufgrund gesetzlicher, satzungsmäßiger oder vertraglicher Aufbewahrungsvorschriften nicht gelöscht werden dürfen, oder ausschließlich Zwecken der Datensicherung oder der Datenschutzkontrolle dienen und eine Auskunftserteilung einen unverhältnismäßigen Aufwand erfordern würde.

(3) Bezieht sich die Auskunftserteilung auf die Übermittlung personenbezogener Daten an Verfassungsschutzbehörden, den Bundesnachrichtendienst, den Militärischen Abschirmdienst und, soweit die Sicherheit des Bundes berührt wird, andere Behörden des Bundesministeriums der Verteidigung, ist sie nur mit Zustimmung dieser Stellen zulässig.

(4) Die Auskunftserteilung unterbleibt, soweit

1. die Auskunft die ordnungsgemäße Erfüllung der in der Zuständigkeit der verantwortlichen Stelle liegenden Aufgaben gefährden würde,
2. die Auskunft die öffentliche Sicherheit oder Ordnung gefährden oder sonst dem Wohle des Bundes oder eines Landes Nachteile bereiten würde oder
3. die Daten oder die Tatsache ihrer Speicherung nach einer Rechtsvorschrift oder ihrem Wesen nach, insbesondere wegen der überwiegenden berechtigten Interessen eines Dritten, geheim gehalten werden müssen

und deswegen das Interesse des Betroffenen an der Auskunftserteilung zurücktreten muss.

(5) Die Ablehnung der Auskunftserteilung bedarf einer Begründung nicht, soweit durch die Mitteilung der tatsächlichen und rechtlichen Gründe, auf die die Entscheidung gestützt wird, der mit der Auskunftsverweigerung verfolgte Zweck gefährdet würde. In diesem Fall ist der Betroffene darauf hinzuweisen, dass er sich an den Bundesbeauftragten für den Datenschutz und die Informationsfreiheit wenden kann.

(6) Wird dem Betroffenen keine Auskunft erteilt, so ist sie auf sein Verlangen dem Bundesbeauftragten für den Datenschutz und die Informationsfreiheit zu erteilen, soweit nicht die jeweils zuständige oberste Bundesbehörde im Einzelfall feststellt, dass dadurch die Sicherheit des Bundes oder eines Landes gefährdet würde. Die Mitteilung des Bundesbeauftragten an den Betroffenen darf keine Rückschlüsse auf den Erkenntnisstand der verantwortlichen Stelle zulassen, sofern diese nicht einer weitergehenden Auskunft zustimmt.

(7) Die Auskunft ist unentgeltlich.

§ 19a Benachrichtigung

(1) Werden Daten ohne Kenntnis des Betroffenen erhoben, so ist er von der Speicherung, der Identität der verantwortlichen Stelle sowie über die Zweckbestimmungen der Erhebung, Verarbeitung oder Nutzung zu unterrichten. Der Betroffene ist auch über die Empfänger oder Kategorien von Empfängern von Daten zu unterrichten, soweit er nicht mit der Übermittlung an diese rechnen muss. Sofern eine Übermittlung vorgesehen ist, hat die Unterrichtung spätestens bei der ersten Übermittlung zu erfolgen.

(2) Eine Pflicht zur Benachrichtigung besteht nicht, wenn

1. der Betroffene auf andere Weise Kenntnis von der Speicherung oder der Übermittlung erlangt hat,
2. die Unterrichtung des Betroffenen einen unverhältnismäßigen Aufwand erfordert oder
3. die Speicherung oder Übermittlung der personenbezogenen Daten durch Gesetz ausdrücklich vorgesehen ist.

Die verantwortliche Stelle legt schriftlich fest, unter welchen Voraussetzungen von einer Benachrichtigung nach Nummer 2 oder 3 abgesehen wird.

(3) § 19 Abs. 2 bis 4 gilt entsprechend.

§ 20 Berichtigung, Löschung und Sperrung von Daten; Widerspruchsrecht

(1) Personenbezogene Daten sind zu berichtigen, wenn sie unrichtig sind. Wird festgestellt, dass personenbezogene Daten, die weder automatisiert verarbeitet noch in nicht automatisierten Dateien gespeichert sind, unrichtig sind, oder wird ihre Richtigkeit von dem Betroffenen bestritten, so ist dies in geeigneter Weise festzuhalten.

(2) Personenbezogene Daten, die automatisiert verarbeitet oder in nicht automatisierten Dateien gespeichert sind, sind zu löschen, wenn

1. ihre Speicherung unzulässig ist oder
2. ihre Kenntnis für die verantwortliche Stelle zur Erfüllung der in ihrer Zuständigkeit liegenden Aufgaben nicht mehr erforderlich ist.

(3) An die Stelle einer Löschung tritt eine Sperrung, soweit

1. einer Löschung gesetzliche, satzungsmäßige oder vertragliche Aufbewahrungsfristen entgegenstehen,
2. Grund zu der Annahme besteht, dass durch eine Löschung schutzwürdige Interessen des Betroffenen beeinträchtigt würden, oder
3. eine Löschung wegen der besonderen Art der Speicherung nicht oder nur mit unverhältnismäßig hohem Aufwand möglich ist.

(4) Personenbezogene Daten, die automatisiert verarbeitet oder in nicht automatisierten Dateien gespeichert sind, sind ferner zu sperren, soweit ihre Richtigkeit vom Betroffenen bestritten wird und sich weder die Richtigkeit noch die Unrichtigkeit feststellen lässt.

(5) Personenbezogene Daten dürfen nicht für eine automatisierte Verarbeitung oder Verarbeitung in nicht automatisierten Dateien erhoben, verarbeitet oder genutzt werden, soweit der Betroffene dieser bei der verantwortlichen Stelle widerspricht und eine Prüfung ergibt, dass das schutzwürdige Interesse des Betroffenen wegen seiner besonderen persönlichen Situation das Interesse der verantwortlichen Stelle an dieser Erhebung, Verarbeitung oder Nutzung überwiegt. Satz 1 gilt nicht, wenn eine Rechtsvorschrift zur Erhebung, Verarbeitung oder Nutzung verpflichtet.

(6) Personenbezogene Daten, die weder automatisiert verarbeitet noch in einer nicht automatisierten Datei gespeichert sind, sind zu sperren, wenn die Behörde im Einzelfall feststellt, dass ohne die Sperrung schutzwürdige Interessen des Betroffenen beeinträchtigt würden und die Daten für die Aufgabenerfüllung der Behörde nicht mehr erforderlich sind.

(7) Gesperrte Daten dürfen ohne Einwilligung des Betroffenen nur übermittelt oder genutzt werden, wenn

1. es zu wissenschaftlichen Zwecken, zur Behebung einer bestehenden Beweisnot oder aus sonstigen im überwiegenden Interesse der verantwortlichen Stelle oder eines Dritten liegenden Gründen unerlässlich ist und
2. die Daten hierfür übermittelt oder genutzt werden dürften, wenn sie nicht gesperrt wären.

(8) Von der Berichtigung unrichtiger Daten, der Sperrung bestrittener Daten sowie der Löschung oder Sperrung wegen Unzulässigkeit der Speicherung sind die Stellen zu verständigen, denen im Rahmen einer Datenübermittlung diese Daten zur Speicherung weitergegeben wurden, wenn dies keinen unverhältnismäßigen Aufwand erfordert und schutzwürdige Interessen des Betroffenen nicht entgegenstehen.

(9) § 2 Abs. 1 bis 6, 8 und 9 des Bundesarchivgesetzes ist anzuwenden.

§ 21 Anrufung des Bundesbeauftragten für den Datenschutz und die Informationsfreiheit

Jedermann kann sich an den Bundesbeauftragten für den Datenschutz und die Informationsfreiheit wenden, wenn er der Ansicht ist, bei der Erhebung, Verarbeitung oder Nutzung seiner personenbezogenen Daten durch öffentliche Stellen des Bundes in seinen Rechten verletzt worden zu sein. Für die Erhebung, Verarbeitung oder Nutzung von personenbezogenen Daten durch Gerichte des Bundes gilt dies nur, soweit diese in Verwaltungsangelegenheiten tätig werden.

Dritter Unterabschnitt
Bundesbeauftragter für den Datenschutz und die Informationsfreiheit

§ 22 Wahl des Bundesbeauftragten für den Datenschutz und die Informationsfreiheit

(1) Der Deutsche Bundestag wählt auf Vorschlag der Bundesregierung den Bundesbeauftragten für den Datenschutz und die Informationsfreiheit mit mehr als der Hälfte der gesetzlichen Zahl seiner Mitglieder. Der Bundesbeauftragte muss bei seiner Wahl das 35. Lebensjahr vollendet haben. Der Gewählte ist vom Bundespräsidenten zu ernennen.

(2) Der Bundesbeauftragte leistet vor dem Bundesminister des Innern folgenden Eid:

> "Ich schwöre, dass ich meine Kraft dem Wohle des deutschen Volkes widmen, seinen Nutzen mehren, Schaden von ihm wenden, das Grundgesetz und die Gesetze des Bundes wahren und verteidigen, meine Pflichten gewissenhaft erfüllen und Gerechtigkeit gegen jedermann üben werde. So wahr mir Gott helfe."

Der Eid kann auch ohne religiöse Beteuerung geleistet werden.

(3) Die Amtszeit des Bundesbeauftragten beträgt fünf Jahre. Einmalige Wiederwahl ist zulässig.

(4) Der Bundesbeauftragte steht nach Maßgabe dieses Gesetzes zum Bund in einem öffentlich-rechtlichen Amtsverhältnis. Er ist in Ausübung seines Amtes unabhängig und nur dem Gesetz unterworfen. Er untersteht der Rechtsaufsicht der Bundesregierung.

(5) Der Bundesbeauftragte wird beim Bundesministerium des Innern eingerichtet. Er untersteht der Dienstaufsicht des Bundesministeriums des Innern. Dem Bundesbeauftragten ist die für die Erfüllung seiner Aufgaben notwendige Personal- und Sachausstattung zur Verfügung zu stellen; sie ist im Einzelplan des Bundesministeriums des Innern in einem eigenen Kapitel auszuweisen. Die Stellen sind im Einvernehmen mit dem Bundesbeauftragten zu besetzen. Die Mitarbeiter können, falls sie mit der beabsichtigten Maßnahme nicht einverstanden sind, nur im Einvernehmen mit ihm versetzt, abgeordnet oder umgesetzt werden.

(6) Ist der Bundesbeauftragte vorübergehend an der Ausübung seines Amtes verhindert, kann der Bundesminister des Innern einen Vertreter mit der Wahrnehmung der Geschäfte beauftragen. Der Bundesbeauftragte soll dazu gehört werden.

§ 23 Rechtsstellung des Bundesbeauftragten für den Datenschutz und die Informationsfreiheit

(1) Das Amtsverhältnis des Bundesbeauftragten für den Datenschutz und die Informationsfreiheit beginnt mit der Aushändigung der Ernennungsurkunde. Es endet

1. mit Ablauf der Amtszeit,
2. mit der Entlassung.

Der Bundespräsident entlässt den Bundesbeauftragten, wenn dieser es verlangt oder auf Vorschlag der Bundesregierung, wenn Gründe vorliegen, die bei einem Richter auf Lebenszeit die Entlassung aus dem Dienst rechtfertigen. Im Falle der Beendigung des Amtsverhältnisses erhält der Bundesbeauftragte eine vom Bundespräsidenten vollzogene Urkunde. Eine Entlassung wird mit der Aushändigung der Urkunde wirksam. Auf Ersuchen des Bundesministers des Innern ist der Bundesbeauftragte verpflichtet, die Geschäfte bis zur Ernennung seines Nachfolgers weiterzuführen.

(2) Der Bundesbeauftragte darf neben seinem Amt kein anderes besoldetes Amt, kein Gewerbe und keinen Beruf ausüben und weder der Leitung oder dem Aufsichtsrat oder Verwaltungsrat eines auf Erwerb gerichteten Unternehmens noch einer Regierung oder einer gesetzgebenden Körperschaft des Bundes oder eines Landes angehören. Er darf nicht gegen Entgelt außergerichtliche Gutachten abgeben.

(3) Der Bundesbeauftragte hat dem Bundesministerium des Innern Mitteilung über Geschenke zu machen, die er in Bezug auf sein Amt erhält. Das Bundesministerium des Innern entscheidet über die Verwendung der Geschenke.

(4) Der Bundesbeauftragte ist berechtigt, über Personen, die ihm in seiner Eigenschaft als Bundesbeauftragter Tatsachen anvertraut haben, sowie über diese Tatsachen selbst das Zeugnis zu verweigern. Dies gilt auch für die Mitarbeiter des Bundesbeauftragten mit der Maßgabe, dass über die Ausübung dieses Rechts der Bundesbeauftragte entscheidet. Soweit das Zeugnisverweigerungsrecht des Bundesbeauftragten reicht, darf die Vorlegung oder Auslieferung von Akten oder anderen Schriftstücken von ihm nicht gefordert werden.

(5) Der Bundesbeauftragte ist, auch nach Beendigung seines Amtsverhältnisses, verpflichtet, über die ihm amtlich bekanntgewordenen Angelegenheiten Verschwiegenheit zu bewahren. Dies gilt nicht für Mitteilungen im dienstlichen Verkehr oder über Tatsachen, die offenkundig sind oder ihrer Bedeutung nach keiner Geheimhaltung bedürfen. Der Bundesbeauftragte darf, auch wenn er nicht mehr im Amt ist, über solche Angelegenheiten ohne Genehmigung des Bundesministeriums des Innern weder vor Gericht noch außergerichtlich aussagen oder Erklärungen abgeben. Unberührt bleibt die gesetzlich begründete Pflicht, Straftaten anzuzeigen und bei Gefährdung der freiheitlichen demokratischen Grundordnung für deren Erhaltung einzutreten. Für den Bundesbeauftragten und seine Mitarbeiter gelten die §§ 93, 97, 105 Abs. 1, § 111 Abs. 5 in Verbindung mit § 105 Abs. 1 sowie § 116 Abs. 1 der Abgabenordnung nicht. Satz 5 findet keine Anwendung, soweit die Finanzbehörden die Kenntnis für die Durchführung eines Verfahrens wegen einer Steuerstraftat sowie eines damit zusammenhängenden Steuerverfahrens benötigen, an deren Verfolgung ein zwingendes öffentliches Interesse besteht, oder soweit es sich um vorsätzlich falsche Angaben des Auskunftspflichtigen oder der für ihn tätigen Personen handelt. Stellt der Bundesbeauftragte einen Datenschutzverstoß fest, ist er befugt, diesen anzuzeigen und den Betroffenen hierüber zu informieren.

(6) Die Genehmigung, als Zeuge auszusagen, soll nur versagt werden, wenn die Aussage dem Wohle des Bundes oder eines deutschen Landes Nachteile bereiten oder die Erfüllung öffentlicher Aufgaben ernstlich gefährden oder erheblich erschweren würde. Die Genehmigung, ein Gutachten zu erstatten, kann versagt werden, wenn die Erstattung den dienstlichen Interessen Nachteile bereiten würde. § 28 des Bundesverfassungsgerichtsgesetzes bleibt unberührt.

(7) Der Bundesbeauftragte erhält vom Beginn des Kalendermonats an, in dem das Amtsverhältnis beginnt, bis zum Schluss des Kalendermonats, in dem das Amtsverhältnis endet, im Falle des Absatzes 1 Satz 6 bis zum Ende des Monats, in dem die Geschäftsführung endet, Amtsbezüge in Höhe der einem Bundesbeamten der Besoldungsgruppe B 9 zustehenden Besoldung. Das Bundesreisekostengesetz und das Bundesumzugskostengesetz sind entsprechend anzuwenden. Im Übrigen sind § 12 Abs 6 sowie die §§ 13 bis 20 und 21a Abs. 5 des Bundesministergesetzes mit den Maßgaben anzuwenden, dass an die Stelle der vierjährigen Amtszeit in § 15 Abs. 1 des Bundesministergesetzes eine Amtszeit von fünf Jahren und an die Stelle der Besoldungsgruppe B 11 in § 21a Abs. 5 des Bundesministergesetzes die Besoldungsgruppe B 9 tritt. Abweichend von Satz 3 in Verbindung mit den §§ 15 bis 17 und 21a Abs. 5 des Bundesministergesetzes berechnet sich das Ruhegehalt des Bundesbeauftragten unter Hinzurechnung der Amtszeit als ruhegehaltsfähige Dienstzeit in entsprechender Anwendung des Beamtenversorgungsgesetzes, wenn dies günstiger ist und der Bundesbeauftragte sich unmittelbar vor seiner Wahl zum Bundesbeauftragten als Beamter oder Richter mindestens in dem letzten gewöhnlich vor Erreichen der Besoldungsgruppe B 9 zu durchlaufenden Amt befunden hat.

(8) Absatz 5 Satz 5 bis 7 gilt entsprechend für die öffentlichen Stellen, die für die Kontrolle der Einhaltung der Vorschriften über den Datenschutz in den Ländern zuständig sind.

§ 24 Kontrolle durch den Bundesbeauftragten für den Datenschutz und die Informationsfreiheit

(1) Der Bundesbeauftragte für den Datenschutz und die Informationsfreiheit kontrolliert bei den öffentlichen Stellen des Bundes die Einhaltung der Vorschriften dieses Gesetzes und anderer Vorschriften über den Datenschutz.

(2) Die Kontrolle des Bundesbeauftragten erstreckt sich auch auf

1. von öffentlichen Stellen des Bundes erlangte personenbezogene Daten über den Inhalt und die näheren Umstände des Brief-, Post- und Fernmeldeverkehrs, und
2. personenbezogene Daten, die einem Berufs- oder besonderen Amtsgeheimnis, insbesondere dem Steuergeheimnis nach § 30 der Abgabenordnung, unterliegen.

Das Grundrecht des Brief-, Post- und Fernmeldegeheimnisses des Artikels 10 des Grundgesetzes wird insoweit eingeschränkt. Personenbezogene Daten, die der Kontrolle durch die Kommission nach § 15 des Artikel 10-Gesetzes unterliegen, unterliegen nicht der Kontrolle durch den Bundesbeauftragten, es sei denn, die

Kommission ersucht den Bundesbeauftragten, die Einhaltung der Vorschriften über den Datenschutz bei bestimmten Vorgängen oder in bestimmten Bereichen zu kontrollieren und ausschließlich ihr darüber zu berichten. Der Kontrolle durch den Bundesbeauftragten unterliegen auch nicht personenbezogene Daten in Akten über die Sicherheitsüberprüfung, wenn der Betroffene der Kontrolle der auf ihn bezogenen Daten im Einzelfall gegenüber dem Bundesbeauftragten widerspricht.

(3) Die Bundesgerichte unterliegen der Kontrolle des Bundesbeauftragten nur, soweit sie in Verwaltungsangelegenheiten tätig werden.

(4) Die öffentlichen Stellen des Bundes sind verpflichtet, den Bundesbeauftragten und seine Beauftragten bei der Erfüllung ihrer Aufgaben zu unterstützen. Ihnen ist dabei insbesondere

1. Auskunft zu ihren Fragen sowie Einsicht in alle Unterlagen, insbesondere in die gespeicherten Daten und in die Datenverarbeitungsprogramme, zu gewähren, die im Zusammenhang mit der Kontrolle nach Absatz 1 stehen,
2. jederzeit Zutritt in alle Diensträume zu gewähren.

Die in § 6 Abs. 2 und § 19 Abs. 3 genannten Behörden gewähren die Unterstützung nur dem Bundesbeauftragten selbst und den von ihm schriftlich besonders Beauftragten. Satz 2 gilt für diese Behörden nicht, soweit die oberste Bundesbehörde im Einzelfall feststellt, dass die Auskunft oder Einsicht die Sicherheit des Bundes oder eines Landes gefährden würde.

(5) Der Bundesbeauftragte teilt das Ergebnis seiner Kontrolle der öffentlichen Stelle mit. Damit kann er Vorschläge zur Verbesserung des Datenschutzes, insbesondere zur Beseitigung von festgestellten Mängeln bei der Verarbeitung oder Nutzung personenbezogener Daten, verbinden. § 25 bleibt unberührt.

(6) Absatz 2 gilt entsprechend für die öffentlichen Stellen, die für die Kontrolle der Einhaltung der Vorschriften über den Datenschutz in den Ländern zuständig sind.

§ 25 Beanstandungen durch den Bundesbeauftragten für den Datenschutz und die Informationsfreiheit

(1) Stellt der Bundesbeauftragte für den Datenschutz und die Informationsfreiheit Verstöße gegen die Vorschriften dieses Gesetzes oder gegen andere Vorschriften über den Datenschutz oder sonstige Mängel bei der Verarbeitung oder Nutzung personenbezogener Daten fest, so beanstandet er dies

1. bei der Bundesverwaltung gegenüber der zuständigen obersten Bundesbehörde,
2. beim Bundeseisenbahnvermögen gegenüber dem Präsidenten,
3. bei den aus dem Sondervermögen Deutsche Bundespost durch Gesetz hervorgegangenen Unternehmen, solange ihnen ein ausschließliches Recht nach dem Postgesetz zusteht, gegenüber deren Vorständen,
4. bei den bundesunmittelbaren Körperschaften, Anstalten und Stiftungen des öffentlichen Rechts sowie bei Vereinigungen solcher Körperschaften, Anstalten und Stiftungen gegenüber dem Vorstand oder dem sonst vertretungsberechtigten Organ

und fordert zur Stellungnahme innerhalb einer von ihm zu bestimmenden Frist auf. In den Fällen von Satz 1 Nr. 4 unterrichtet der Bundesbeauftragte gleichzeitig die zuständige Aufsichtsbehörde.

(2) Der Bundesbeauftragte kann von einer Beanstandung absehen oder auf eine Stellungnahme der betroffenen Stelle verzichten, insbesondere wenn es sich um unerhebliche oder inzwischen beseitigte Mängel handelt.

(3) Die Stellungnahme soll auch eine Darstellung der Maßnahmen enthalten, die aufgrund der Beanstandung des Bundesbeauftragten getroffen worden sind. Die in Absatz 1 Satz 1 Nr. 4 genannten Stellen leiten der zuständigen Aufsichtsbehörde gleichzeitig eine Abschrift ihrer Stellungnahme an den Bundesbeauftragten zu.

§ 26 Weitere Aufgaben des Bundesbeauftragten für den Datenschutz und die Informationsfreiheit

(1) Der Bundesbeauftragte für den Datenschutz und die Informationsfreiheit erstattet dem Deutschen Bundestag alle zwei Jahre einen Tätigkeitsbericht. Er unterrichtet den Deutschen Bundestag und die Öffentlichkeit über wesentliche Entwicklungen des Datenschutzes.

(2) Auf Anforderung des Deutschen Bundestages oder der Bundesregierung hat der Bundesbeauftragte Gutachten zu erstellen und Berichte zu erstatten. Auf Ersuchen des Deutschen Bundestages, des

Petitionsausschusses, des Innenausschusses oder der Bundesregierung geht der Bundesbeauftragte ferner Hinweisen auf Angelegenheiten und Vorgänge des Datenschutzes bei den öffentlichen Stellen des Bundes nach. Der Bundesbeauftragte kann sich jederzeit an den Deutschen Bundestag wenden.

(3) Der Bundesbeauftragte kann der Bundesregierung und den in § 12 Abs. 1 genannten Stellen des Bundes Empfehlungen zur Verbesserung des Datenschutzes geben und sie in Fragen des Datenschutzes beraten. Die in § 25 Abs. 1 Nr. 1 bis 4 genannten Stellen sind durch den Bundesbeauftragten zu unterrichten, wenn die Empfehlung oder Beratung sie nicht unmittelbar betrifft.

(4) Der Bundesbeauftragte wirkt auf die Zusammenarbeit mit den öffentlichen Stellen, die für die Kontrolle der Einhaltung der Vorschriften über den Datenschutz in den Ländern zuständig sind, sowie mit den Aufsichtsbehörden nach § 38 hin. § 38 Abs. 1 Satz 4 und 5 gilt entsprechend.

Dritter Abschnitt
Datenverarbeitung nicht-öffentlicher Stellen und öffentlich-rechtlicher Wettbewerbsunternehmen

Erster Unterabschnitt
Rechtsgrundlagen der Datenverarbeitung

§ 27 Anwendungsbereich

(1) Die Vorschriften dieses Abschnittes finden Anwendung, soweit personenbezogene Daten unter Einsatz von Datenverarbeitungsanlagen verarbeitet, genutzt oder dafür erhoben werden oder die Daten in oder aus nicht automatisierten Dateien verarbeitet, genutzt oder dafür erhoben werden durch

1. nicht-öffentliche Stellen,
2.
   a) öffentliche Stellen des Bundes, soweit sie als öffentlich-rechtliche Unternehmen am Wettbewerb teilnehmen,

   b) öffentliche Stellen der Länder, soweit sie als öffentlich-rechtliche Unternehmen am Wettbewerb teilnehmen, Bundesrecht ausführen und der Datenschutz nicht durch Landesgesetz geregelt ist.

Dies gilt nicht, wenn die Erhebung, Verarbeitung oder Nutzung der Daten ausschließlich für persönliche oder familiäre Tätigkeiten erfolgt. In den Fällen der Nummer 2 Buchstabe a gelten anstelle des § 38 die §§ 18, 21 und 24 bis 26.

(2) Die Vorschriften dieses Abschnittes gelten nicht für die Verarbeitung und Nutzung personenbezogener Daten außerhalb von nicht automatisierten Dateien, soweit es sich nicht um personenbezogene Daten handelt, die offensichtlich aus einer automatisierten Verarbeitung entnommen worden sind.

§ 28 Datenerhebung und -speicherung für eigene Geschäftszwecke

(1) Das Erheben, Speichern, Verändern oder Übermitteln personenbezogener Daten oder ihre Nutzung als Mittel für die Erfüllung eigener Geschäftszwecke ist zulässig

1. wenn es für die Begründung, Durchführung oder Beendigung eines rechtsgeschäftlichen oder rechtsgeschäftsähnlichen Schuldverhältnisses mit dem Betroffenen erforderlich ist,
2. soweit es zur Wahrung berechtigter Interessen der verantwortlichen Stelle erforderlich ist und kein Grund zu der Annahme besteht, dass das schutzwürdige Interesse des Betroffenen an dem Ausschluss der Verarbeitung oder Nutzung überwiegt, oder
3. wenn die Daten allgemein zugänglich sind oder die verantwortliche Stelle sie veröffentlichen dürfte, es sei denn, dass das schutzwürdige Interesse des Betroffenen an dem Ausschluss der Verarbeitung oder Nutzung gegenüber dem berechtigten Interesse der verantwortlichen Stelle offensichtlich überwiegt.

Bei der Erhebung personenbezogener Daten sind die Zwecke, für die die Daten verarbeitet oder genutzt werden sollen, konkret festzulegen.

(2) Die Übermittlung oder Nutzung für einen anderen Zweck ist zulässig

1. unter den Voraussetzungen des Absatzes 1 Satz 1 Nummer 2 oder Nummer 3,

2. soweit es erforderlich ist,
   a) zur Wahrung berechtigter Interessen eines Dritten oder
   b) zur Abwehr von Gefahren für die staatliche oder öffentliche Sicherheit oder zur Verfolgung von Straftaten

   und kein Grund zu der Annahme besteht, dass der Betroffene ein schutzwürdiges Interesse an dem Ausschluss der Übermittlung oder Nutzung hat, oder
3. wenn es im Interesse einer Forschungseinrichtung zur Durchführung wissenschaftlicher Forschung erforderlich ist, das wissenschaftliche Interesse an der Durchführung des Forschungsvorhabens das Interesse des Betroffenen an dem Ausschluss der Zweckänderung erheblich überwiegt und der Zweck der Forschung auf andere Weise nicht oder nur mit unverhältnismäßigem Aufwand erreicht werden kann.

(3) Die Verarbeitung oder Nutzung personenbezogener Daten für Zwecke des Adresshandels oder der Werbung ist zulässig, soweit der Betroffene eingewilligt hat und im Falle einer nicht schriftlich erteilten Einwilligung die verantwortliche Stelle nach Absatz 3a verfährt. Darüber hinaus ist die Verarbeitung oder Nutzung personenbezogener Daten zulässig, soweit es sich um listenmäßig oder sonst zusammengefasste Daten über Angehörige einer Personengruppe handelt, die sich auf die Zugehörigkeit des Betroffenen zu dieser Personengruppe, seine Berufs-, Branchen- oder Geschäftsbezeichnung, seinen Namen, Titel, akademischen Grad, seine Anschrift und sein Geburtsjahr beschränken, und die Verarbeitung oder Nutzung erforderlich ist

1. für Zwecke der Werbung für eigene Angebote der verantwortlichen Stelle, die diese Daten mit Ausnahme der Angaben zur Gruppenzugehörigkeit beim Betroffenen nach Absatz 1 Satz 1 Nummer 1 oder aus allgemein zugänglichen Adress-, Rufnummern-, Branchen- oder vergleichbaren Verzeichnissen erhoben hat,
2. für Zwecke der Werbung im Hinblick auf die berufliche Tätigkeit des Betroffenen und unter seiner beruflichen Anschrift oder
3. für Zwecke der Werbung für Spenden, die nach § 10b Absatz 1 und § 34g des Einkommensteuergesetzes steuerbegünstigt sind.

Für Zwecke nach Satz 2 Nummer 1 darf die verantwortliche Stelle zu den dort genannten Daten weitere Daten hinzuspeichern. Zusammengefasste personenbezogene Daten nach Satz 2 dürfen auch dann für Zwecke der Werbung übermittelt werden, wenn die Übermittlung nach Maßgabe des § 34 Absatz 1a Satz 1 gespeichert wird; in diesem Fall muss die Stelle, die die Daten erstmalig erhoben hat, aus der Werbung eindeutig hervorgehen. Unabhängig vom Vorliegen der Voraussetzungen des Satzes 2 dürfen personenbezogene Daten für Zwecke der Werbung für fremde Angebote genutzt werden, wenn für den Betroffenen bei der Ansprache zum Zwecke der Werbung die für die Nutzung der Daten verantwortliche Stelle eindeutig erkennbar ist. Eine Verarbeitung oder Nutzung nach den Sätzen 2 bis 4 ist nur zulässig, soweit schutzwürdige Interessen des Betroffenen nicht entgegenstehen. Nach den Sätzen 1, 2 und 4 übermittelte Daten dürfen nur für den Zweck verarbeitet oder genutzt werden, für den sie übermittelt worden sind.

(3a) Wird die Einwilligung nach § 4a Absatz 1 Satz 3 in anderer Form als der Schriftform erteilt, hat die verantwortliche Stelle dem Betroffenen den Inhalt der Einwilligung schriftlich zu bestätigen, es sei denn, dass die Einwilligung elektronisch erklärt wird und die verantwortliche Stelle sicherstellt, dass die Einwilligung protokolliert wird und der Betroffene deren Inhalt jederzeit abrufen und die Einwilligung jederzeit mit Wirkung für die Zukunft widerrufen kann. Soll die Einwilligung zusammen mit anderen Erklärungen schriftlich erteilt werden, ist sie in drucktechnisch deutlicher Gestaltung besonders hervorzuheben.

(3b) Die verantwortliche Stelle darf den Abschluss eines Vertrags nicht von einer Einwilligung des Betroffenen nach Absatz 3 Satz 1 abhängig machen, wenn dem Betroffenen ein anderer Zugang zu gleichwertigen vertraglichen Leistungen ohne die Einwilligung nicht oder nicht in zumutbarer Weise möglich ist. Eine unter solchen Umständen erteilte Einwilligung ist unwirksam.

(4) Widerspricht der Betroffene bei der verantwortlichen Stelle der Verarbeitung oder Nutzung seiner Daten für Zwecke der Werbung oder der Markt- oder Meinungsforschung, ist eine Verarbeitung oder Nutzung für diese Zwecke unzulässig. Der Betroffene ist bei der Ansprache zum Zweck der Werbung oder der Markt- oder Meinungsforschung und in den Fällen des Absatzes 1 Satz 1 Nummer 1 auch bei Begründung des rechtsgeschäftlichen oder rechtsgeschäftsähnlichen Schuldverhältnisses über die verantwortliche Stelle sowie über das Widerspruchsrecht nach Satz 1 zu unterrichten; soweit der Ansprechende personenbezogene Daten des Betroffenen nutzt, die bei einer ihm nicht bekannten Stelle gespeichert sind, hat er auch sicherzustellen, dass der Betroffene Kenntnis über die Herkunft der Daten erhalten kann. Widerspricht der Betroffene bei dem Dritten, dem die Daten im Rahmen der Zwecke nach Absatz 3 übermittelt worden sind, der Verarbeitung oder Nutzung für Zwecke der Werbung oder der Markt- oder Meinungsforschung, hat dieser die Daten für diese Zwecke zu sperren.

In den Fällen des Absatzes 1 Satz 1 Nummer 1 darf für den Widerspruch keine strengere Form verlangt werden als für die Begründung des rechtsgeschäftlichen oder rechtsgeschäftsähnlichen Schuldverhältnisses.

(5) Der Dritte, dem die Daten übermittelt worden sind, darf diese nur für den Zweck verarbeiten oder nutzen, zu dessen Erfüllung sie ihm übermittelt werden. Eine Verarbeitung oder Nutzung für andere Zwecke ist nicht-öffentlichen Stellen nur unter den Voraussetzungen der Absätze 2 und 3 und öffentlichen Stellen nur unter den Voraussetzungen des § 14 Abs. 2 erlaubt. Die übermittelnde Stelle hat ihn darauf hinzuweisen.

(6) Das Erheben, Verarbeiten und Nutzen von besonderen Arten personenbezogener Daten (§ 3 Abs. 9) für eigene Geschäftszwecke ist zulässig, soweit nicht der Betroffene nach Maßgabe des § 4a Abs. 3 eingewilligt hat, wenn

1. dies zum Schutz lebenswichtiger Interessen des Betroffenen oder eines Dritten erforderlich ist, sofern der Betroffene aus physischen oder rechtlichen Gründen außerstande ist, seine Einwilligung zu geben,
2. es sich um Daten handelt, die der Betroffene offenkundig öffentlich gemacht hat,
3. dies zur Geltendmachung, Ausübung oder Verteidigung rechtlicher Ansprüche erforderlich ist und kein Grund zu der Annahme besteht, dass das schutzwürdige Interesse des Betroffenen an dem Ausschluss der Erhebung, Verarbeitung oder Nutzung überwiegt, oder
4. dies zur Durchführung wissenschaftlicher Forschung erforderlich ist, das wissenschaftliche Interesse an der Durchführung des Forschungsvorhabens das Interesse des Betroffenen an dem Ausschluss der Erhebung, Verarbeitung und Nutzung erheblich überwiegt und der Zweck der Forschung auf andere Weise nicht oder nur mit unverhältnismäßigem Aufwand erreicht werden kann.

(7) Das Erheben von besonderen Arten personenbezogener Daten (§ 3 Abs. 9) ist ferner zulässig, wenn dies zum Zweck der Gesundheitsvorsorge, der medizinischen Diagnostik, der Gesundheitsversorgung oder Behandlung oder für die Verwaltung von Gesundheitsdiensten erforderlich ist und die Verarbeitung dieser Daten durch ärztliches Personal oder durch sonstige Personen erfolgt, die einer entsprechenden Geheimhaltungspflicht unterliegen. Die Verarbeitung und Nutzung von Daten zu den in Satz 1 genannten Zwecken richtet sich nach den für die in Satz 1 genannten Personen geltenden Geheimhaltungspflichten. Werden zu einem in Satz 1 genannten Zweck Daten über die Gesundheit von Personen durch Angehörige eines anderen als in § 203 Abs. 1 und 3 des Strafgesetzbuchs genannten Berufes, dessen Ausübung die Feststellung, Heilung oder Linderung von Krankheiten oder die Herstellung oder den Vertrieb von Hilfsmitteln mit sich bringt, erhoben, verarbeitet oder genutzt, ist dies nur unter den Voraussetzungen zulässig, unter denen ein Arzt selbst hierzu befugt wäre.

(8) Für einen anderen Zweck dürfen die besonderen Arten personenbezogener Daten (§ 3 Abs. 9) nur unter den Voraussetzungen des Absatzes 6 Nr. 1 bis 4 oder des Absatzes 7 Satz 1 übermittelt oder genutzt werden. Eine Übermittlung oder Nutzung ist auch zulässig, wenn dies zur Abwehr von erheblichen Gefahren für die staatliche und öffentliche Sicherheit sowie zur Verfolgung von Straftaten von erheblicher Bedeutung erforderlich ist.

(9) Organisationen, die politisch, philosophisch, religiös oder gewerkschaftlich ausgerichtet sind und keinen Erwerbszweck verfolgen, dürfen besondere Arten personenbezogener Daten (§ 3 Abs. 9) erheben, verarbeiten oder nutzen, soweit dies für die Tätigkeit der Organisation erforderlich ist. Dies gilt nur für personenbezogene Daten ihrer Mitglieder oder von Personen, die im Zusammenhang mit deren Tätigkeitszweck regelmäßig Kontakte mit ihr unterhalten. Die Übermittlung dieser personenbezogenen Daten an Personen oder Stellen außerhalb der Organisation ist nur unter den Voraussetzungen des § 4a Abs. 3 zulässig. Absatz 2 Nummer 2 Buchstabe b gilt entsprechend.

§ 28a Datenübermittlung an Auskunfteien

(1) Die Übermittlung personenbezogener Daten über eine Forderung an Auskunfteien ist nur zulässig, soweit die geschuldete Leistung trotz Fälligkeit nicht erbracht worden ist, die Übermittlung zur Wahrung berechtigter Interessen der verantwortlichen Stelle oder eines Dritten erforderlich ist und

1. die Forderung durch ein rechtskräftiges oder für vorläufig vollstreckbar erklärtes Urteil festgestellt worden ist oder ein Schuldtitel nach § 794 der Zivilprozessordnung vorliegt,
2. die Forderung nach § 178 der Insolvenzordnung festgestellt und nicht vom Schuldner im Prüfungstermin bestritten worden ist,
3. der Betroffene die Forderung ausdrücklich anerkannt hat,
4. a) der Betroffene nach Eintritt der Fälligkeit der Forderung mindestens zweimal schriftlich gemahnt worden ist,

b) zwischen der ersten Mahnung und der Übermittlung mindestens vier Wochen liegen,

c) die verantwortliche Stelle den Betroffenen rechtzeitig vor der Übermittlung der Angaben, jedoch frühestens bei der ersten Mahnung über die bevorstehende Übermittlung unterrichtet hat und

d) der Betroffene die Forderung nicht bestritten hat oder

5. das der Forderung zugrunde liegende Vertragsverhältnis aufgrund von Zahlungsrückständen fristlos gekündigt werden kann und die verantwortliche Stelle den Betroffenen über die bevorstehende Übermittlung unterrichtet hat.

Satz 1 gilt entsprechend, wenn die verantwortliche Stelle selbst die Daten nach § 29 verwendet.

(2) Zur zukünftigen Übermittlung nach § 29 Abs. 2 dürfen Kreditinstitute personenbezogene Daten über die Begründung, ordnungsgemäße Durchführung und Beendigung eines Vertragsverhältnisses betreffend ein Bankgeschäft nach § 1 Abs. 1 Satz 2 Nr. 2, 8 oder Nr. 9 des Kreditwesengesetzes an Auskunfteien übermitteln, es sei denn, dass das schutzwürdige Interesse des Betroffenen an dem Ausschluss der Übermittlung gegenüber dem Interesse der Auskunftei an der Kenntnis der Daten offensichtlich überwiegt. Der Betroffene ist vor Abschluss des Vertrages hierüber zu unterrichten. Satz 1 gilt nicht für Giroverträge, die die Einrichtung eines Kontos ohne Überziehungsmöglichkeit zum Gegenstand haben. Zur zukünftigen Übermittlung nach § 29 Abs. 2 ist die Übermittlung von Daten über Verhaltensweisen des Betroffenen, die im Rahmen eines vorvertraglichen Vertrauensverhältnisses der Herstellung von Markttransparenz dienen, an Auskunfteien auch mit Einwilligung des Betroffenen unzulässig.

(3) Nachträgliche Änderungen der einer Übermittlung nach Absatz 1 oder Absatz 2 zugrunde liegenden Tatsachen hat die verantwortliche Stelle der Auskunftei innerhalb von einem Monat nach Kenntniserlangung mitzuteilen, solange die ursprünglich übermittelten Daten bei der Auskunftei gespeichert sind. Die Auskunftei hat die übermittelnde Stelle über die Löschung der ursprünglich übermittelten Daten zu unterrichten.

§ 28b Scoring

Zum Zweck der Entscheidung über die Begründung, Durchführung oder Beendigung eines Vertragsverhältnisses mit dem Betroffenen darf ein Wahrscheinlichkeitswert für ein bestimmtes zukünftiges Verhalten des Betroffenen erhoben oder verwendet werden, wenn

1. die zur Berechnung des Wahrscheinlichkeitswerts genutzten Daten unter Zugrundelegung eines wissenschaftlich anerkannten mathematisch-statistischen Verfahrens nachweisbar für die Berechnung der Wahrscheinlichkeit des bestimmten Verhaltens erheblich sind,
2. im Fall der Berechnung des Wahrscheinlichkeitswerts durch eine Auskunftei die Voraussetzungen für eine Übermittlung der genutzten Daten nach § 29 und in allen anderen Fällen die Voraussetzungen einer zulässigen Nutzung der Daten nach § 28 vorliegen,
3. für die Berechnung des Wahrscheinlichkeitswerts nicht ausschließlich Anschriftendaten genutzt werden,
4. im Fall der Nutzung von Anschriftendaten der Betroffene vor Berechnung des Wahrscheinlichkeitswerts über die vorgesehene Nutzung dieser Daten unterrichtet worden ist; die Unterrichtung ist zu dokumentieren.

§ 29 Geschäftsmäßige Datenerhebung und -speicherung zum Zweck der Übermittlung

(1) Das geschäftsmäßige Erheben, Speichern, Verändern oder Nutzen personenbezogener Daten zum Zweck der Übermittlung, insbesondere wenn dies der Werbung, der Tätigkeit von Auskunfteien oder dem Adresshandel dient, ist zulässig, wenn

1. kein Grund zu der Annahme besteht, dass der Betroffene ein schutzwürdiges Interesse an dem Ausschluss der Erhebung, Speicherung oder Veränderung hat,
2. die Daten aus allgemein zugänglichen Quellen entnommen werden können oder die verantwortliche Stelle sie veröffentlichen dürfte, es sei denn, dass das schutzwürdige Interesse des Betroffenen an dem Ausschluss der Erhebung, Speicherung oder Veränderung offensichtlich überwiegt, oder
3. die Voraussetzungen des § 28a Abs. 1 oder Abs. 2 erfüllt sind; Daten im Sinne von § 28a Abs. 2 Satz 4 dürfen nicht erhoben oder gespeichert werden.

§ 28 Absatz 1 Satz 2 und Absatz 3 bis 3b ist anzuwenden.

(2) Die Übermittlung im Rahmen der Zwecke nach Absatz 1 ist zulässig, wenn

1. der Dritte, dem die Daten übermittelt werden, ein berechtigtes Interesse an ihrer Kenntnis glaubhaft dargelegt hat und
2. kein Grund zu der Annahme besteht, dass der Betroffene ein schutzwürdiges Interesse an dem Ausschluss der Übermittlung hat.

§ 28 Absatz 3 bis 3b gilt entsprechend. Bei der Übermittlung nach Satz 1 Nr. 1 sind die Gründe für das Vorliegen eines berechtigten Interesses und die Art und Weise ihrer glaubhaften Darlegung von der übermittelnden Stelle aufzuzeichnen. Bei der Übermittlung im automatisierten Abrufverfahren obliegt die Aufzeichnungspflicht dem Dritten, dem die Daten übermittelt werden. Die übermittelnde Stelle hat Stichprobenverfahren nach § 10 Abs. 4 Satz 3 durchzuführen und dabei auch das Vorliegen eines berechtigten Interesses einzelfallbezogen festzustellen und zu überprüfen.

(3) Die Aufnahme personenbezogener Daten in elektronische oder gedruckte Adress-, Rufnummern-, Branchen- oder vergleichbare Verzeichnisse hat zu unterbleiben, wenn der entgegenstehende Wille des Betroffenen aus dem zugrunde liegenden elektronischen oder gedruckten Verzeichnis oder Register ersichtlich ist. Der Empfänger der Daten hat sicherzustellen, dass Kennzeichnungen aus elektronischen oder gedruckten Verzeichnissen oder Registern bei der Übernahme in Verzeichnisse oder Register übernommen werden.

(4) Für die Verarbeitung oder Nutzung der übermittelten Daten gilt § 28 Abs. 4 und 5.

(5) § 28 Abs. 6 bis 9 gilt entsprechend.

(6) Eine Stelle, die geschäftsmäßig personenbezogene Daten, die zur Bewertung der Kreditwürdigkeit von Verbrauchern genutzt werden dürfen, zum Zweck der Übermittlung erhebt, speichert oder verändert, hat Auskunftsverlangen von Darlehensgebern aus anderen Mitgliedstaaten der Europäischen Union oder anderen Vertragsstaaten des Abkommens über den Europäischen Wirtschaftsraum genauso zu behandeln wie Auskunftsverlangen inländischer Darlehensgeber.

(7) Wer den Abschluss eines Verbraucherdarlehensvertrags oder eines Vertrags über eine entgeltliche Finanzierungshilfe mit einem Verbraucher infolge einer Auskunft einer Stelle im Sinne des Absatzes 6 ablehnt, hat den Verbraucher unverzüglich hierüber sowie über die erhaltene Auskunft zu unterrichten. Die Unterrichtung unterbleibt, soweit hierdurch die öffentliche Sicherheit oder Ordnung gefährdet würde. § 6a bleibt unberührt.

§ 30 Geschäftsmäßige Datenerhebung und -speicherung zum Zweck der Übermittlung in anonymisierter Form

(1) Werden personenbezogene Daten geschäftsmäßig erhoben und gespeichert, um sie in anonymisierter Form zu übermitteln, sind die Merkmale gesondert zu speichern, mit denen Einzelangaben über persönliche oder sachliche Verhältnisse einer bestimmten oder bestimmbaren natürlichen Person zugeordnet werden können. Diese Merkmale dürfen mit den Einzelangaben nur zusammengeführt werden, soweit dies für die Erfüllung des Zwecks der Speicherung oder zu wissenschaftlichen Zwecken erforderlich ist.

(2) Die Veränderung personenbezogener Daten ist zulässig, wenn

1. kein Grund zu der Annahme besteht, dass der Betroffene ein schutzwürdiges Interesse an dem Ausschluss der Veränderung hat, oder
2. die Daten aus allgemein zugänglichen Quellen entnommen werden können oder die verantwortliche Stelle sie veröffentlichen dürfte, soweit nicht das schutzwürdige Interesse des Betroffenen an dem Ausschluss der Veränderung offensichtlich überwiegt.

(3) Die personenbezogenen Daten sind zu löschen, wenn ihre Speicherung unzulässig ist.

(4) § 29 gilt nicht.

(5) § 28 Abs. 6 bis 9 gilt entsprechend.

§ 30a Geschäftsmäßige Datenerhebung und -speicherung für Zwecke der Markt- oder Meinungsforschung

(1) Das geschäftsmäßige Erheben, Verarbeiten oder Nutzen personenbezogener Daten für Zwecke der Markt- oder Meinungsforschung ist zulässig, wenn

1. kein Grund zu der Annahme besteht, dass der Betroffene ein schutzwürdiges Interesse an dem Ausschluss der Erhebung, Verarbeitung oder Nutzung hat, oder
2. die Daten aus allgemein zugänglichen Quellen entnommen werden können oder die verantwortliche Stelle sie veröffentlichen dürfte und das schutzwürdige Interesse des Betroffenen an dem Ausschluss der Erhebung, Verarbeitung oder Nutzung gegenüber dem Interesse der verantwortlichen Stelle nicht offensichtlich überwiegt.

Besondere Arten personenbezogener Daten (§ 3 Absatz 9) dürfen nur für ein bestimmtes Forschungsvorhaben erhoben, verarbeitet oder genutzt werden.

(2) Für Zwecke der Markt- oder Meinungsforschung erhobene oder gespeicherte personenbezogene Daten dürfen nur für diese Zwecke verarbeitet oder genutzt werden. Daten, die nicht aus allgemein zugänglichen Quellen entnommen worden sind und die die verantwortliche Stelle auch nicht veröffentlichen darf, dürfen nur für das Forschungsvorhaben verarbeitet oder genutzt werden, für das sie erhoben worden sind. Für einen anderen Zweck dürfen sie nur verarbeitet oder genutzt werden, wenn sie zuvor so anonymisiert werden, dass ein Personenbezug nicht mehr hergestellt werden kann.

(3) Die personenbezogenen Daten sind zu anonymisieren, sobald dies nach dem Zweck des Forschungsvorhabens, für das die Daten erhoben worden sind, möglich ist. Bis dahin sind die Merkmale gesondert zu speichern, mit denen Einzelangaben über persönliche oder sachliche Verhältnisse einer bestimmten oder bestimmbaren Person zugeordnet werden können. Diese Merkmale dürfen mit den Einzelangaben nur zusammengeführt werden, soweit dies nach dem Zweck des Forschungsvorhabens erforderlich ist.

(4) § 29 gilt nicht.

(5) § 28 Absatz 4 und 6 bis 9 gilt entsprechend.

§ 31 Besondere Zweckbindung

Personenbezogene Daten, die ausschließlich zu Zwecken der Datenschutzkontrolle, der Datensicherung oder zur Sicherstellung eines ordnungsgemäßen Betriebes einer Datenverarbeitungsanlage gespeichert werden, dürfen nur für diese Zwecke verwendet werden.

§ 32 Datenerhebung, -verarbeitung und -nutzung für Zwecke des Beschäftigungsverhältnisses

(1) Personenbezogene Daten eines Beschäftigten dürfen für Zwecke des Beschäftigungsverhältnisses erhoben, verarbeitet oder genutzt werden, wenn dies für die Entscheidung über die Begründung eines Beschäftigungsverhältnisses oder nach Begründung des Beschäftigungsverhältnisses für dessen Durchführung oder Beendigung erforderlich ist. Zur Aufdeckung von Straftaten dürfen personenbezogene Daten eines Beschäftigten nur dann erhoben, verarbeitet oder genutzt werden, wenn zu dokumentierende tatsächliche Anhaltspunkte den Verdacht begründen, dass der Betroffene im Beschäftigungsverhältnis eine Straftat begangen hat, die Erhebung, Verarbeitung oder Nutzung zur Aufdeckung erforderlich ist und das schutzwürdige Interesse des Beschäftigten an dem Ausschluss der Erhebung, Verarbeitung oder Nutzung nicht überwiegt, insbesondere Art und Ausmaß im Hinblick auf den Anlass nicht unverhältnismäßig sind.

(2) Absatz 1 ist auch anzuwenden, wenn personenbezogene Daten erhoben, verarbeitet oder genutzt werden, ohne dass sie automatisiert verarbeitet oder in oder aus einer nicht automatisierten Datei verarbeitet, genutzt oder für die Verarbeitung oder Nutzung in einer solchen Datei erhoben werden.

(3) Die Beteiligungsrechte der Interessenvertretungen der Beschäftigten bleiben unberührt.

Zweiter Unterabschnitt
Rechte des Betroffenen

§ 33 Benachrichtigung des Betroffenen

(1) Werden erstmals personenbezogene Daten für eigene Zwecke ohne Kenntnis des Betroffenen gespeichert, ist der Betroffene von der Speicherung, der Art der Daten, der Zweckbestimmung der Erhebung, Verarbeitung oder Nutzung und der Identität der verantwortlichen Stelle zu benachrichtigen. Werden personenbezogene Daten geschäftsmäßig zum Zweck der Übermittlung ohne Kenntnis des Betroffenen gespeichert, ist der Betroffene

von der erstmaligen Übermittlung und der Art der übermittelten Daten zu benachrichtigen. Der Betroffene ist in den Fällen der Sätze 1 und 2 auch über die Kategorien von Empfängern zu unterrichten, soweit er nach den Umständen des Einzelfalles nicht mit der Übermittlung an diese rechnen muss.

(2) Eine Pflicht zur Benachrichtigung besteht nicht, wenn

1. der Betroffene auf andere Weise Kenntnis von der Speicherung oder der Übermittlung erlangt hat,
2. die Daten nur deshalb gespeichert sind, weil sie aufgrund gesetzlicher, satzungsmäßiger oder vertraglicher Aufbewahrungsvorschriften nicht gelöscht werden dürfen oder ausschließlich der Datensicherung oder der Datenschutzkontrolle dienen und eine Benachrichtigung einen unverhältnismäßigen Aufwand erfordern würde,
3. die Daten nach einer Rechtsvorschrift oder ihrem Wesen nach, namentlich wegen des überwiegenden rechtlichen Interesses eines Dritten, geheimgehalten werden müssen,
4. die Speicherung oder Übermittlung durch Gesetz ausdrücklich vorgesehen ist,
5. die Speicherung oder Übermittlung für Zwecke der wissenschaftlichen Forschung erforderlich ist und eine Benachrichtigung einen unverhältnismäßigen Aufwand erfordern würde,
6. die zuständige öffentliche Stelle gegenüber der verantwortlichen Stelle festgestellt hat, dass das Bekanntwerden der Daten die öffentliche Sicherheit oder Ordnung gefährden oder sonst dem Wohle des Bundes oder eines Landes Nachteile bereiten würde,
7. die Daten für eigene Zwecke gespeichert sind und
   a) aus allgemein zugänglichen Quellen entnommen sind und eine Benachrichtigung wegen der Vielzahl der betroffenen Fälle unverhältnismäßig ist, oder
   b) die Benachrichtigung die Geschäftszwecke der verantwortlichen Stelle erheblich gefährden würde, es sei denn, dass das Interesse an der Benachrichtigung die Gefährdung überwiegt,
8. die Daten geschäftsmäßig zum Zweck der Übermittlung gespeichert sind und
   a) aus allgemein zugänglichen Quellen entnommen sind, soweit sie sich auf diejenigen Personen beziehen, die diese Daten veröffentlicht haben, oder
   b) es sich um listenmäßig oder sonst zusammengefasste Daten handelt (§ 29 Absatz 2 Satz 2)

   und eine Benachrichtigung wegen der Vielzahl der betroffenen Fälle unverhältnismäßig ist,
9. aus allgemein zugänglichen Quellen entnommene Daten geschäftsmäßig für Zwecke der Markt- oder Meinungsforschung gespeichert sind und eine Benachrichtigung wegen der Vielzahl der betroffenen Fälle unverhältnismäßig ist.

Die verantwortliche Stelle legt schriftlich fest, unter welchen Voraussetzungen von einer Benachrichtigung nach Satz 1 Nr. 2 bis 7 abgesehen wird.

§ 34 Auskunft an den Betroffenen

(1) Die verantwortliche Stelle hat dem Betroffenen auf Verlangen Auskunft zu erteilen über

1. die zu seiner Person gespeicherten Daten, auch soweit sie sich auf die Herkunft dieser Daten beziehen,
2. den Empfänger oder die Kategorien von Empfängern, an die Daten weitergegeben werden, und
3. den Zweck der Speicherung.

Der Betroffene soll die Art der personenbezogenen Daten, über die Auskunft erteilt werden soll, näher bezeichnen. Werden die personenbezogenen Daten geschäftsmäßig zum Zweck der Übermittlung gespeichert, ist Auskunft über die Herkunft und die Empfänger auch dann zu erteilen, wenn diese Angaben nicht gespeichert sind. Die Auskunft über die Herkunft und die Empfänger kann verweigert werden, soweit das Interesse an der Wahrung des Geschäftsgeheimnisses gegenüber dem Informationsinteresse des Betroffenen überwiegt.

(1a) Im Fall des § 28 Absatz 3 Satz 4 hat die übermittelnde Stelle die Herkunft der Daten und den Empfänger für die Dauer von zwei Jahren nach der Übermittlung zu speichern und dem Betroffenen auf Verlangen Auskunft über die Herkunft der Daten und den Empfänger zu erteilen. Satz 1 gilt entsprechend für den Empfänger.

(2) Im Fall des § 28b hat die für die Entscheidung verantwortliche Stelle dem Betroffenen auf Verlangen Auskunft zu erteilen über

1. die innerhalb der letzten sechs Monate vor dem Zugang des Auskunftsverlangens erhobenen oder erstmalig gespeicherten Wahrscheinlichkeitswerte,
2. die zur Berechnung der Wahrscheinlichkeitswerte genutzten Datenarten und
3. das Zustandekommen und die Bedeutung der Wahrscheinlichkeitswerte einzelfallbezogen und nachvollziehbar in allgemein verständlicher Form.

Satz 1 gilt entsprechend, wenn die für die Entscheidung verantwortliche Stelle

1. die zur Berechnung der Wahrscheinlichkeitswerte genutzten Daten ohne Personenbezug speichert, den Personenbezug aber bei der Berechnung herstellt oder
2. bei einer anderen Stelle gespeicherte Daten nutzt.

Hat eine andere als die für die Entscheidung verantwortliche Stelle

1. den Wahrscheinlichkeitswert oder
2. einen Bestandteil des Wahrscheinlichkeitswerts

berechnet, hat sie die insoweit zur Erfüllung der Auskunftsansprüche nach den Sätzen 1 und 2 erforderlichen Angaben auf Verlangen der für die Entscheidung verantwortlichen Stelle an diese zu übermitteln. Im Fall des Satzes 3 Nr. 1 hat die für die Entscheidung verantwortliche Stelle den Betroffenen zur Geltendmachung seiner Auskunftsansprüche unter Angabe des Namens und der Anschrift der anderen Stelle sowie der zur Bezeichnung des Einzelfalls notwendigen Angaben unverzüglich an diese zu verweisen, soweit sie die Auskunft nicht selbst erteilt. In diesem Fall hat die andere Stelle, die den Wahrscheinlichkeitswert berechnet hat, die Auskunftsansprüche nach den Sätzen 1 und 2 gegenüber dem Betroffenen unentgeltlich zu erfüllen. Die Pflicht der für die Berechnung des Wahrscheinlichkeitswerts verantwortlichen Stelle nach Satz 3 entfällt, soweit die für die Entscheidung verantwortliche Stelle von ihrem Recht nach Satz 4 Gebrauch macht.

(3) Eine Stelle, die geschäftsmäßig personenbezogene Daten zum Zweck der Übermittlung speichert, hat dem Betroffenen auf Verlangen Auskunft über die zu seiner Person gespeicherten Daten zu erteilen, auch wenn sie weder automatisiert verarbeitet werden noch in einer nicht automatisierten Datei gespeichert sind. Dem Betroffenen ist auch Auskunft zu erteilen über Daten, die

1. gegenwärtig noch keinen Personenbezug aufweisen, bei denen ein solcher aber im Zusammenhang mit der Auskunftserteilung von der verantwortlichen Stelle hergestellt werden soll,
2. die verantwortliche Stelle nicht speichert, aber zum Zweck der Auskunftserteilung nutzt.

Die Auskunft über die Herkunft und die Empfänger kann verweigert werden, soweit das Interesse an der Wahrung des Geschäftsgeheimnisses gegenüber dem Informationsinteresse des Betroffenen überwiegt.

(4) Eine Stelle, die geschäftsmäßig personenbezogene Daten zum Zweck der Übermittlung erhebt, speichert oder verändert, hat dem Betroffenen auf Verlangen Auskunft zu erteilen über

1. die innerhalb der letzten zwölf Monate vor dem Zugang des Auskunftsverlangens übermittelten Wahrscheinlichkeitswerte für ein bestimmtes zukünftiges Verhalten des Betroffenen sowie die Namen und letztbekannten Anschriften der Dritten, an die die Werte übermittelt worden sind,
2. die Wahrscheinlichkeitswerte, die sich zum Zeitpunkt des Auskunftsverlangens nach den von der Stelle zur Berechnung angewandten Verfahren ergeben,
3. die zur Berechnung der Wahrscheinlichkeitswerte nach den Nummern 1 und 2 genutzten Datenarten sowie
4. das Zustandekommen und die Bedeutung der Wahrscheinlichkeitswerte einzelfallbezogen und nachvollziehbar in allgemein verständlicher Form.

Satz 1 gilt entsprechend, wenn die verantwortliche Stelle

1. die zur Berechnung des Wahrscheinlichkeitswerts genutzten Daten ohne Personenbezug speichert, den Personenbezug aber bei der Berechnung herstellt oder
2. bei einer anderen Stelle gespeicherte Daten nutzt.

(5) Die nach den Absätzen 1a bis 4 zum Zweck der Auskunftserteilung an den Betroffenen gespeicherten Daten dürfen nur für diesen Zweck sowie für Zwecke der Datenschutzkontrolle verwendet werden; für andere Zwecke sind sie zu sperren.

(6) Die Auskunft ist auf Verlangen in Textform zu erteilen, soweit nicht wegen der besonderen Umstände eine andere Form der Auskunftserteilung angemessen ist.

(7) Eine Pflicht zur Auskunftserteilung besteht nicht, wenn der Betroffene nach § 33 Abs. 2 Satz 1 Nr. 2, 3 und 5 bis 7 nicht zu benachrichtigen ist.

(8) Die Auskunft ist unentgeltlich. Werden die personenbezogenen Daten geschäftsmäßig zum Zweck der Übermittlung gespeichert, kann der Betroffene einmal je Kalenderjahr eine unentgeltliche Auskunft in Textform verlangen. Für jede weitere Auskunft kann ein Entgelt verlangt werden, wenn der Betroffene die Auskunft gegenüber Dritten zu wirtschaftlichen Zwecken nutzen kann. Das Entgelt darf über die durch die Auskunftserteilung entstandenen unmittelbar zurechenbaren Kosten nicht hinausgehen. Ein Entgelt kann nicht verlangt werden, wenn

1. besondere Umstände die Annahme rechtfertigen, dass Daten unrichtig oder unzulässig gespeichert werden, oder
2. die Auskunft ergibt, dass die Daten nach § 35 Abs. 1 zu berichtigen oder nach § 35 Abs. 2 Satz 2 Nr. 1 zu löschen sind.

(9) Ist die Auskunftserteilung nicht unentgeltlich, ist dem Betroffenen die Möglichkeit zu geben, sich im Rahmen seines Auskunftsanspruchs persönlich Kenntnis über die ihn betreffenden Daten zu verschaffen. Er ist hierauf hinzuweisen.

§ 35 Berichtigung, Löschung und Sperrung von Daten

(1) Personenbezogene Daten sind zu berichtigen, wenn sie unrichtig sind. Geschätzte Daten sind als solche deutlich zu kennzeichnen.

(2) Personenbezogene Daten können außer in den Fällen des Absatzes 3 Nr. 1 und 2 jederzeit gelöscht werden. Personenbezogene Daten sind zu löschen, wenn

1. ihre Speicherung unzulässig ist,
2. es sich um Daten über die rassische oder ethnische Herkunft, politische Meinungen, religiöse oder philosophische Überzeugungen, Gewerkschaftszugehörigkeit, Gesundheit, Sexualleben, strafbare Handlungen oder Ordnungswidrigkeiten handelt und ihre Richtigkeit von der verantwortlichen Stelle nicht bewiesen werden kann,
3. sie für eigene Zwecke verarbeitet werden, sobald ihre Kenntnis für die Erfüllung des Zwecks der Speicherung nicht mehr erforderlich ist, oder
4. sie geschäftsmäßig zum Zweck der Übermittlung verarbeitet werden und eine Prüfung jeweils am Ende des vierten, soweit es sich um Daten über erledigte Sachverhalte handelt und der Betroffene der Löschung nicht widerspricht, am Ende des dritten Kalenderjahres beginnend mit dem Kalenderjahr, das der erstmaligen Speicherung folgt, ergibt, dass eine längerwährende Speicherung nicht erforderlich ist.

Personenbezogene Daten, die auf der Grundlage von § 28a Abs. 2 Satz 1 oder § 29 Abs. 1 Satz 1 Nr. 3 gespeichert werden, sind nach Beendigung des Vertrages auch zu löschen, wenn der Betroffene dies verlangt.

(3) An die Stelle einer Löschung tritt eine Sperrung, soweit

1. im Fall des Absatzes 2 Satz 2 Nr. 3 einer Löschung gesetzliche, satzungsmäßige oder vertragliche Aufbewahrungsfristen entgegenstehen,
2. Grund zu der Annahme besteht, dass durch eine Löschung schutzwürdige Interessen des Betroffenen beeinträchtigt würden, oder
3. eine Löschung wegen der besonderen Art der Speicherung nicht oder nur mit unverhältnismäßig hohem Aufwand möglich ist.

(4) Personenbezogene Daten sind ferner zu sperren, soweit ihre Richtigkeit vom Betroffenen bestritten wird und sich weder die Richtigkeit noch die Unrichtigkeit feststellen lässt.

(4a) Die Tatsache der Sperrung darf nicht übermittelt werden.

(5) Personenbezogene Daten dürfen nicht für eine automatisierte Verarbeitung oder Verarbeitung in nicht automatisierten Dateien erhoben, verarbeitet oder genutzt werden, soweit der Betroffene dieser bei der verantwortlichen Stelle widerspricht und eine Prüfung ergibt, dass das schutzwürdige Interesse des Betroffenen wegen seiner besonderen persönlichen Situation das Interesse der verantwortlichen Stelle an dieser Erhebung,

Verarbeitung oder Nutzung überwiegt. Satz 1 gilt nicht, wenn eine Rechtsvorschrift zur Erhebung, Verarbeitung oder Nutzung verpflichtet.

(6) Personenbezogene Daten, die unrichtig sind oder deren Richtigkeit bestritten wird, müssen bei der geschäftsmäßigen Datenspeicherung zum Zweck der Übermittlung außer in den Fällen des Absatzes 2 Nr. 2 nicht berichtigt, gesperrt oder gelöscht werden, wenn sie aus allgemein zugänglichen Quellen entnommen und zu Dokumentationszwecken gespeichert sind. Auf Verlangen des Betroffenen ist diesen Daten für die Dauer der Speicherung seine Gegendarstellung beizufügen. Die Daten dürfen nicht ohne diese Gegendarstellung übermittelt werden.

(7) Von der Berichtigung unrichtiger Daten, der Sperrung bestrittener Daten sowie der Löschung oder Sperrung wegen Unzulässigkeit der Speicherung sind die Stellen zu verständigen, denen im Rahmen einer Datenübermittlung diese Daten zur Speicherung weitergegeben wurden, wenn dies keinen unverhältnismäßigen Aufwand erfordert und schutzwürdige Interessen des Betroffenen nicht entgegenstehen.

(8) Gesperrte Daten dürfen ohne Einwilligung des Betroffenen nur übermittelt oder genutzt werden, wenn

1. es zu wissenschaftlichen Zwecken, zur Behebung einer bestehenden Beweisnot oder aus sonstigen im überwiegenden Interesse der verantwortlichen Stelle oder eines Dritten liegenden Gründen unerlässlich ist und
2. die Daten hierfür übermittelt oder genutzt werden dürften, wenn sie nicht gesperrt wären.

Dritter Unterabschnitt
Aufsichtsbehörde

§§ 36 und 37 (weggefallen)

-

§ 38 Aufsichtsbehörde

(1) Die Aufsichtsbehörde kontrolliert die Ausführung dieses Gesetzes sowie anderer Vorschriften über den Datenschutz, soweit diese die automatisierte Verarbeitung personenbezogener Daten oder die Verarbeitung oder Nutzung personenbezogener Daten in oder aus nicht automatisierten Dateien regeln einschließlich des Rechts der Mitgliedstaaten in den Fällen des § 1 Abs. 5. Sie berät und unterstützt die Beauftragten für den Datenschutz und die verantwortlichen Stellen mit Rücksicht auf deren typische Bedürfnisse. Die Aufsichtsbehörde darf die von ihr gespeicherten Daten nur für Zwecke der Aufsicht verarbeiten und nutzen; § 14 Abs. 2 Nr. 1 bis 3, 6 und 7 gilt entsprechend. Insbesondere darf die Aufsichtsbehörde zum Zweck der Aufsicht Daten an andere Aufsichtsbehörden übermitteln. Sie leistet den Aufsichtsbehörden anderer Mitgliedstaaten der Europäischen Union auf Ersuchen ergänzende Hilfe (Amtshilfe). Stellt die Aufsichtsbehörde einen Verstoß gegen dieses Gesetz oder andere Vorschriften über den Datenschutz fest, so ist sie befugt, die Betroffenen hierüber zu unterrichten, den Verstoß bei den für die Verfolgung oder Ahndung zuständigen Stellen anzuzeigen sowie bei schwerwiegenden Verstößen die Gewerbeaufsichtsbehörde zur Durchführung gewerberechtlicher Maßnahmen zu unterrichten. Sie veröffentlicht regelmäßig, spätestens alle zwei Jahre, einen Tätigkeitsbericht. § 21 Satz 1 und § 23 Abs. 5 Satz 4 bis 7 gelten entsprechend.

(2) Die Aufsichtsbehörde führt ein Register der nach § 4d meldepflichtigen automatisierten Verarbeitungen mit den Angaben nach § 4e Satz 1. Das Register kann von jedem eingesehen werden. Das Einsichtsrecht erstreckt sich nicht auf die Angaben nach § 4e Satz 1 Nr. 9 sowie auf die Angabe der zugriffsberechtigten Personen.

(3) Die der Kontrolle unterliegenden Stellen sowie die mit deren Leitung beauftragten Personen haben der Aufsichtsbehörde auf Verlangen die für die Erfüllung ihrer Aufgaben erforderlichen Auskünfte unverzüglich zu erteilen. Der Auskunftspflichtige kann die Auskunft auf solche Fragen verweigern, deren Beantwortung ihn selbst oder einen der in § 383 Abs. 1 Nr. 1 bis 3 der Zivilprozessordnung bezeichneten Angehörigen der Gefahr strafgerichtlicher Verfolgung oder eines Verfahrens nach dem Gesetz über Ordnungswidrigkeiten aussetzen würde. Der Auskunftspflichtige ist darauf hinzuweisen.

(4) Die von der Aufsichtsbehörde mit der Kontrolle beauftragten Personen sind befugt, soweit es zur Erfüllung der der Aufsichtsbehörde übertragenen Aufgaben erforderlich ist, während der Betriebs- und Geschäftszeiten Grundstücke und Geschäftsräume der Stelle zu betreten und dort Prüfungen und Besichtigungen vorzunehmen. Sie können geschäftliche Unterlagen, insbesondere die Übersicht nach § 4g Abs. 2 Satz 1 sowie die gespeicherten

personenbezogenen Daten und die Datenverarbeitungsprogramme, einsehen. § 24 Abs. 6 gilt entsprechend. Der Auskunftspflichtige hat diese Maßnahmen zu dulden.

(5) Zur Gewährleistung der Einhaltung dieses Gesetzes und anderer Vorschriften über den Datenschutz kann die Aufsichtsbehörde Maßnahmen zur Beseitigung festgestellter Verstöße bei der Erhebung, Verarbeitung oder Nutzung personenbezogener Daten oder technischer oder organisatorischer Mängel anordnen. Bei schwerwiegenden Verstößen oder Mängeln, insbesondere solchen, die mit einer besonderen Gefährdung des Persönlichkeitsrechts verbunden sind, kann sie die Erhebung, Verarbeitung oder Nutzung oder den Einsatz einzelner Verfahren untersagen, wenn die Verstöße oder Mängel entgegen der Anordnung nach Satz 1 und trotz der Verhängung eines Zwangsgeldes nicht in angemessener Zeit beseitigt werden. Sie kann die Abberufung des Beauftragten für den Datenschutz verlangen, wenn er die zur Erfüllung seiner Aufgaben erforderliche Fachkunde und Zuverlässigkeit nicht besitzt.

(6) Die Landesregierungen oder die von ihnen ermächtigten Stellen bestimmen die für die Kontrolle der Durchführung des Datenschutzes im Anwendungsbereich dieses Abschnittes zuständigen Aufsichtsbehörden.

(7) Die Anwendung der Gewerbeordnung auf die den Vorschriften dieses Abschnittes unterliegenden Gewerbebetriebe bleibt unberührt.

§ 38a Verhaltensregeln zur Förderung der Durchführung datenschutzrechtlicher Regelungen

(1) Berufsverbände und andere Vereinigungen, die bestimmte Gruppen von verantwortlichen Stellen vertreten, können Entwürfe für Verhaltensregeln zur Förderung der Durchführung von datenschutzrechtlichen Regelungen der zuständigen Aufsichtsbehörde unterbreiten.

(2) Die Aufsichtsbehörde überprüft die Vereinbarkeit der ihr unterbreiteten Entwürfe mit dem geltenden Datenschutzrecht.

Vierter Abschnitt
Sondervorschriften

§ 39 Zweckbindung bei personenbezogenen Daten, die einem Berufs- oder besonderen Amtsgeheimnis unterliegen

(1) Personenbezogene Daten, die einem Berufs- oder besonderen Amtsgeheimnis unterliegen und die von der zur Verschwiegenheit verpflichteten Stelle in Ausübung ihrer Berufs- oder Amtspflicht zur Verfügung gestellt worden sind, dürfen von der verantwortlichen Stelle nur für den Zweck verarbeitet oder genutzt werden, für den sie sie erhalten hat. In die Übermittlung an eine nicht-öffentliche Stelle muss die zur Verschwiegenheit verpflichtete Stelle einwilligen.

(2) Für einen anderen Zweck dürfen die Daten nur verarbeitet oder genutzt werden, wenn die Änderung des Zwecks durch besonderes Gesetz zugelassen ist.

§ 40 Verarbeitung und Nutzung personenbezogener Daten durch Forschungseinrichtungen

(1) Für Zwecke der wissenschaftlichen Forschung erhobene oder gespeicherte personenbezogene Daten dürfen nur für Zwecke der wissenschaftlichen Forschung verarbeitet oder genutzt werden.

(2) Die personenbezogenen Daten sind zu anonymisieren, sobald dies nach dem Forschungszweck möglich ist. Bis dahin sind die Merkmale gesondert zu speichern, mit denen Einzelangaben über persönliche oder sachliche Verhältnisse einer bestimmten oder bestimmbaren Person zugeordnet werden können. Sie dürfen mit den Einzelangaben nur zusammengeführt werden, soweit der Forschungszweck dies erfordert.

(3) Die wissenschaftliche Forschung betreibenden Stellen dürfen personenbezogene Daten nur veröffentlichen, wenn

1. der Betroffene eingewilligt hat oder
2. dies für die Darstellung von Forschungsergebnissen über Ereignisse der Zeitgeschichte unerlässlich ist.

§ 41 Erhebung, Verarbeitung und Nutzung personenbezogener Daten durch die Medien

(1) Die Länder haben in ihrer Gesetzgebung vorzusehen, dass für die Erhebung, Verarbeitung und Nutzung personenbezogener Daten von Unternehmen und Hilfsunternehmen der Presse ausschließlich zu eigenen journalistisch-redaktionellen oder literarischen Zwecken den Vorschriften der §§ 5, 9 und 38a entsprechende Regelungen einschließlich einer hierauf bezogenen Haftungsregelung entsprechend § 7 zur Anwendung kommen.

(2) Führt die journalistisch-redaktionelle Erhebung, Verarbeitung oder Nutzung personenbezogener Daten durch die Deutsche Welle zur Veröffentlichung von Gegendarstellungen des Betroffenen, so sind diese Gegendarstellungen zu den gespeicherten Daten zu nehmen und für dieselbe Zeitdauer aufzubewahren wie die Daten selbst.

(3) Wird jemand durch eine Berichterstattung der Deutschen Welle in seinem Persönlichkeitsrecht beeinträchtigt, so kann er Auskunft über die der Berichterstattung zugrunde liegenden, zu seiner Person gespeicherten Daten verlangen. Die Auskunft kann nach Abwägung der schutzwürdigen Interessen der Beteiligten verweigert werden, soweit

1. aus den Daten auf Personen, die bei der Vorbereitung, Herstellung oder Verbreitung von Rundfunksendungen berufsmäßig journalistisch mitwirken oder mitgewirkt haben, geschlossen werden kann,
2. aus den Daten auf die Person des Einsenders oder des Gewährsträgers von Beiträgen, Unterlagen und Mitteilungen für den redaktionellen Teil geschlossen werden kann,
3. durch die Mitteilung der recherchierten oder sonst erlangten Daten die journalistische Aufgabe der Deutschen Welle durch Ausforschung des Informationsbestandes beeinträchtigt würde.

Der Betroffene kann die Berichtigung unrichtiger Daten verlangen.

(4) Im Übrigen gelten für die Deutsche Welle von den Vorschriften dieses Gesetzes die §§ 5, 7, 9 und 38a. Anstelle der §§ 24 bis 26 gilt § 42, auch soweit es sich um Verwaltungsangelegenheiten handelt.

§ 42 Datenschutzbeauftragter der Deutschen Welle

(1) Die Deutsche Welle bestellt einen Beauftragten für den Datenschutz, der an die Stelle des Bundesbeauftragten für den Datenschutz und die Informationsfreiheit tritt. Die Bestellung erfolgt auf Vorschlag des Intendanten durch den Verwaltungsrat für die Dauer von vier Jahren, wobei Wiederbestellungen zulässig sind. Das Amt eines Beauftragten für den Datenschutz kann neben anderen Aufgaben innerhalb der Rundfunkanstalt wahrgenommen werden.

(2) Der Beauftragte für den Datenschutz kontrolliert die Einhaltung der Vorschriften dieses Gesetzes sowie anderer Vorschriften über den Datenschutz. Er ist in Ausübung dieses Amtes unabhängig und nur dem Gesetz unterworfen. Im Übrigen untersteht er der Dienst- und Rechtsaufsicht des Verwaltungsrates.

(3) Jedermann kann sich entsprechend § 21 Satz 1 an den Beauftragten für den Datenschutz wenden.

(4) Der Beauftragte für den Datenschutz erstattet den Organen der Deutschen Welle alle zwei Jahre, erstmals zum 1. Januar 1994 einen Tätigkeitsbericht. Er erstattet darüber hinaus besondere Berichte auf Beschluss eines Organes der Deutschen Welle. Die Tätigkeitsberichte übermittelt der Beauftragte auch an den Bundesbeauftragten für den Datenschutz und die Informationsfreiheit.

(5) Weitere Regelungen entsprechend den §§ 23 bis 26 trifft die Deutsche Welle für ihren Bereich. Die §§ 4f und 4g bleiben unberührt.

§ 42a Informationspflicht bei unrechtmäßiger Kenntniserlangung von Daten

Stellt eine nichtöffentliche Stelle im Sinne des § 2 Absatz 4 oder eine öffentliche Stelle nach § 27 Absatz 1 Satz 1 Nummer 2 fest, dass bei ihr gespeicherte

1. besondere Arten personenbezogener Daten (§ 3 Absatz 9),
2. personenbezogene Daten, die einem Berufsgeheimnis unterliegen,
3. personenbezogene Daten, die sich auf strafbare Handlungen oder Ordnungswidrigkeiten oder den Verdacht strafbarer Handlungen oder Ordnungswidrigkeiten beziehen, oder

4. personenbezogene Daten zu Bank- oder Kreditkartenkonten

unrechtmäßig übermittelt oder auf sonstige Weise Dritten unrechtmäßig zur Kenntnis gelangt sind, und drohen schwerwiegende Beeinträchtigungen für die Rechte oder schutzwürdigen Interessen der Betroffenen, hat sie dies nach den Sätzen 2 bis 5 unverzüglich der zuständigen Aufsichtsbehörde sowie den Betroffenen mitzuteilen. Die Benachrichtigung des Betroffenen muss unverzüglich erfolgen, sobald angemessene Maßnahmen zur Sicherung der Daten ergriffen worden oder nicht unverzüglich erfolgt sind und die Strafverfolgung nicht mehr gefährdet wird. Die Benachrichtigung der Betroffenen muss eine Darlegung der Art der unrechtmäßigen Kenntniserlangung und Empfehlungen für Maßnahmen zur Minderung möglicher nachteiliger Folgen enthalten. Die Benachrichtigung der zuständigen Aufsichtsbehörde muss zusätzlich eine Darlegung möglicher nachteiliger Folgen der unrechtmäßigen Kenntniserlangung und der von der Stelle daraufhin ergriffenen Maßnahmen enthalten. Soweit die Benachrichtigung der Betroffenen einen unverhältnismäßigen Aufwand erfordern würde, insbesondere aufgrund der Vielzahl der betroffenen Fälle, tritt an ihre Stelle die Information der Öffentlichkeit durch Anzeigen, die mindestens eine halbe Seite umfassen, in mindestens zwei bundesweit erscheinenden Tageszeitungen oder durch eine andere, in ihrer Wirksamkeit hinsichtlich der Information der Betroffenen gleich geeignete Maßnahme. Eine Benachrichtigung, die der Benachrichtigungspflichtige erteilt hat, darf in einem Strafverfahren oder in einem Verfahren nach dem Gesetz über Ordnungswidrigkeiten gegen ihn oder einen in § 52 Absatz 1 der Strafprozessordnung bezeichneten Angehörigen des Benachrichtigungspflichtigen nur mit Zustimmung des Benachrichtigungspflichtigen verwendet werden.

Fünfter Abschnitt
Schlussvorschriften

§ 43 Bußgeldvorschriften

(1) Ordnungswidrig handelt, wer vorsätzlich oder fahrlässig

1. entgegen § 4d Abs. 1, auch in Verbindung mit § 4e Satz 2, eine Meldung nicht, nicht richtig, nicht vollständig oder nicht rechtzeitig macht,
2. entgegen § 4f Abs. 1 Satz 1 oder 2, jeweils auch in Verbindung mit Satz 3 und 6, einen Beauftragten für den Datenschutz nicht, nicht in der vorgeschriebenen Weise oder nicht rechtzeitig bestellt,

2a. entgegen § 10 Absatz 4 Satz 3 nicht gewährleistet, dass die Datenübermittlung festgestellt und überprüft werden kann,

2b. entgegen § 11 Absatz 2 Satz 2 einen Auftrag nicht richtig, nicht vollständig oder nicht in der vorgeschriebenen Weise erteilt oder entgegen § 11 Absatz 2 Satz 4 sich nicht vor Beginn der Datenverarbeitung von der Einhaltung der beim Auftragnehmer getroffenen technischen und organisatorischen Maßnahmen überzeugt,

3. entgegen § 28 Abs. 4 Satz 2 den Betroffenen nicht, nicht richtig oder nicht rechtzeitig unterrichtet oder nicht sicherstellt, dass der Betroffene Kenntnis erhalten kann,

3a. entgegen § 28 Absatz 4 Satz 4 eine strengere Form verlangt,

4. entgegen § 28 Abs. 5 Satz 2 personenbezogene Daten übermittelt oder nutzt,

4a. entgegen § 28a Abs. 3 Satz 1 eine Mitteilung nicht, nicht richtig, nicht vollständig oder nicht rechtzeitig macht,

5. entgegen § 29 Abs. 2 Satz 3 oder 4 die dort bezeichneten Gründe oder die Art und Weise ihrer glaubhaften Darlegung nicht aufzeichnet,

6. entgegen § 29 Abs. 3 Satz 1 personenbezogene Daten in elektronische oder gedruckte Adress-, Rufnummern-, Branchen- oder vergleichbare Verzeichnisse aufnimmt,

7. entgegen § 29 Abs. 3 Satz 2 die Übernahme von Kennzeichnungen nicht sicherstellt,

7a. entgegen § 29 Abs. 6 ein Auskunftsverlangen nicht richtig behandelt,

7b. entgegen § 29 Abs. 7 Satz 1 einen Verbraucher nicht, nicht richtig, nicht vollständig oder nicht rechtzeitig unterrichtet,

8. entgegen § 33 Abs. 1 den Betroffenen nicht, nicht richtig oder nicht vollständig benachrichtigt,

8a. entgegen § 34 Absatz 1 Satz 1, auch in Verbindung mit Satz 3, entgegen § 34 Absatz 1a, entgegen § 34 Absatz 2 Satz 1, auch in Verbindung mit Satz 2, oder entgegen § 34 Absatz 2 Satz 5, Absatz 3 Satz 1

oder Satz 2 oder Absatz 4 Satz 1, auch in Verbindung mit Satz 2, eine Auskunft nicht, nicht richtig, nicht vollständig oder nicht rechtzeitig erteilt oder entgegen § 34 Absatz 1a Daten nicht speichert,

8b. entgegen § 34 Abs. 2 Satz 3 Angaben nicht, nicht richtig, nicht vollständig oder nicht rechtzeitig übermittelt,

8c. entgegen § 34 Abs. 2 Satz 4 den Betroffenen nicht oder nicht rechtzeitig an die andere Stelle verweist,

9. entgegen § 35 Abs. 6 Satz 3 Daten ohne Gegendarstellung übermittelt,

10. entgegen § 38 Abs. 3 Satz 1 oder Abs. 4 Satz 1 eine Auskunft nicht, nicht richtig, nicht vollständig oder nicht rechtzeitig erteilt oder eine Maßnahme nicht duldet oder

11. einer vollziehbaren Anordnung nach § 38 Abs. 5 Satz 1 zuwiderhandelt.

(2) Ordnungswidrig handelt, wer vorsätzlich oder fahrlässig

1. unbefugt personenbezogene Daten, die nicht allgemein zugänglich sind, erhebt oder verarbeitet,

2. unbefugt personenbezogene Daten, die nicht allgemein zugänglich sind, zum Abruf mittels automatisierten Verfahrens bereithält,

3. unbefugt personenbezogene Daten, die nicht allgemein zugänglich sind, abruft oder sich oder einem anderen aus automatisierten Verarbeitungen oder nicht automatisierten Dateien verschafft,

4. die Übermittlung von personenbezogenen Daten, die nicht allgemein zugänglich sind, durch unrichtige Angaben erschleicht,

5. entgegen § 16 Abs. 4 Satz 1, § 28 Abs. 5 Satz 1, auch in Verbindung mit § 29 Abs. 4, § 39 Abs. 1 Satz 1 oder § 40 Abs. 1, die übermittelten Daten für andere Zwecke nutzt,

5a. entgegen § 28 Absatz 3b den Abschluss eines Vertrages von der Einwilligung des Betroffenen abhängig macht,

5b. entgegen § 28 Absatz 4 Satz 1 Daten für Zwecke der Werbung oder der Markt- oder Meinungsforschung verarbeitet oder nutzt,

6. entgegen § 30 Absatz 1 Satz 2, § 30a Absatz 3 Satz 3 oder § 40 Absatz 2 Satz 3 ein dort genanntes Merkmal mit einer Einzelangabe zusammenführt oder

7. entgegen § 42a Satz 1 eine Mitteilung nicht, nicht richtig, nicht vollständig oder nicht rechtzeitig macht.

(3) Die Ordnungswidrigkeit kann im Fall des Absatzes 1 mit einer Geldbuße bis zu fünfzigtausend Euro, in den Fällen des Absatzes 2 mit einer Geldbuße bis zu dreihunderttausend Euro geahndet werden. Die Geldbuße soll den wirtschaftlichen Vorteil, den der Täter aus der Ordnungswidrigkeit gezogen hat, übersteigen. Reichen die in Satz 1 genannten Beträge hierfür nicht aus, so können sie überschritten werden.

§ 44 Strafvorschriften

(1) Wer eine in § 43 Abs. 2 bezeichnete vorsätzliche Handlung gegen Entgelt oder in der Absicht, sich oder einen anderen zu bereichern oder einen anderen zu schädigen, begeht, wird mit Freiheitsstrafe bis zu zwei Jahren oder mit Geldstrafe bestraft.

(2) Die Tat wird nur auf Antrag verfolgt. Antragsberechtigt sind der Betroffene, die verantwortliche Stelle, der Bundesbeauftragte für den Datenschutz und die Informationsfreiheit und die Aufsichtsbehörde.

Sechster Abschnitt Übergangsvorschriften

§ 45 Laufende Verwendungen

Erhebungen, Verarbeitungen oder Nutzungen personenbezogener Daten, die am 23. Mai 2001 bereits begonnen haben, sind binnen drei Jahren nach diesem Zeitpunkt mit den Vorschriften dieses Gesetzes in Übereinstimmung zu bringen. Soweit Vorschriften dieses Gesetzes in Rechtsvorschriften außerhalb des Anwendungsbereichs der Richtlinie 95/46/EG des Europäischen Parlaments und des Rates vom 24. Oktober 1995 zum Schutz natürlicher Personen bei der Verarbeitung personenbezogener Daten und zum freien Datenverkehr zur Anwendung gelangen, sind Erhebungen, Verarbeitungen oder Nutzungen personenbezogener Daten, die am 23. Mai 2001

bereits begonnen haben, binnen fünf Jahren nach diesem Zeitpunkt mit den Vorschriften dieses Gesetzes in Übereinstimmung zu bringen.

§ 46 Weitergeltung von Begriffsbestimmungen

(1) Wird in besonderen Rechtsvorschriften des Bundes der Begriff Datei verwendet, ist Datei

1. eine Sammlung personenbezogener Daten, die durch automatisierte Verfahren nach bestimmten Merkmalen ausgewertet werden kann (automatisierte Datei), oder
2. jede sonstige Sammlung personenbezogener Daten, die gleichartig aufgebaut ist und nach bestimmten Merkmalen geordnet, umgeordnet und ausgewertet werden kann (nicht automatisierte Datei).

Nicht hierzu gehören Akten und Aktensammlungen, es sei denn, dass sie durch automatisierte Verfahren umgeordnet und ausgewertet werden können.

(2) Wird in besonderen Rechtsvorschriften des Bundes der Begriff Akte verwendet, ist Akte jede amtlichen oder dienstlichen Zwecken dienende Unterlage, die nicht dem Dateibegriff des Absatzes 1 unterfällt; dazu zählen auch Bild- und Tonträger. Nicht hierunter fallen Vorentwürfe und Notizen, die nicht Bestandteil eines Vorgangs werden sollen.

(3) Wird in besonderen Rechtsvorschriften des Bundes der Begriff Empfänger verwendet, ist Empfänger jede Person oder Stelle außerhalb der verantwortlichen Stelle. Empfänger sind nicht der Betroffene sowie Personen und Stellen, die im Inland, in einem anderen Mitgliedstaat der Europäischen Union oder in einem anderen Vertragsstaat des Abkommens über den Europäischen Wirtschaftsraum personenbezogene Daten im Auftrag erheben, verarbeiten oder nutzen.

§ 47 Übergangsregelung

Für die Verarbeitung und Nutzung vor dem 1. September 2009 erhobener oder gespeicherter Daten ist § 28 in der bis dahin geltenden Fassung weiter anzuwenden

1. für Zwecke der Markt- oder Meinungsforschung bis zum 31. August 2010,
2. für Zwecke der Werbung bis zum 31. August 2012.

§ 48 Bericht der Bundesregierung

Die Bundesregierung berichtet dem Bundestag

1. bis zum 31. Dezember 2012 über die Auswirkungen der §§ 30a und 42a,
2. bis zum 31. Dezember 2014 über die Auswirkungen der Änderungen der §§ 28 und 29.

Sofern sich aus Sicht der Bundesregierung gesetzgeberische Maßnahmen empfehlen, soll der Bericht einen Vorschlag enthalten.

Anlage (zu § 9 Satz 1)

(Fundstelle des Originaltextes: BGBl. I 2003, 88;
bzgl. der einzelnen Änderungen vgl. Fußnote)

Werden personenbezogene Daten automatisiert verarbeitet oder genutzt, ist die innerbehördliche oder innerbetriebliche Organisation so zu gestalten, dass sie den besonderen Anforderungen des Datenschutzes gerecht wird. Dabei sind insbesondere Maßnahmen zu treffen, die je nach der Art der zu schützenden personenbezogenen Daten oder Datenkategorien geeignet sind,

1. Unbefugten den Zutritt zu Datenverarbeitungsanlagen, mit denen personenbezogene Daten verarbeitet oder genutzt werden, zu verwehren (Zutrittskontrolle),
2. zu verhindern, dass Datenverarbeitungssysteme von Unbefugten genutzt werden können (Zugangskontrolle),
3. zu gewährleisten, dass die zur Benutzung eines Datenverarbeitungssystems Berechtigten ausschließlich auf die ihrer Zugriffsberechtigung unterliegenden Daten zugreifen können, und dass personenbezogene Daten bei der Verarbeitung, Nutzung und nach der Speicherung nicht unbefugt gelesen, kopiert, verändert oder entfernt werden können (Zugriffskontrolle),

4. zu gewährleisten, dass personenbezogene Daten bei der elektronischen Übertragung oder während ihres Transports oder ihrer Speicherung auf Datenträger nicht unbefugt gelesen, kopiert, verändert oder entfernt werden können, und dass überprüft und festgestellt werden kann, an welche Stellen eine Übermittlung personenbezogener Daten durch Einrichtungen zur Datenübertragung vorgesehen ist (Weitergabekontrolle),
5. zu gewährleisten, dass nachträglich überprüft und festgestellt werden kann, ob und von wem personenbezogene Daten in Datenverarbeitungssysteme eingegeben, verändert oder entfernt worden sind (Eingabekontrolle),
6. zu gewährleisten, dass personenbezogene Daten, die im Auftrag verarbeitet werden, nur entsprechend den Weisungen des Auftraggebers verarbeitet werden können (Auftragskontrolle),
7. zu gewährleisten, dass personenbezogene Daten gegen zufällige Zerstörung oder Verlust geschützt sind (Verfügbarkeitskontrolle),
8. zu gewährleisten, dass zu unterschiedlichen Zwecken erhobene Daten getrennt verarbeitet werden können.

Eine Maßnahme nach Satz 2 Nummer 2 bis 4 ist insbesondere die Verwendung von dem Stand der Technik entsprechenden Verschlüsselungsverfahren.
